# Supplementary material for: Programmable on-chip nonlinear photonics
Source: Nature. 2025 Oct 8;649(8096):330–7. doi: 10.1038/s41586-025-09620-9 (PMC12779561; doi:10.1038/s41586-025-09620-9)
Supplement: Supplementary file 1 — This file contains Supplementary Sections 1–13, including Supplementary Tables 1–3, Supplementary Figs. 1–40 and Supplementary references. [file 41586_2025_9620_MOESM1_ESM.pdf]

nature portfolio

https://doi.org/10.1038/s41586-025-09620-9

Supplementary information

Programmable on-chip nonlinear photonics

In the format provided by the authors and unedited

PROGRAMMABLE ON-CHIP NONLINEAR PHOTONICS: SUPPLEMENTARY INFORMATION

Ryotatsu Yanagimoto, Benjamin A. Ash, Mandar M. Sohoni, Martin M. Stein, Yiqi Zhao, Federico Presutti, Marc Jankowski, Logan G. Wright, Tatsuhiro Onodera, and Peter L. McMahon

TABLE OF CONTENTS

Introduction **2**
S1 Summary of device performance and potential for future improvements **2**
S2 Potential future applications of programmable nonlinear photonics **4**
A Highly programmable on-chip optical pulse shaper 4
B Programmable quantum frequency converter 6
C Widely tunable and highly efficient integrated light sources 6
D Programmable quantum light sources 8
E 100 %-yield QPM gratings 10
S3 Electrical properties of programmable nonlinear waveguides **10**
A Lumped-element circuit model 10
B Resolution limit imposed by electric field fringing 12
C Photoconductive core for smaller feature sizes 13
S4 Optical properties of a programmable nonlinear waveguide **14**
A Waveguide mode 14
B Phase-matching conditions 15
C Optical loss 16
D Optical damage threshold 21
S5 Experimental estimation of electric-field-induced $\chi^{(2)}$ nonlinearity **21**
S6 Model for SHG in a programmable nonlinear waveguide **23**
S7 Common parts of the experiment **24**
A Projector setup for programming illumination 24
B Electrical and optical coupling to the waveguide 25
S8 Programmable periodic poling for CW-pumped SHG **26**
A Calibration of the experimental setup 26
B Basic nonlinear-optical characterization of the device 28
C Real-time feedback to compensate for random walks in the pump wavelength 28
S9 Spectral engineering **29**
A Broadband SHG with manually designed QPM gratings 29
B In situ optimization of the QPM grating 31
C Real-time update of the QPM grating 32
S10 Spatial engineering **33**
A Calibration of the experimental setup 33
B Model for spatially engineered SHG 37
C Experimental results and simulations 38
S11 Spatio-spectral engineering **40**
S12 Need for experimental characterization of the electric-field-induced $\chi^{(2)}$ nonlinearity **41**
S13 Methods for inverse design **43**
A In silico inverse design 43
B Hybrid in situ–in silico inverse design 43
References **44**

INTRODUCTION

This document provides supplementary information related to the results presented in the main text. The sections in this document are organized into the following five categories:

- **Future prospects**: In Sec. S1, we summarize the current and potential future performance of the programmable nonlinear waveguides. In Sec. S2, we discuss potential applications of the platform based on quantitative metrics.
- **Characterization of the device**: These sections describe the physical parameters of the programmable nonlinear waveguides. The electrical and optical properties are summarized in Sec. S3 and Sec. S4, respectively. In Sec. S5, we provide details on how the programmable $\chi^{(2)}$ nonlinearity was estimated.
- **Model for the device operation**: In Sec. S6, we describe how to concisely model the nonlinear-optical behavior of the device for a given programming illumination.
- **Experimental details**: These sections provide additional information on the experimental results. The common parts of the setup are described in Sec. S7. The remaining sections cover CW-pumped SHG (Sec. S8), spectral engineering (Sec. S9), spatial engineering (Sec. S10), and spatio-spectral engineering (Sec. S11).
- **Theoretical background**: These sections offer supplementary theoretical context. In Sec. S12, we discuss the physics of electric-field-induced $\chi^{(2)}$ nonlinearity. In Sec. S13, we summarize methods for inverse designs in photonics.

S1. SUMMARY OF DEVICE PERFORMANCE AND POTENTIAL FOR FUTURE IMPROVEMENTS

Device type	Induced $\chi^{(2)}$ nonlinearity	Conversion efficiency η_{norm}	Bandwidth	Reference
Programmable planar waveguide	0.47 pm/V	5×10^{-5} %/W	700 GHz [a]	This work
Programmable channel waveguide	0.47 pm/V	2×10^{-3} %/W	700 GHz	This work
Channel waveguide	0.5 pm/V	5×10^{-3} %/W	70 THz [b]	Ref. [S1]
Channel waveguide	0.3 pm/V	5×10^{-2} %/W	300 GHz [c]	Ref. [S2]
Microring resonator	0.03 pm/V	47.6 %/W	260 MHz [d]	Ref. [S3]
Microring resonator	0.2 pm/V	2500 %/W	320 MHz [e]	Ref. [S4]
Microring resonator	Not provided	651 %/W	14 MHz [f]	Ref. [S5]
Microring resonator	0.03 pm/V	3.2 %/W	350 MHz [g]	Ref. [S6]
Microring resonator	0.022 pm/V	141 %/W	13 MHz [h]	Ref. [S7]

TABLE S1: Summary of the performance of electric-field induced $\chi^{(2)}$ nonlinear-optical device on SiN. For waveguides, the bandwidth is defined as the width of the SHG phase-matching function. For resonators, we refer to the linewidth of the fundamental harmonics. [a] 5.6 nm around the pump wavelength of 1562 nm. [b] 60 nm around the pump wavelength of 1560 nm. [c] 2.5 nm around the pump wavelength of 1544 nm. [d] $Q = 7.5 \times 10^5$ around 1550 nm. [e] $Q = 6.0 \times 10^5$ around 1560 nm. [f] $Q = 1.39 \times 10^7$ around 1560 nm. [g] $Q = 8 \times 10^6$ around 1060 nm. [h] $Q = 2.2 \times 10^7$ around 1063 nm.

To put the performance of the programmable nonlinear waveguides into context, in Table S1 we compare the performance of our devices with literature values reporting electric-field-induced second-harmonic generation (SHG) in SiN nanophotonics. In all cases except our work, the $\chi^{(2)}$ nonlinearity arose from the photogalvanic effect. As shown in the table, the programmable $\chi^{(2)}$ nonlinearity demonstrated in our work is comparable to, or even greater than, previously reported values. The apparently lower conversion efficiencies are primarily due to differences in device geometries, such as shorter propagation lengths and weaker transverse field confinement. As can be seen in the table, using a resonant structure can significantly enhance the conversion efficiency by recirculating both the pump and the generated SH light, at the cost of the bandwidth. As discussed in Sec. S2 C, forming a microring resonator with our programmable channel waveguide could achieve performance surpassing the current state of the art in nanophotonics, albeit with technical challenges in reducing propagation loss.

In table S2, we summarize the general performance of the programmable nonlinear planar waveguide experimentally demonstrated in this work and present projected performance for a future device with enhanced functionalities. Below, we further discuss the limitations of the current device and potential avenues for future improvements.

Performance	This work	Potential future device
Programmable $\chi^{(2)}$ nonlinearity (material)	0.47 pm/V (N-rich SiN [a])	22.7 pm/V (Si-rich SiN [b])<br>41 pm/V (Si [c])
Approximate bandgap wavelength (material)	400 nm (N-rich SiN [d])	600 nm (Si-rich SiN [e])<br>1100 nm (Si [f])
Update speed of nonlinearity	1 Hz [g]	200 Hz [h]
Area of programmable nonlinearity ($z \times x$)	7.2 mm × 4.5 mm [i]	1.6 cm × 2.9 cm [j]
Smallest programmable feature size	7.5 µm [k]	1 µm [l]
Optical loss	1 dB/cm ∼ 5 dB/cm [m]	< 1 dB/cm [n]

TABLE S2: The table summarizes the performance of the programmable nonlinear planar waveguide demonstrated in this work, as well as that of a potential future device. [a] See Sec. S5. [b] Value reported in Ref. [S8]. [c] Value reported in Ref. [S9]. [d] Inferred from ellipsometry data. [e] Corresponding to the value of ∼ 2 eV reported for the highest-index SRN film in Ref. [S10]. [f] Value reported in Ref. [S11]. [g] Speed limit imposed by the update speed of the SLM. [h] A high-speed SLM can achieve update speeds exceeding 1 kHz. The speed limit is set by the RC time constant of the device (see Sec. S3 A). We assume a 10× increase in the bright-state photoconductivity. [i] The current SLM has 1920 × 1200 pixels, with each pixel mapping to 3.772 µm spot (see Sec. S7 A). [j] Assuming a high-resolution SLM, with 8K resolution (7680 × 4320 pixels) [S12]. We assume that each pixel of the SLM maps to a 3.772 µm spot on the programmable waveguide. We can further increase the programmable area by combining illumination from multiple SLMs. In this case, the upper bound is imposed by limitations in fabrication, e.g., the size of a wafer. [k] Limit imposed by the fringing of electric fields (see Sec. S3 B). [l] Assuming a programmable nonlinear waveguide with a 1 µm-thick photoconductive core (see Sec. S3 C). [m] The loss is maximal around 1520 nm, while the typical loss at other wavelengths is 1 dB/cm (see Sec. S4 C). [n] Various materials compatible with programmable nonlinear waveguides have demonstrated this level of loss [S13–S15]. As discussed in Sec. S4 C 4, annealing of SiN reduces the propagation loss. We observed 0.4 dB/cm on annealed PECVD SiN.

A most important limitation of the current prototype device is the low value of programmable nonlinearity, which was measured to be $\chi^{(2)} = 0.47$ pm/V. Fortunately, we expect there are various routes to improve the nonlinearity. For instance, the electric-field contrast within the core layer could be improved by using a thicker photoconductor layer, which reduces the spatially uniform background nonlinearity that does not contribute to nonlinear-optical processes, thereby increasing the dynamic range of the programmable nonlinearity. Electrical characterization of the device suggests that a thicker photoconductor with higher photoconductivity could increase the programmable nonlinearity by up to a factor of 2.3, which would result in a maximum programmable $\chi^{(2)}$ of 1.1 pm/V (see Sec. S5). We chose plasma-enhanced chemical vapor deposition (PECVD)-grown low-index (i.e., nitrogen-rich) SiN as our core material (see the Methods section) because of its commercial availability and large bandgap (making it suitable for SHG pumped at ∼1.55 µm). However, other materials can achieve substantially higher electric-field-induced $\chi^{(2)}$ nonlinearities, such as 41 pm/V with Si [S9] and 22.7 pm/V with silicon-rich silicon nitride (SRN) [S8]. These materials could increase the magnitude of programmable $\chi^{(2)}$ nonlinearity to near that of conventional state-of-the-art materials like lithium niobate (∼50 pm/V). However, the bandgaps of Si and SRN are smaller than that of SiN, which limits their operation to longer wavelengths of light. The potential use of Si or SRN as the waveguide core material provide a promising path for developing high-nonlinearity programmable nonlinear waveguides for wavelengths longer than ∼1 µm, e.g., for mid-infrared applications [S16]. What are the prospects for making high-nonlinearity devices with transparency windows covering some or all of the visible wavelengths too? A suitable material for the waveguide core should have a large bandgap, high breakdown voltage, and low optical loss, as well as a high $\chi^{(3)}$. Promising candidates for evaluation include diamond, silicon carbide, and aluminum nitride. The relevant $\chi^{(3)}$ tensor element for evaluating the magnitude of possible electric-field-induced $\chi^{(2)}$ in a material is surprisingly understudied in the literature (see Sec. S12), leaving open the possibility that these or other candidate materials may enable programmable nonlinear waveguides with nonlinearity comparable to the commonly used native-$\chi^{(2)}$ materials and a transparency window stretching into the ultraviolet.

Another limitation is the low update rate of the programmable nonlinearity, which is currently limited to approximately 20 Hz because of the RC time constant of the prototype device (see Sec. S3). The update rate could be improved by using a material for the photoconductor layer that has higher photoconductivity than SRN. The device is also limited by its need for AC operation; because the $\chi^{(2)}$ nonlinearity is also modulated at the AC frequency, the rest of the system—including the optical inputs—needs to be synchronized to this modulation. This limitation could

be eliminated by using a conductive oxide as the cladding, enabling DC operation.

A final limitation is that the device relies on free-space optics for generating and imaging the illumination pattern for programming, which makes the system bulkier than it likely needs to be. The system could be miniaturized by directly integrating a micro-light-emitting-diode (µLED) display on or near the photoconductor layer. As discussed in Ref. [S17], µLED displays can deliver sufficient optical power for this application, making this a promising approach to compact and robust programmable nonlinear photonic systems.

S2. POTENTIAL FUTURE APPLICATIONS OF PROGRAMMABLE NONLINEAR PHOTONICS

In this section, we provide a list of potential applications of programmable on-chip nonlinear photonics, along with quantitative discussions of their possible performances. For the performance analysis of SiN programmable waveguides, we take a conservative approach to the values of nonlinearities, relying only on normalized (slope) conversion efficiencies already demonstrated in our work. This is because optical nonlinearity is an inherent property of a material and cannot be significantly enhanced even by advanced fabrication techniques unless different materials are employed. On the other hand, propagation loss depends strongly on fabrication processes, and we expect there is more room for improvement beyond the values demonstrated in the prototypical devices. Thus, we cite loss numbers reported in the literature for the performance estimations. Advances in fabrication techniques to reduce propagation loss in programmable waveguides would be an essential step to realize some of the applications proposed in this section. Finally, some of the applications require QPM gratings over a meter in length, which is well beyond the centimeter scale demonstrated in this work. Developing a scheme to efficiently optimize the QPM grating structure based on real-time feedback would be essential to realize such operations.

A. Highly programmable on-chip optical pulse shaper

Building an optical pulse shaper that can access both a large programmable time scale T_{window} and temporal resolution ΔT is a challenging task because the device needs to cover physical processes with vastly different timescales. The ratio between these timescales, known as the time-bandwidth product $\mathrm{BT} = \Delta T/T_{\mathrm{window}}$, is a figure of merit that corresponds to the number of programmable parameters of a pulse shaper [S18]. In Table S3, we summarize the performance of notable demonstrations of optical pulse shapers. Here, we show that a programmable channel waveguide could potentially realize a competitive on-chip pulse shaper with as many as 3000 programmable parameters, whose expected performance is shown in the table. Our proposed approach offers sub-fs timing resolution (i.e., terahertz of bandwidth) and hundreds picoseconds of programmable time window (i.e., gigahertz-level frequency resolution), which could be heterogeneously integrated in a larger photonic system as a flexible, on-demand, and on-chip pump source. Beyond applications in photonics, optical pulse shaping in such a frequency range has been used for radio-frequency (RF) and terahertz wave generation. That is, by detecting the synthesized optical pulses with high-bandwidth photodetectors, the photocurrent inherits the temporal-domain intensity profile of the optical pulse [S19, S20]. The accessible bandwidth of the generated electronic signal can go far beyond what is possible through purely electronic means.

Device type	Time window T_{window}	Time resolution ΔT	Programmable parameters (BT)	Reference
**Programmable channel waveguide (theory)**	**300 ps**	**200 fs**	**1500**	**This work (theory)**
Commercial Fourier transform optical pulse shaper	3.2 ps	5 fs [a]	640	Ref. [S21]
Meta-surface Fourier transform optical pulse shaper	6.6 ps	10 fs [b]	660	Ref. [S22]
Commercial acousto-optic programmable dispersive filter	14 ps	5.3 fs [c]	2600	Ref. [S23]
Free-space line-by-line pulse shaper	380 ps [d]	2 ps	190	Ref. [S20]
Integrated line-by-line pulse shaper	40 ps [e]	1.25 ps	32	Ref. [S24]

TABLE S3: Summary of the performance of optical pulse shapers. [a]Calculated from the maximum spectral window of 600 nm around 1000 nm. [b]Calculated from the spectral window between 700 nm and 900 nm. [c]Calculated from the spectral window between 650 nm and 1100 nm. [d]2.6 GHz spectral resolution. [e]25 GHz channel spacing.

A possible integration of a pulse shaper based on a programmable waveguide is shown in Fig. S1. High-power pump pulses are coupled into the SiN programmable channel waveguide. The programmable QPM grating then engineers the shape of the SHG pulse into desired forms [S25–S27]. With programmable illumination implemented using micro-LED arrays [S17] and pump light source provided by micro-combs, the entire pulse shaper setup could be fully integrated on-chip. To maximize the programmability of the device, we consider maximizing its length. The superb properties of SiN allow access to 0.4 dB/m loss [S14], and spiral-shaped waveguides with more than a meter of length have been demonstrated on-chip [S28, S29]. Here, we assume $L_{\text{tot}} = 1.5\,\text{m}$ of spiral-shaped waveguide with dispersion engineering to minimize group-velocity dispersion. While performing periodic poling on such a long and winding structure would be challenging with conventional means, the real-time programmability of our approach allows robust optimization of QPM gratings based on experimental feedback, as demonstrated in our work. Assuming a moderate group-velocity mismatch of $\text{GVM} = -0.2\,\text{ps/mm}$ and using the pulse shaping scheme with QPM grating [S25], the device could engineer arbitrary SH pulse shapes with a temporal window as large as $T_{\text{window}} = L_{\text{tot}} \times |\text{GVM}| = 300\,\text{ps}$. The smallest programmable temporal feature size is set by the duration of the pump pulse, $\Delta T = T_{\text{pump}}$. Assuming $T_{\text{pump}} = 200\,\text{fs}$, the time-bandwidth product (BT) of the pulse shaper (i.e., the number of programmable spectral-temporal features) would become $\text{BT} = T_{\text{window}}/\Delta T = 1500$. These numbers are summarized in Table S3.

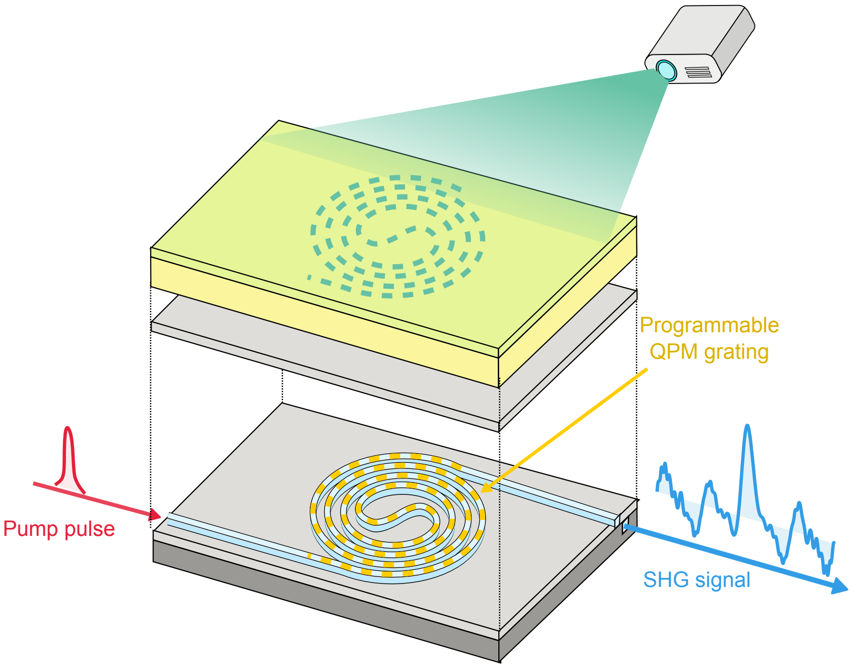

FIG. S1: An illustration for the possible future implementation of on-chip pulse shaper with a long programmable channel waveguide.

Finally, we comment on the efficiency of our proposed pulse shaper. To verify that a channel waveguide can enhance the conversion efficiency, we fabricated a SiN programmable channel waveguide (see Methods). On this waveguide, we observed a normalized SHG conversion efficiency of $\eta_{\text{norm}} = 2 \times 10^{-3}\,\%/\text{W}$, or equivalently a slope conversion efficiency of $\eta_0 = 4 \times 10^{-3}\,\%/\text{W/cm}^2$, showing a 40-fold improvement over the programmable planar waveguide. We assume that the proposed pulse shaper consists of a channel waveguide with the same nonlinearities as demonstrated in our work.

Because there is a finite group-velocity mismatch, we need to account for the effects of temporal walk-off in the calculation of conversion efficiency. More specifically, since the FH and SH pulses temporally walk off every $L_{\text{walk-off}} = T_{\text{pump}}/|\text{GVM}| = 1\,\text{mm}$ of propagation, the peak SH power will approximately be $P_{\text{SH}} = \eta_0 P_{\text{FH}}^2 L_{\text{walk-off}}^2$, where P_{FH} is the peak power of the FH mode. The total energy conversion efficiency of the device, accounting for temporal walk-off (but assuming no pump depletion), is approximately given as

$$\eta_{\text{tot}} = \frac{P_{\text{SH}} T_{\text{window}}}{P_{\text{FH}} T_{\text{pump}}} = \frac{\eta_0 P_{\text{FH}} T_{\text{pump}} L_{\text{tot}}}{\text{GVM}}, \tag{S1}$$

which reaches a conversion efficiency of $\eta_{\mathrm{tot}} = 10\,\%$ with a peak power of $P_{\mathrm{FH}} = 170\,\mathrm{W}$. Ultrashort pulses with such peak powers are straightforward to obtain from fiber-based mode-locked lasers (for instance, the ELMO HP laser we used in our work outputs more than 30 kW of peak power), indicating that the entire programmable arbitrary optical-pulse synthesizer system could be integrated into a compact setup with no free-space optical components. Also, with a relatively large cross-section of $2\,\mu\mathrm{m} \times 4\,\mu\mathrm{m}$, this waveguide can handle 60 kW of peak power when pumped by femtosecond pulses (see Sec. S4 D).

B. Programmable quantum frequency converter

A quantum frequency converter (QFC) is a device that converts the frequency of quantum-optical signals [S30]. One of the major applications of QFCs is quantum networking, where the frequency of photons emitted from quantum nodes—often composed of atoms or vacancy centers—is converted to a frequency more suitable for long-range communication, and vice versa [S31]. For this application, the ability to tune the target wavelengths is an essential feature of a QFC. It can compensate for the inherent inhomogeneities of the transition frequencies of certain quantum emitters and enables wavelength division multiplexing (WDM) to boost communication capacity. Recently, Cisco Quantum Labs published a roadmap for scalable quantum networking with dense WDM [S32], where reconfigurable quantum interfaces—i.e., QFCs with tunable phase-matching conditions—play a central role. However, a suitable device has not yet been demonstrated. Our work could enable such an application, thanks to the fast and extremely wide programmability of the phase-matching conditions. More broadly, Ref. [S33] reviews the unique role that nontrivial QPM structures can play in QFC applications, many of which can be implemented and dynamically switched on our platform. Below, we quantitatively show that a SiN programmable channel waveguide could realize high-efficiency conversion of quantum signals using sum/difference frequency (SFG/DFG) processes.

As illustrated in Fig. S2, we consider the same device design as discussed in Sec. S2 A but operated differently. The device consists of a programmable SiN channel waveguide with a slope SHG conversion efficiency of $\eta_0 = 4 \times 10^{-3}\,\%/\mathrm{W/cm}^2$ (as demonstrated in this work; see Methods) and a total length of 1.5 m. We consider three different modes of light labeled as the "atomic mode", "communication mode", and "pump mode", with frequencies ω_a, ω_c, and ω_p, respectively. The goal of a QFC is to convert photons in the atomic mode to the communication mode via a DFG process $\omega_a - \omega_p = \omega_c$. Note that this process also transduces photons in the communication mode to the atomic mode at the same time. Assigning annihilation operators to the atomic and communication modes as $\hat{a}$ and $\hat{c}$, the DFG process induces the following transformation:

$$\hat{c}(L) = \cos(\sqrt{\eta_{0,\mathrm{DFG}} P_p} L)\hat{c}(0) + \sin(\sqrt{\eta_{0,\mathrm{DFG}} P_p} L)\hat{a}(0), \tag{S2}$$

where $L = 1.5\,\mathrm{m}$ is the length of the waveguide, P_p is the power of the pump field, and $\eta_{0,\mathrm{DFG}}$ is the DFG slope conversion efficiency. Assuming weak wavelength dependence of the nonlinearity, $\eta_{0,\mathrm{DFG}} = 4\eta_0$ holds, with the slope SHG conversion efficiency of the waveguide $\eta_0 = 4 \times 10^{-3}\,\%/\mathrm{W/cm}^2$. Full frequency conversion is achieved when $\hat{c}(L) = \hat{a}(0)$, corresponding to $P_p = 0.7\,\mathrm{W}$. Such a power level can be straightforwardly achieved using quasi-CW pulsed operation. The normalized conversion efficiency of the device is $\eta_{0,\mathrm{DFG}} \times (1.5\,\mathrm{m})^2 = 360\,\%/\mathrm{W}$, which exceeds the value 80 %/W of a commercially available QFC [S34]. For a group-velocity mismatch of $-0.2\,\mathrm{ps/mm}$, the phase-matching bandwidth is expected to be 3.2 GHz. Such an extremely narrow bandwidth, enabled by the long waveguide, can play the role of a filter, rejecting undesired background photons [S35]. Overall, we expect the realization of such a programmable QFC to be feasible on SiN. The conversion efficiency η_0 we assumed was observed in our work. Furthermore, SiN waveguides longer than a meter have been demonstrated in various studies [S28, S29], and optical loss as low as 0.4 dB/m has been achieved [S14]. An ability to reconfigure the QPM grating based on real-time experimental feedback would facilitate efficient quasi-phase matching on such a long waveguide.

C. Widely tunable and highly efficient integrated light sources

Nonlinear $\chi^{(2)}$ nanophotonic resonators hold the key to highly efficient integrated light sources. For second-harmonic generation, the highest normalized conversion efficiency to date has reached $5 \times 10^6\,\%/\mathrm{W}$, which was demonstrated in thin-film lithium niobate [S36]. The performance of other nonlinear optical processes, such as optical parametric oscillation [S37], can also be significantly improved by high-Q resonators. A crucial drawback of such integrated photonics is the lack of tunability. That is, the phase-matching condition is typically fixed during device fabrication, making each device only useful for a narrow bandwidth. Recently, all-optical poling (AOP), induced by the combination of DC Kerr and photogalvanic effects, has attracted attention as a solution to this challenge [S1]. By leveraging AOP, Ref. [S7] demonstrated efficient green-light generation over a 2.6 THz tunable bandwidth. At the same time,

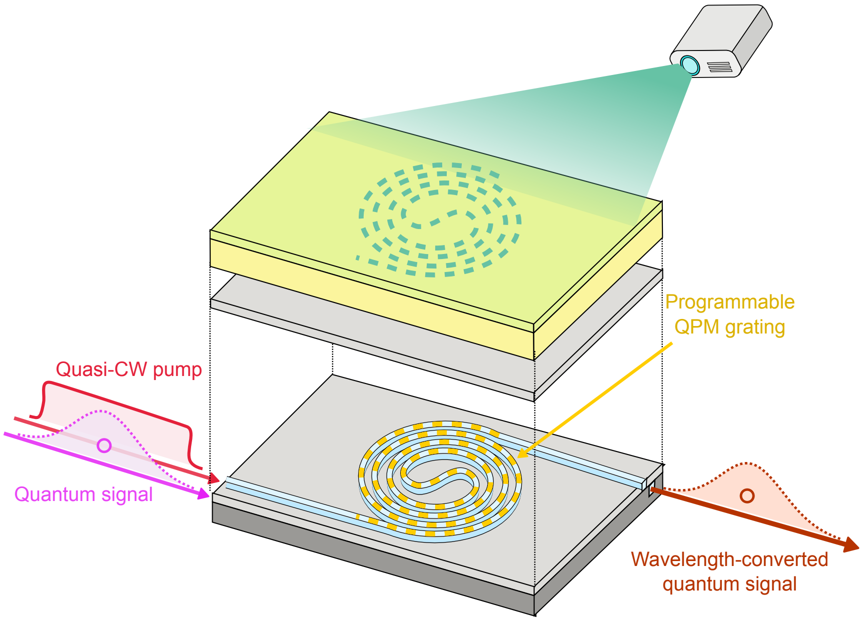

FIG. S2: Illustration of a possible implementation of a QFC with a programmable nonlinear waveguide. The pump light drives a frequency conversion of a weak input quantum signal to the communication mode.

the AOP-based approach still faces several challenges. First, AOP involves a complicated buildup of interference patterns, which takes several seconds to reconfigure [S7]. Second, the formation of AOP requires efficient SHG, but one may be interested in other nonlinear-optical processes—e.g., spontaneous parametric downconversion (SPDC). In Ref. [S5], the authors first drove the resonator with FH light to produce AOP, then switched the pump wavelength to SH to realize SPDC. Furthermore, they observed that the effective $\chi^{(2)}$ nonlinearity of the AOP faded over a time scale of 40 s $\sim$ 70 s during the SPDC operation. The rate of the decay increased with stronger pump fields, and they hypothesized that the effect was caused by multiphoton excitation from the pump. This complicates efforts to increase pump power to achieve stronger gain—for example, in the generation of squeezed states or for realization of optical paramatric oscillators.

Our approach based on programmable $\chi^{(2)}$ nonlinearity can provide a resolution to these challenges. While benefiting from the superb optical properties of the SiN photonics platform, we could induce arbitrary QPM grating patterns with an update speed potentially approaching 200 Hz (see Sec. S1). Furthermore, because the QPM grating is produced by an external bias electric field, there is no issue with the QPM grating fading even when no SHG is taking place, making it attractive for applications like SPDC. Below, we show that a microring resonator made of a SiN programmable nonlinear waveguide can indeed achieve competitive performance for various nonlinear optical processes, enabling widely tunable and highly efficient integrated light sources.

In Fig. S3, we depict how such a device may be integrated. For concreteness, we consider a microring resonator with $R = 100\,\mu$m radius, composed of a curved channel waveguide structure. Though the loss of the programmable planar waveguide was rather high (> 1 dB/cm), we verified in Sec. S4 C 4 that high-temperature annealing of the film allows us to reduce the loss further to 0.4 dB/cm. This corresponds to a field decay rate of $\kappa_{\rm FH} = 2\pi \times 109$ MHz, assuming a group index of 2. The intrinsic Q-factor of the resonator for the FH is thus $Q_{\rm FH,0} = \frac{\omega_{\rm FH}}{2\kappa_{\rm FH}} = 8.9 \times 10^5$. Assuming the same Q-factor values for the SH and critical coupling for both modes, the loaded Q-factors of the resonator are expected to be $Q_{\rm FH} = Q_{\rm SH} = 4.4 \times 10^5$. The normalized SHG conversion efficiency of the device takes the form

$$\eta_{\rm norm} = \frac{8 g^2 Q_{\rm FH}^2 Q_{\rm SH}}{\hbar \omega_{\rm FH}^4}, \tag{S3}$$

where g denotes the nonlinear coupling constant [S36]. The value of g can be calculated via

$$g = \sqrt{\frac{\hbar \omega_{\rm FH} \eta_0 v_g^3}{4\pi R}}, \tag{S4}$$

where η_0 is the normalized slope conversion efficiency of the waveguide [S38]. For the programmable channel waveguide, we demonstrated $\eta_0 = 4 \times 10^{-3}\,\%/\mathrm{W/cm^2}$, leading to $g = 2\pi \times 1.9\,\mathrm{kHz}$. Plugging in these numbers, we find $\eta_{\mathrm{norm}} = 42\,\%/\mathrm{W}$ using only values demonstrated in this work. It is worth noting that SiN is known for its superb ability to achieve extremely low-loss photonic devices. SiN microring resonators with intrinsic Q-factors exceeding 60 million have been reported [S39]. Considering the steep scaling of $\eta_{\mathrm{norm}} \sim Q^3$, this suggests the potential to reach $\eta_{\mathrm{norm}} = 1.3 \times 10^7\,\%/\mathrm{W}$ with loaded Q-factors of 30 million. Notably, this value exceeds the highest recorded SHG conversion efficiency of $5 \times 10^6\,\%/\mathrm{W}$ on integrated photonics, which was demonstrated on a thin-film lithium niobate microring resonator [S36].

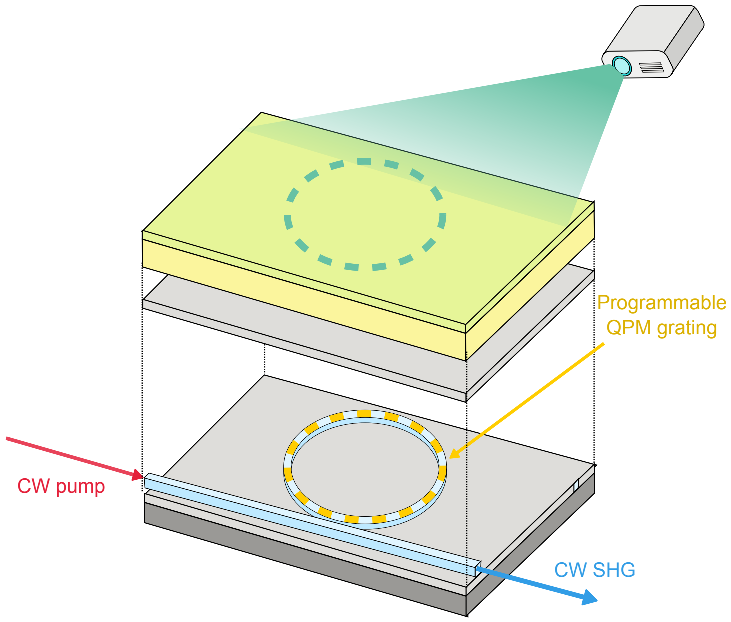

FIG. S3: An illustration of a possible future implementation of a programmable nonlinear microring resonator for a tunable SHG.

Another important application of $\chi^{(2)}$ nonlinear photonics is the optical parametric oscillator (OPO), where short-wavelength pump light parametrically downconverts to produce signal light at a longer wavelength. An important figure of merit for an OPO is the threshold power P_{th}, above which macroscopic signal light is generated. The lowest OPO threshold reported to date on an integrated platform is $P_{\mathrm{th}} = 30\,\mu\mathrm{W}$, demonstrated in Ref. [S37] on a thin-film lithium niobate microring resonator. Assuming critical coupling, the OPO threshold takes the form

$$P_{\mathrm{th}} = \frac{\hbar \omega_{\mathrm{FH}}^4}{8 g^2 Q_{\mathrm{FH}}^2 Q_{\mathrm{SH}}}. \tag{S5}$$

Assuming a loaded Q-factor of 30 million, we expect $P_{\mathrm{th}} = 7.7\,\mu\mathrm{W}$ on a programmable SiN OPO.

Overall, we can see that a SiN programmable microring resonator has the potential to achieve state-of-the-art performance, even compared to more established, non-programmable platforms with much stronger native $\chi^{(2)}$ non-linearities (e.g., lithium niobate with $\chi^{(2)} = 50\,\mathrm{pm/V}$). This is due to the exceptionally low loss of SiN, which can fully compensate for the lower value of $\chi^{(2)}$ optical nonlinearity.

D. Programmable quantum light sources

Phase matching plays a crucial role in the application of biphoton generation using spontaneous parametric down-conversion (SPDC). It determines various properties of the generated photon pair, such as wavelength, spatial profiles, and polarization [S40]. Furthermore, nontrivial QPM grating structures can engineer more complex correlation structures of photons, enabling separable [S41], highly multimodal [S42], and even grid-like [S43] correlations. Reference [S33] provides a broader review of the potential of QPM engineering for SPDC light sources. The ability to

programmably switch among these functions would enable highly flexible implementations of quantum technologies. For instance, similar to the concept proposed in Ref. [S32], tunability of the wavelength of entangled photons would allow wavelength-division multiplexing in quantum networking, boosting the communication rate. There have been various demonstrations of tunable SPDC—e.g., via temperature tuning [S44], angle tuning [S45], or ferroelectric liquid crystals [S46]—but engineering the full QPM grating structure remains elusive. To this end, a programmable nonlinear waveguide offers an ideal solution to these challenges. Below, we provide quantitative discussions on the potential performance of SPDC sources based on our approach.

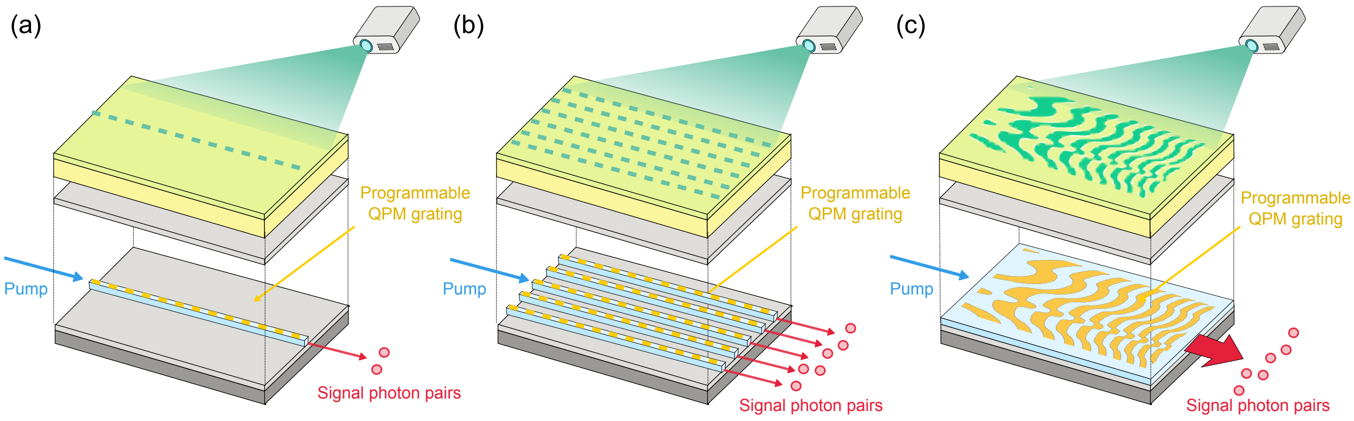

FIG. S4: Illustration of possible future implementations of programmable SPDC on (a) a channel waveguide, (b) a waveguide array, and (c) a planar waveguide.

A figure of merit often employed for quantitative characterizations of SPDC is the normalized brightness B_{norm}, which quantifies the number of photons generated per second per mW of pump power. For instance, $B_{\text{norm}} = 6.4 \times 10^4$ pairs/s/mW was measured for bulk beta barium borate (BBO) crystals, which are widely used in quantum optics experiments [S47]. On a thin-film lithium niobate waveguide, $B_{\text{norm}} = 2.3 \times 10^{11}$ pairs/s/mW was demonstrated [S48]. Note that for many applications, a very high absolute count rate is neither necessary nor desirable, due to the undesired multi-photon events and the limitations in the speed of photon detectors and electronics. Thus, an important consideration is whether one can achieve the desired brightness with a reasonable pump power. Commercial SPDC sources operate around 1×10^4 pairs/s $\sim 1 \times 10^7$ pairs/s [S49–S51], implying the typical range of brightness required for applications.

For the estimation of realistic B_{norm} on programmable waveguides, we first consider a SiN programmable channel waveguide with $L = 0.7$ mm in length and an induced $\chi^{(2)} = 0.47$ pm/V—performance metrics we demonstrated in this work. The normalized brightness of a Type-0 degenerate SPDC process pumped by CW light in a waveguide is estimated as [S52]

$$B_{\text{norm}} = \sqrt{\frac{2}{\pi^3}} \frac{2}{3\epsilon_0 c^3} \frac{n_{g,a}^2}{n_a^4 n_b} \frac{d_{\text{eff}}^2 \omega_b^2}{\sqrt{|\beta_2|}} \left| \frac{\sigma_b^2}{\sigma_a^2 + 2\sigma_b^2} \right|^2 \frac{1}{\sigma_b^2} L^{3/2}, \tag{S6}$$

where β_2 is the group-velocity dispersion at the FH wavelength, L is the length of the waveguide, and ω_b is the angular frequency of the pump (i.e., SH) light with wavelength 780 nm. For a programmable nonlinear waveguide, we have the effective nonlinearity $d_{\text{eff}} = \frac{1}{2\pi}\chi^{(2)}$. Here, the labels "a" and "b" denote the FH and SH modes, respectively. The formula assumes that both FH and SH have radially symmetric Gaussian beam profiles with beam radii of σ_a and σ_b, respectively. For the estimation of the SPDC rate, we assume the refractive indices of the FH and SH modes as $n_a = n_b = 2$, and the group index as $n_{g,a} = 2$. Assuming a moderate group-velocity dispersion of $|\beta_2| = 50\,\text{fs}^2/\text{mm}$, beam radii of $\sigma_a = \sigma_b = 1\,\mu\text{m}$, and a total length of $L = 7$ mm, we find the normalized brightness of the device to be $B_{\text{norm}} = 8.4 \times 10^5$ pairs/s/mW—an order of magnitude greater than that of bulk BBO crystals. An absolute brightness of 1×10^7 pairs/s can be achieved with a reasonable pump power of 12 mW.

So far, we have focused our discussion on a programmable channel waveguide, but geometries with more spatial degrees of freedom offer intriguing potential for spatially multiplexed quantum light sources. In Fig. S4, we show illustrations of such extensions. For instance, various works have theoretically shown that SPDC occurring in an array of 1D waveguides can be used to produce a variety of spatial correlations of photons, such as cluster states, where pump shaping and engineered QPM gratings play central roles in determining the generated states [S53–S55]. Programmable SPDC on planar waveguide geometries could offer unique advantages as well. For example, it enables

non-collinear generation of biphotons, as leveraged in Ref.[S56], providing a simple way to separate entangled photon pairs. Additionally, free-form propagation on a planar waveguide could offer comparable or even higher channel capacity and beam maneuverability [S17]. A programmable waveguide can realize two-dimensional controllability of QPM gratings on such geometries, offering a unique opportunity for programmable quantum light sources with spatio-spectral controls of entanglement structures.

E. 100 %-yield QPM gratings

A key benefit of realizing functions on programmable photonic devices—whether in planar-waveguide geometry or other configurations—is that inevitable material and fabrication imperfections when a device is made, as well as environmental fluctuations when it is operated, can be compensated for. As a result, high performance can be achieved with better fabrication yield or less sophisticated fabrication than conventional devices while maintaining robustness to material imperfections and variable operating conditions.

For example, nanoscale thickness variations in thin-film lithium niobate (TFLN) waveguides typically place an effective limit on the useful length of a periodically poled waveguide and the maximum achievable conversion efficiency. In Ref. [S57], the authors reported how they could circumvent this limitation by precisely measuring the thickness distribution and adapting the poling to compensate for the thickness inhomogeneity. Programmable nonlinear photonic devices offer a fundamentally different solution to such challenges. By dynamically optimizing the QPM grating structure for an experimentally measured figure of merit (FOM), programmable devices can be adapted in real time to maximize performance. In the main text, we demonstrated both robustness to fluctuations in pump wavelength, where the FOM was conversion efficiency, and in situ inverse design, where the FOM was the similarity between the measured and target SHG spectra. We note that the prototype devices demonstrated in in this work had a much thicker film and lower field confinement than the aforementioned TFLN waveguides, making them less sensitive to poling errors. Thus, such real-time adaptive poling was not technically necessary to achieve efficient phase-matching conditions over lengths of less than a centimeter. Rather, these demonstrations should be viewed as proofs of concept for futuristic devices with more stringent requirements for the accuracy of periodic poling, e.g., meters-long programmable waveguides as discussed in Sec. S2 A and Sec. S2 B. Furthermore, there are many situations in nonlinear optics—supercontinuum generation being a prominent example [S58]—where device behavior is extremely sensitive not only to the device parameters but also to the field profile of the pump light, making it challenging to achieve exact agreement between simulation and experiment. The inverse-design experiments reported in the main text were performed without prior characterization of the pump; in situ inverse design using programmable devices may ultimately enable the realization of quantitatively correct behavior even for complex nonlinear-optical processes that we don't have accurate simulation models for.

S3. ELECTRICAL PROPERTIES OF PROGRAMMABLE NONLINEAR WAVEGUIDES

This section introduces an electric circuit model for a programmable waveguide, demonstrating how photoconductivity can be used to control the electric-field-induced $\chi^{(2)}$. We also discuss the necessary considerations for optimal device operation, the potential for improving the induced nonlinearity, and the resolution limits of programmable $\chi^{(2)}$ nonlinearity.

A. Lumped-element circuit model

The electrical properties of a programmable waveguide can be approximated by modeling each layer of the device as a lumped-element circuit element with a defined impedance [S17]. For the cladding and core layers, we assume that their conductivities are negligible. The impedance of each layer at the frequency $\omega = 2\pi f$ is given by

$$Z_\alpha = \frac{1}{i\omega C_\alpha}, \tag{S7}$$

where

$$C_\alpha = \frac{\epsilon_0 \epsilon_\alpha A}{d_\alpha} \tag{S8}$$

is the capacitance of the layer. Here, the subscript $\alpha \in \{\text{cladding}, \text{core}\}$ denotes the layer, ϵ_0 is the vacuum permittivity, ϵ_α is the relative permittivity, d_α is the layer thickness, and A is the area. In this context, the term "cladding" refers to the combined stack of the top and bottom cladding layers.

The photoconductor layer, however, can exhibit nonnegligible conductance even in the absence of illumination. Therefore, we account for both its capacitive and conductive contributions to the impedance as

$$Z_{\mathrm{B/D}} = \frac{1}{i\omega C_{\mathrm{PC}} + 1/R_{\mathrm{B/D}}}, \tag{S9}$$

where

$$C_{\mathrm{PC}} = \frac{\epsilon_0 \epsilon_{\mathrm{PC}} A}{d_{\mathrm{PC}}} \tag{S10}$$

is the capacitance of the photoconductor layer. The subscripts "B" and "D" denote the bright and dark states, respectively. The resistance $R_{\mathrm{B/D}}$ is given by

$$R_{\mathrm{B/D}} = \frac{d_{\mathrm{PC}}}{\sigma_{\mathrm{B/D}} A}, \tag{S11}$$

where σ_{B} and σ_{D} are the conductivities of the photoconductor in the bright and dark states, respectively.

When a total voltage V_{tot} is applied, the overall stack acts as a voltage divider. Specifically, the voltage across the core layer in a bright or dark state is

$$V_{\mathrm{B/D}} = \frac{Z_{\mathrm{core}}}{Z_{\mathrm{core}} + Z_{\mathrm{cladding}} + Z_{\mathrm{B/D}}} V_{\mathrm{tot}}. \tag{S12}$$

To illustrate how photoconductivity enables control of the electric field within the core, we consider an idealized limit in which the photoconductor layer is infinitely thick ($d_{\mathrm{PC}} \to \infty$) and the bright-state photoconductivity is infinitely high (i.e., $d_{\mathrm{PC}}/\sigma_{\mathrm{B}} \to 0$). In this limit, we have $Z_{\mathrm{B}} \to 0$ and $Z_{\mathrm{D}} \to \infty$, leading to

$$V_{\mathrm{B}} = \frac{Z_{\mathrm{core}}}{Z_{\mathrm{core}} + Z_{\mathrm{cladding}}} V_{\mathrm{tot}} \quad \text{and} \quad V_{\mathrm{D}} = 0. \tag{S13}$$

Thus, photoconductivity enables control of the bias electric field over a dynamic range from

$$V_{\mathrm{max}} = \frac{Z_{\mathrm{core}}}{Z_{\mathrm{core}} + Z_{\mathrm{cladding}}} V_{\mathrm{tot}} \tag{S14}$$

down to 0. In a realistic device with a finite photoconductor thickness and finite bright-state conductivity, the dynamic range is reduced.

In nonlinear optics, the contrast between the bright and dark states, $V_{\mathrm{B}} - V_{\mathrm{D}}$, produces a QPM grating. Since the efficiency of SHG is proportional to the square of the contrast in $\chi^{(2)}$ nonlinearity, the SHG power P_{SH} can be expressed as

$$P_{\mathrm{SH}} \propto \Delta V^2 = |V_{\mathrm{B}} - V_{\mathrm{D}}|^2 = \left| \frac{Z_{\mathrm{core}}}{Z_{\mathrm{core}} + Z_{\mathrm{cladding}} + Z_{\mathrm{B}}} - \frac{Z_{\mathrm{core}}}{Z_{\mathrm{core}} + Z_{\mathrm{cladding}} + Z_{\mathrm{D}}} \right|^2 V_{\mathrm{tot}}^2. \tag{S15}$$

In Fig. S5(a), we present a fit of the model in Eq. (S15) to our experimental data. In this measurement, we varied both the frequency ω and the amplitude V_{tot} of the bias field, and we recorded the generated SHG power via a spectrometer. The fit showed good agreement between the model and the experiment, indicating that the optimal operating frequency was $f = \omega/2\pi = 5\,\mathrm{Hz}$. For all the experiments reported in this paper, we applied a bias voltage $V_{\mathrm{tot}} = 1000\,\mathrm{V}$ to our device with frequency 5 Hz, unless otherwise specified. From the figure, we found the frequency at which the induced $\chi^{(2)}$ nonlinearity became 80% of the optimal value (i.e., where the SHG conversion efficiency dropped to $(80\%)^2 = 60\%$) was approximately 20 Hz. The fit also yielded the conductivities $\sigma_{\mathrm{B/D}}$ at various V_{tot} values, as shown in Fig. S5(b).

On the basis of these values, we estimate the potential improvement in nonlinearity achievable with further optimization of the photoconductor. For this analysis, we use the nominal values $\sigma_{\mathrm{B}} = 1.8 \times 10^{-8}\,\Omega^{-1}\mathrm{m}^{-1}$ and $\sigma_{\mathrm{D}} = 1.6 \times 10^{-9}\,\Omega^{-1}\mathrm{m}^{-1}$ obtained from the fit at $V_{\mathrm{tot}} = 600\,\mathrm{V}$, along with the optimal operating condition $\omega/2\pi = 5\,\mathrm{Hz}$, to determine the present voltage contrast $\Delta V_{\mathrm{present}}$ that was achievable with our current waveguide design.

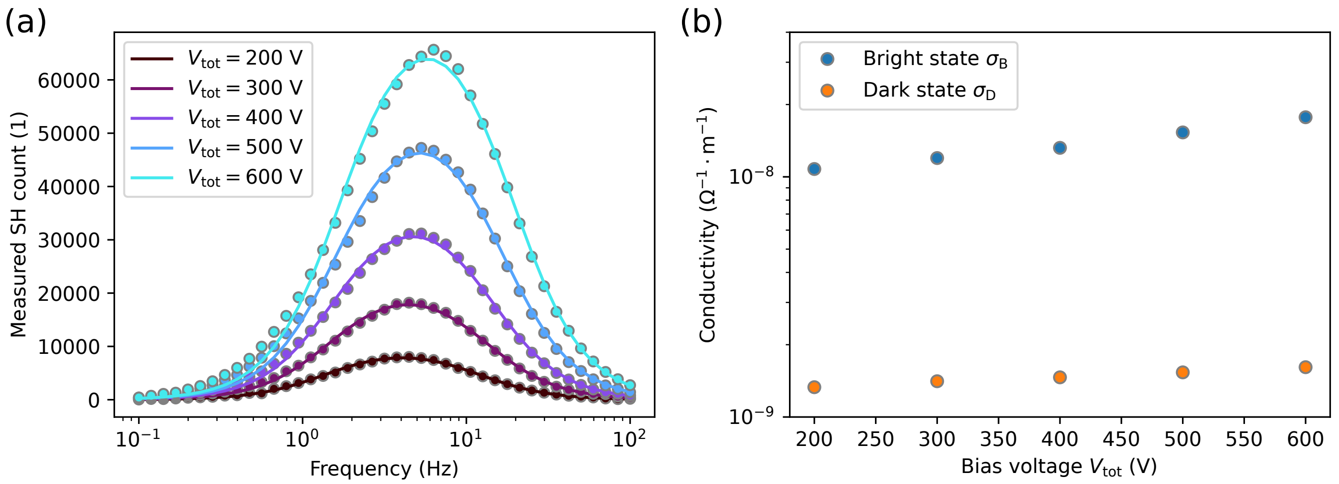

FIG. S5: (a) Circles: Experimentally measured SHG power for various bias voltages V_{tot} and its frequency. Solid lines: Theoretical fit based on the model Eq. (S15). We assume that $\epsilon_{\text{cladding}} = 3.9$ for the silicon dioxide cladding, $\epsilon_{\text{core}} = 6.0$ for the silicon nitride core, and $\epsilon_{\text{PC}} = 8.0$ for the photoconductor composed of SRN. The thicknesses of the layers are $d_{\text{cladding}} = 2\,\mu\text{m}$, $d_{\text{core}} = 2.05\,\mu\text{m}$, and $d_{\text{PC}} = 7.5\,\mu\text{m}$. See the Methods section for the details of the device fabrication. We note that the dependence on A is canceled in Eq. (S15). (b) Numerically determined bright- and dark-state conductivities of the photoconductor for various total bias voltages V_{tot}.

The theoretical upper bound on the voltage contrast, denoted as ΔV_{max}, is reached when the photoconductor exhibits perfect switching characteristics and is infinitely thick. Under these ideal conditions, $\Delta V_{\text{max}} = V_{\text{max}}$. The ratio

$$\mathcal{R}_{\text{max}} = \frac{\Delta V_{\text{max}}}{\Delta V_{\text{present}}} \tag{S16}$$

quantifies the potential for improvement. Numerically, we find $\mathcal{R}_{\text{max}} \approx 2.3$, which indicates that the induced $\chi^{(2)}$ could be larger by this factor than that measured in our experiment.

B. Resolution limit imposed by electric field fringing

Notably, the theoretical upper limit assumes an infinitely thick photoconductor, a condition that would lead to significant fringing of the electric field and reduce the resolution. In practice, the use of a reasonably thin photoconductor is preferable to avoid such issues. Under the assumption of perfect switching for a photoconductor of finite thickness (i.e., $\sigma_B \to \infty$ and $\sigma_D = 0$), our estimates indicate that the induced $\chi^{(2)}$ nonlinearity could be increased by a factor of approximately 1.2.

Thus far, we have assumed uniform programming illumination on a programmable nonlinear waveguide to model its electric properties. In reality, however, the programming illumination can exhibit small spatial variations, which may partially invalidate this assumption. In the following, we study how these spatial variations affect the electric field distribution inside the core by analyzing the resolution limit imposed by electric field "fringing".

For this analysis, we consider a simplified model of a programmable nonlinear waveguide, as shown in Fig. S6(a). In this model, when programming illumination with a width of w is projected onto the photoconductor layer, a vertical pillar of fully conductive material of the same width is created. When a bias voltage $V = V_{\text{tot}}$ is applied to the top electrode, the electric potential of this conductive region is fixed at V_{tot}, while the bottom substrate is grounded (i.e., $V = 0$). The electric potential distribution between these boundaries is given by the self-consistent solution of the Poisson equation

$$\nabla \cdot (\epsilon \nabla V) = 0, \tag{S17}$$

where ϵ denotes the electric permittivity distribution of the medium. This equation can be solved for the specified boundary conditions via the finite-difference method [S59] and the biconjugate gradient stabilized algorithm. The

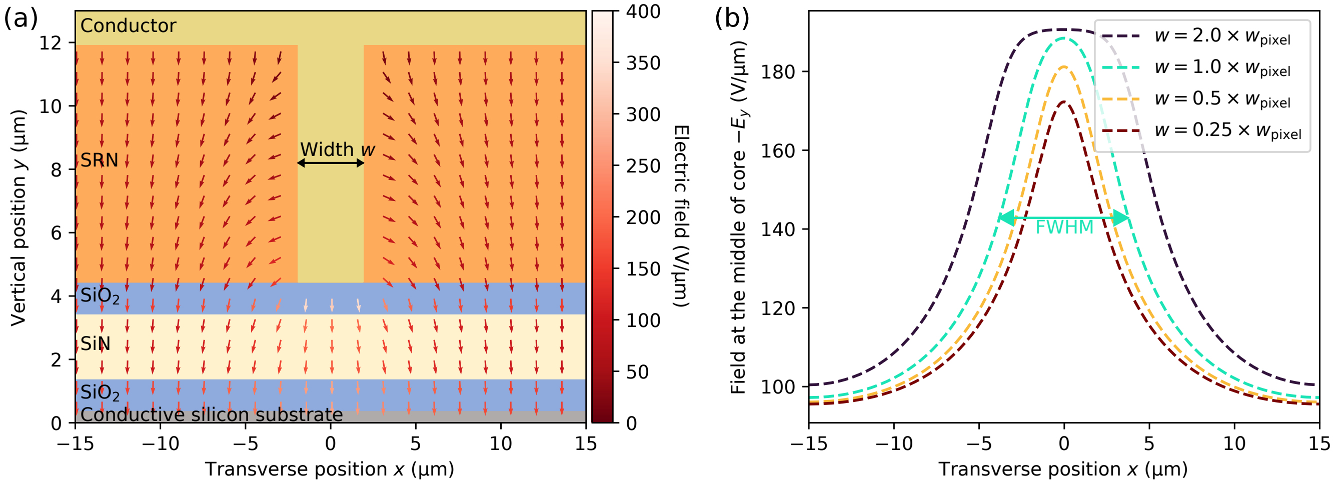

FIG. S6: (a) Numerically simulated distribution of the electric field inside a programmable nonlinear waveguide. The simulation is performed by solving Eq. (S17) via the finite-difference method with the same parameters as in Fig. S5. We assume that $V_{\mathrm{tot}} = 1000\,\mathrm{V}$ and that there is a periodic boundary condition in the transverse dimension. (b) Vertical component of the electric field in the middle of the core layer for various widths of the conductive region w.

resulting electric field distribution, $\mathbf{E} = -\nabla V$, is shown in Fig. S6(a), revealing that the electric fields fringe inside the medium, which blurs the features.

To quantitatively assess the resolution limit imposed by these fringing effects, Fig. S6(b) displays the distributions of the vertical electric field inside the core for various feature sizes w. In our experimental setup, the smallest feature size we can generate with the programming illumination is $w_{\mathrm{pixel}} = 3.772\,\mu\mathrm{m}$, corresponding to a pixel of the SLM (see Sec. S7). According to our simulations, a feature with a width of w_{pixel} produces a bias field distribution with a full width at half maximum (FWHM) of $w_{\mathrm{FWHM}} = 7.5\,\mu\mathrm{m}$, which defines the smallest feature size possible in our experiment. Notably, owing to the fringing effects, an illumination spot smaller than w_{pixel} does not necessarily produce a finer electric field distribution within the core.

C. Photoconductive core for smaller feature sizes

In Sec. S3 B, it is shown that the minimum feature size for programmable nonlinearity $w_{\mathrm{FWHM}} = 7.5\,\mu\mathrm{m}$ is not imposed by the resolution of the programming illumination. Indeed, w_{FWHM} is significantly larger than the fundamental diffraction limit at the wavelength of the programming light 532 nm. Rather, the fringing effects of the electric field inside the photoconductive layer and the top cladding play major roles in blurring the field contrast inside the core layer.

In Fig. S7, we show a future device design that could address this challenge to achieve much smaller feature sizes. In this design, we employ a photoconductive material as the core material. An optical waveguide is formed by the photoconductive core layer and cladding layers, and a transparent electrode is deposited on the top cladding. There is no separate photoconductive layer in this design.

When the programming illumination is applied to the core layer, it locally increases the conductivity. This has the effect of reducing the electric field within this region, decreasing the induced $\chi^{(2)}$ nonlinearity. In contrast, with no programming illumination, the core layer remains highly insulating and experiences a high bias field, thus exhibiting high $\chi^{(2)}$ nonlinearity. In summary, we can dynamically program the distribution of $\chi^{(2)}$ nonlinearity by locally reducing the nonlinearity with illumination, which is the opposite of the design demonstrated in this work. Because the variation in conductivity directly occurs inside the core layer, the aforementioned issue of electric field fringing will be limited to roughly the thickness of the core layer.

A possible drawback of this approach is increased optical loss. As the programming illumination excites free carriers inside the core, it would induce free-carrier absorption (FCA) in a manner that depends on the illumination pattern. It is possible that such FCA imposes a limit on how low the loss can be with this approach. Although this approach is still preliminary and requires further research, we discuss several factors that we believe are notable. First, we note that the level of photoconductivity required for programmable photonics is quite low. As shown in Fig. S5,

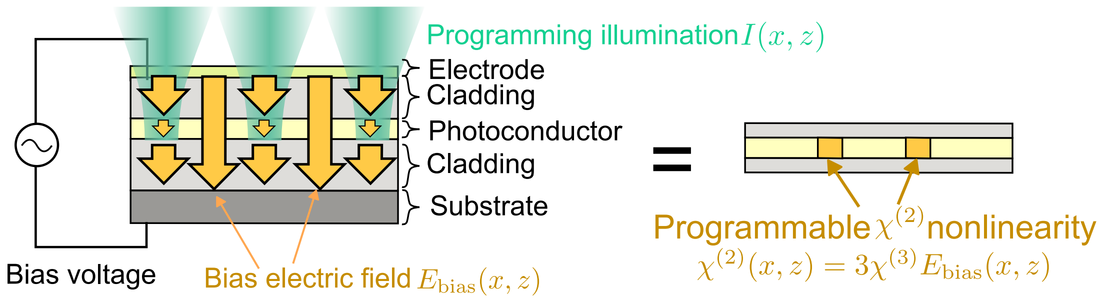

FIG. S7: Conceptual illustration of a programmable nonlinear waveguide using a photoconductive core. When the programming illumination is applied to the photoconductive core, the material becomes conductive, locally reducing the bias electric field. Consequently, we can program the distribution of electric-field-induced $\chi^{(2)}$ nonlinearity by reducing its value through illumination with light.

only a 10× contrast between the bright and dark states is needed to achieve sufficient programmability. This relaxed requirement opens up the possibility of employing materials with lower carrier concentrations, extending beyond the conventional catalog of photoconductive materials. Indeed, SRN, which we used in our work, is typically not employed in conventional photoconductor applications due to its low photoconductivity. Second, as seen in indium tin oxide, the presence of free carriers does not necessarily preclude optical transparency—this is made possible by its large bandgap and high plasma frequency. This underscores the importance of co-engineering optical and electrical properties for the development of an ideal core material.

S4. OPTICAL PROPERTIES OF A PROGRAMMABLE NONLINEAR WAVEGUIDE

This section describes the optical properties of a programmable nonlinear waveguide, including the effective index of the guided mode, the phase-matching condition for SHG, and the optical loss.

A. Waveguide mode

We characterize the guided mode of the programmable waveguide by approximating the cladding as infinitely thick and determining the guided modes of the core layer. Deviations from this approximation result in radiation loss and coupling to the photoconductor layer, as discussed in Sec. S4 C. The refractive indices of the cladding and core layers are shown in Fig. S8 as functions of wavelength.

In this work, we consider the fundamental transverse magnetic (TM) modes of the waveguide for nonlinear optics. For a TM mode, only the H_x, E_y, and E_z field components are nonzero. The spatial profile of a TM mode is obtained as a solution to the eigenvalue equation

$$\beta^2 H_x(y) = \left(n^2(y) \frac{\partial}{\partial y} \frac{1}{n^2(y)} \frac{\partial}{\partial y} + k_0^2 n^2(y) \right) H_x(y), \tag{S18}$$

where β is the effective propagation constant of the mode, $n(y)$ is the refractive index distribution in the vertical direction, and k_0 is the wavenumber in a vacuum. The other nonzero field components are related to H_x by

$$E_y = -\frac{\beta}{\epsilon_0 n^2(y)\omega} H_x, \tag{S19}$$

$$E_z = -\frac{i}{\epsilon_0 n^2(y)\omega} \frac{\partial H_x}{\partial y} \tag{S20}$$

with the angular frequency of light ω.

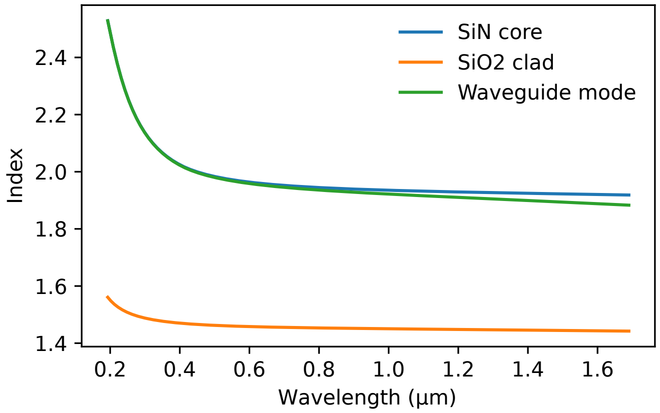

FIG. S8: Optical indices of the SiN core (blue lines), SiO_2 cladding (orange lines), and fundamental TM mode of the optical waveguide (green lines), shown for various wavelengths of light. The index of the SiN core was measured via an ellipsometer, and we use the formula provided in Ref. [S60] for the SiO_2 cladding.

Our planar waveguide is characterized by $n(y) = n_{\mathrm{core}}$ for $|y| \leq d_{\mathrm{core}}/2$ and $n(y) = n_{\mathrm{cladding}}$ elsewhere. In this case, an analytic solution for the fundamental TM mode is given by

$$H_x(y) = \begin{cases} C\cos(k_{\mathrm{core}} y) & \text{for } |y| \leq d_{\mathrm{core}}/2 \\ C\cos(k_{\mathrm{core}} d_{\mathrm{core}}/2)\exp(-\kappa_{\mathrm{core}}(y - d_{\mathrm{core}}/2)) & \text{for } y > d_{\mathrm{core}}/2 \\ C\cos(k_{\mathrm{core}} d_{\mathrm{core}}/2)\exp(-\kappa_{\mathrm{core}}(-y - d_{\mathrm{core}}/2)) & \text{for } y < -d_{\mathrm{core}}/2 \end{cases} \tag{S21}$$

where C is a normalization constant. The value of k_{core} is determined as the smallest solution of the equation

$$k_{\mathrm{core}} \tan\left(\frac{k_{\mathrm{core}} d_{\mathrm{core}}}{2}\right) = \frac{n_{\mathrm{core}}^2}{n_{\mathrm{cladding}}^2}\sqrt{k_0^2\left(n_{\mathrm{core}}^2 - n_{\mathrm{cladding}}^2\right) - k_{\mathrm{core}}^2}. \tag{S22}$$

Equation (S22) is related to the propagation constants according to

$$-k_{\mathrm{core}}^2 + n_{\mathrm{core}}^2 k_0^2 = \kappa^2 + n_{\mathrm{cladding}}^2 k_0^2 = \beta^2. \tag{S23}$$

Importantly, the effective index of the fundamental TM mode is defined as

$$n_{\mathrm{eff}} = \frac{\beta}{k_0}. \tag{S24}$$

Figure S8 shows n_{eff} for our waveguide structure.

B. Phase-matching conditions

For the second-harmonic generation (SHG) of pump light with wavelength λ_0, the phase mismatch is defined as

$$\Delta k = k_2 - 2k_1, \tag{S25}$$

where k_1 and k_2 are the wavenumbers of the fundamental and second-harmonic waves, respectively. Since we are considering SHG between the fundamental TM modes of the waveguide, we have

$$k_1 = \frac{2\pi n_{\mathrm{eff}}(\lambda_0)}{\lambda_0}, \tag{S26}$$

$$k_2 = \frac{4\pi n_{\mathrm{eff}}(\lambda_0/2)}{\lambda_0}. \tag{S27}$$

Quasi-phase matching (QPM) is achieved when the $\chi^{(2)}$ nonlinearity is modulated in a grating-like pattern with a spatial period

$$\Lambda = \frac{2\pi}{\Delta k}. \tag{S28}$$

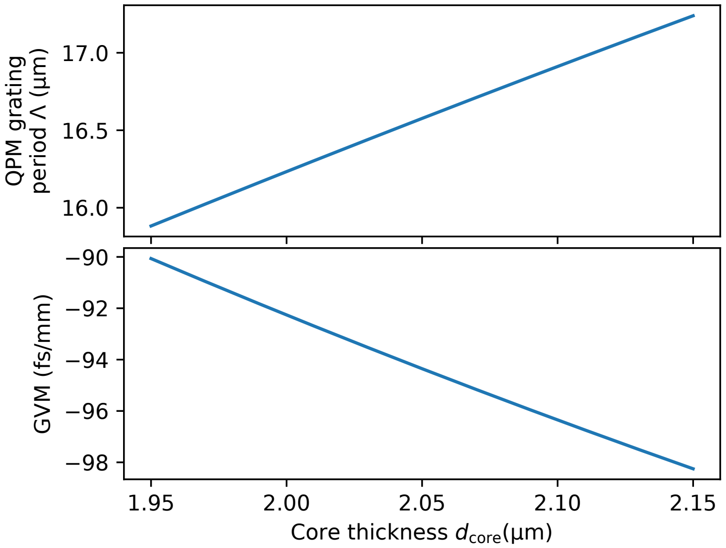

FIG. S9: The numerically calculated QPM grating period and GVM for the SHG of pump wavelength $\lambda_0 = 1.56\,\mu\text{m}$ as functions of the core thickness d_{core}. We use the material index data shown in Fig. S8.

Interestingly, the rate at which the optimal QPM grating period changes with wavelength is proportional to the group velocity mismatch (GVM) between the fundamental and second-harmonic waves:

$$\frac{\partial \Lambda}{\partial \lambda_0} = -\frac{8\pi^2 c}{\Delta k^2 \lambda_0^2}\,\text{GVM}, \tag{S29}$$

where

$$\text{GVM} = \frac{1}{v_{\text{g},1}} - \frac{1}{v_{\text{g},2}} \tag{S30}$$

and where $v_{\text{g},1}$ and $v_{\text{g},2}$ are the group velocities of the fundamental and second-harmonic (SH) waves, respectively [S25].

In Fig. S9, we present the numerically estimated QPM grating period Λ and the GVM at $\lambda_0 = 1.56\,\mu\text{m}$ as functions of the core thickness d_{core}. At the nominal thickness $d_{\text{core}} = 2.05\,\mu\text{m}$, our simulations yield $\Lambda = 16.58\,\mu\text{m}$ and $\text{GVM} = -94\,\text{fs/mm}$, which are in good agreement with the experimentally measured values of $\Lambda = 16.69\,\mu\text{m}$ and $\text{GVM} = -92\,\text{fs/mm}$ (see the main text). As shown in Fig. S9, the nominal film thickness variations of approximately 50 nm can account for these residual discrepancies.

C. Optical loss

Several factors contribute to the optical loss in a programmable nonlinear waveguide. Below, we evaluate the primary contributors in more detail.

1. *Material absorption*

The core material employed for the programmable nonlinear waveguide was PECVD SiN, which can exhibit considerable optical loss at the wavelengths of interest. To characterize the optical loss inside the core, we fabricated several planar waveguides of varying lengths. Laser light was coupled into the fundamental modes of these waveguides, and by comparing the output powers from waveguides of different lengths, we estimated the propagation loss.

Figure S10(a) displays the loss at the fundamental harmonic (FH) wavelength. The peak observed at approximately 1520 nm was attributed to the characteristic absorption of PECVD SiN due to residual hydrogen atoms [S61, S62]. At wavelengths distant from this absorption peak, the loss decreased to approximately 1 dB/cm. Figure S10(b) shows the loss at the second-harmonic (SH) wavelength, where we observed both lower loss and reduced wavelength dependence.

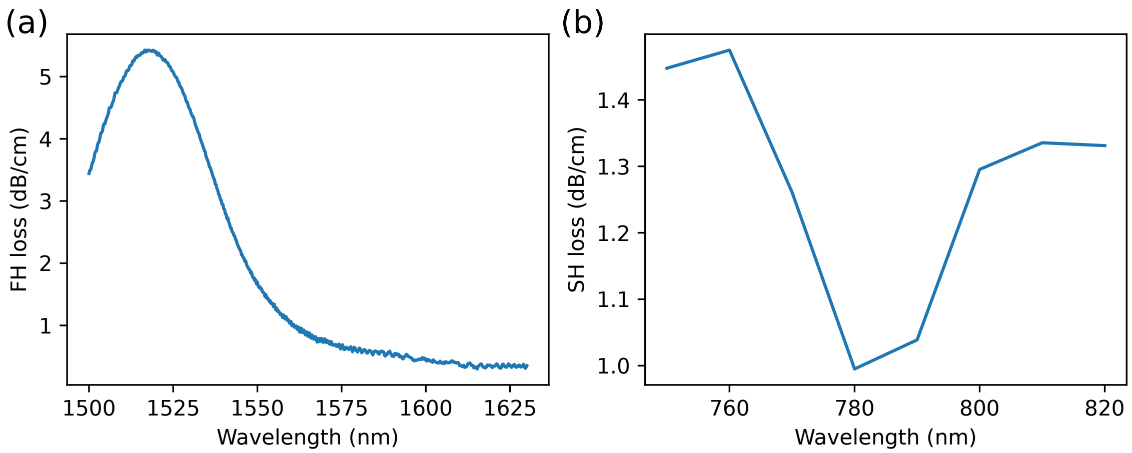

FIG. S10: Optical loss in SiN was measured by comparing transmission through two planar waveguides with lengths of 1.5 cm and 3.0 cm. The waveguides consist of a 1 µm SiO_2 bottom cladding, a 2.05 µm SiN core, and an air top cladding. (a) The loss of FH light, with wavelengths ranging from 1500 to 1630 nm, was measured via a tunable CW laser (TSL-570; Santec) as the light source. (b) The loss of SH light, with wavelengths between 750 and 820 nm, was measured via a Ti:sapphire laser.

2. *Radiation loss to the substrate*

Since the refractive index of the Si substrate was greater than that of the waveguide core, the guided mode was not fully confined, and light could gradually leak into the substrate [S63]. Here, we numerically analyze the impact of this radiation loss on a programmable nonlinear waveguide.

To model the radiation loss, we represent our system as a symmetric planar waveguide composed of an SiN core with thickness $d_{\mathrm{core}} = 2.05\,\mu\mathrm{m}$, top and bottom SiO_2 cladding layers with thickness $d_{\mathrm{cladding}} = 1\,\mu\mathrm{m}$, and Si substrates on both sides of the cladding layers, which are assumed to be infinitely thick. We then employ the formalism in Ref. [S63] to calculate the radiation loss rate κ for this stack and use $\kappa/2$ as a phenomenological estimate for our waveguide, which has an Si substrate only on one side. The simulation results for the loss at 1.55 µm are shown in Fig. S11, indicating that the contribution from radiation loss is negligible compared with the material absorption observed in our experiment.

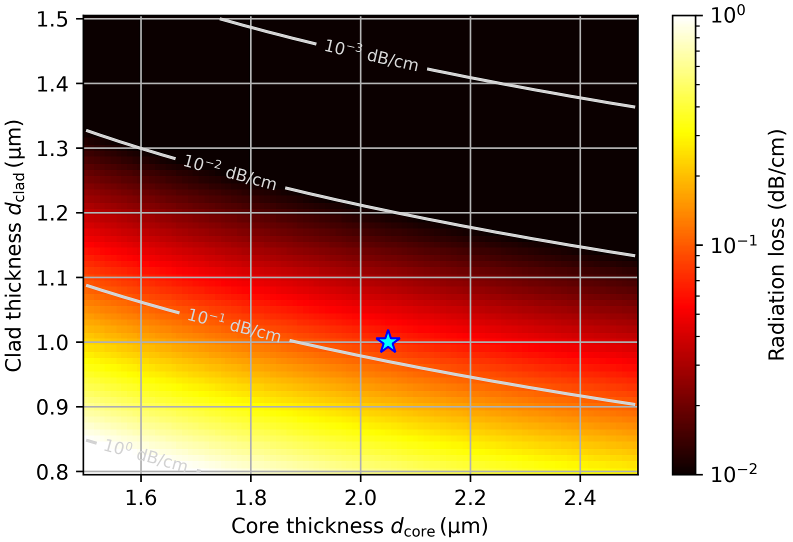

FIG. S11: Numerically simulated radiation loss to the Si substrate at a wavelength of 1.55 µm. We use the formalism provided in Ref. [S63]. The blue star represents the design of our programmable nonlinear waveguide.

3. *Coupling to the photoconductor mode*

Another loss channel is formed by the coupling between the core mode and the photoconductor mode. Unlike the coupling to the substrate discussed in Sec. S4 C 2, where the large thickness of the substrate results in a continuum of modes, the finite thickness of the photoconductor layer yields well-resolved discrete modes. Consequently, we observed sharp peaks in the waveguide loss at specific wavelengths where the effective index of the fundamental core mode matched that of a photoconductor mode.

The field distribution of a TM mode in a waveguide satisfies the following equation:

$$\beta^2 H_x(y) = \left(n^2(y)\partial_y \frac{1}{n^2(y)}\partial_y + k_0^2 n^2(y)\right) H_x(y), \tag{S31}$$

where β is the propagation constant of the mode, $n(y)$ denotes the refractive index distribution in the vertical direction y, and $H_x(y)$ is the magnetic field component in the transverse direction x. Thus, for a given planar optical waveguide stack along the y-direction, we can numerically diagonalize Eq. (S31) to obtain all the modes of the system.

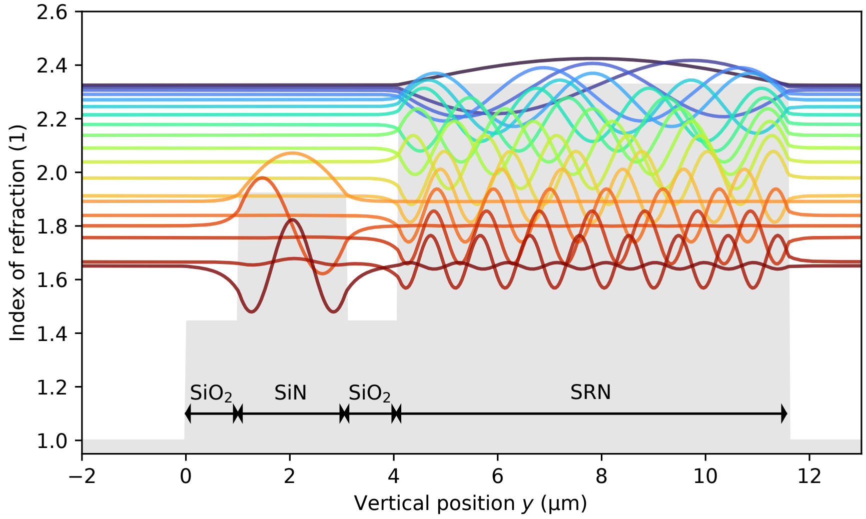

FIG. S12: Spatial profiles $H_x(y)$ of the TM modes at $\lambda_0 = 1.55\,\mu\text{m}$ calculated via Eq. (S31), where we show 18 modes with the highest effective indices. We use the same index data as in Fig. S8 with the waveguide geometry specified in the Methods section. The gray shaded region represents the distribution of the index of refraction $n(y)$.

The waveguide modes are shown in Fig. S12 for $\lambda_0 = 1.55\,\mu\text{m}$. In this figure, core modes (localized within the SiN layer) and photoconductor modes (localized within the SRN layer) are clearly observed. To focus on the physics of the photoconductor modes, we replace the Si substrate with an air layer in this model.

Generally, the effective indices of the core modes and the photoconductor mode vary in different ways with the wavelength λ_0. Consequently, as λ_0 changes, their indices may cross over. This behavior is illustrated in Fig. S13(a), which shows the effective indices of the waveguide modes as a function of λ_0. A closer look at the crossover region in Fig. S13(b) reveals an avoided energy crossing between the modes, a clear manifestation of mode hybridization.

When such hybridization occurs, light in the fundamental core mode can leak into the photoconductor mode via coherent coupling. The strength of this coupling is characterized by the magnitude of the avoided crossing in Fig. S13(b), which is approximately $\Delta n = 10^{-4}$. This implies that a significant portion of the light will be lost to the photoconductor mode over a propagation distance of $\lambda_0/\Delta n \approx 1\,\text{cm}$. Note that this photoconductor-induced loss affects only a narrow band of wavelengths near the crossover point and does not lead to global losses. Furthermore, owing to the coherent nature of the coupling, the light can also be recoupled back into the core mode after sufficient propagation.

We present the experimental results for transmission through a programmable nonlinear waveguide in Fig. S14 for various wavelengths of light. Aside from the loss at approximately 1520 nm due to material absorption (see Sec. S4 C 1), we observed localized absorption lines near 1560 nm, which we attributed to loss caused by the photoconductor mode. The effects of the photoconductor mode were also evident in the experimental results shown in Fig. 3, where the SHG conversion efficiency was reduced around this pump wavelength. Note that the location of the absorption peak is

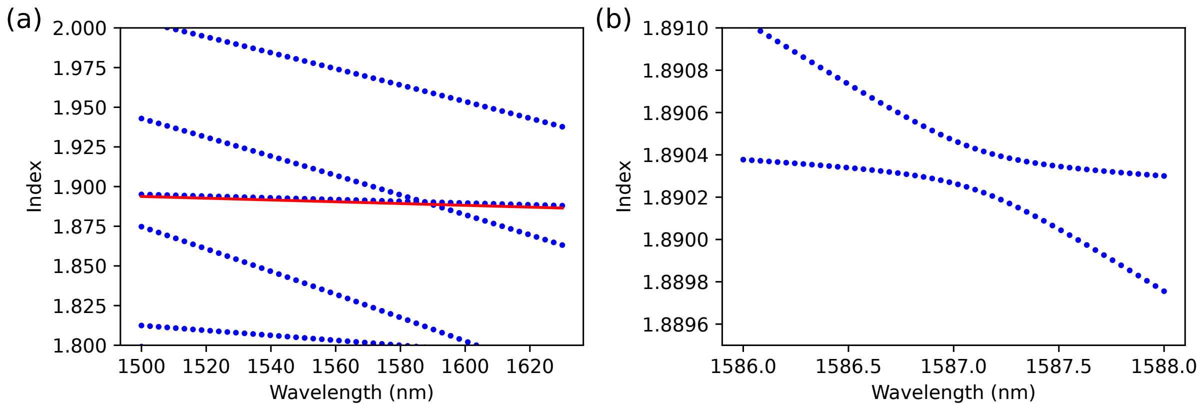

FIG. S13: The blue circles represent numerically calculated effective indices of the waveguide modes for various wavelengths λ_0, where we use the same simulation methods as in Fig. S12. The red solid lines are unperturbed indices of the fundamental TM modes in the absence of the photoconductor layer. (a) and (b) show the same data but with different regions of interest.

highly sensitive to factors such as the refractive index and thickness of the films, which likely explains the quantitative mismatch between the numerically predicted location of mode crossing and the experimental result.

Finally, we note that the photoconductor-induced loss can be mitigated by employing a thicker top cladding. As the overlap between the core modes and the photoconductor modes decreases exponentially with increasing cladding thickness, even a slight increase in the top cladding can significantly reduce these effects.

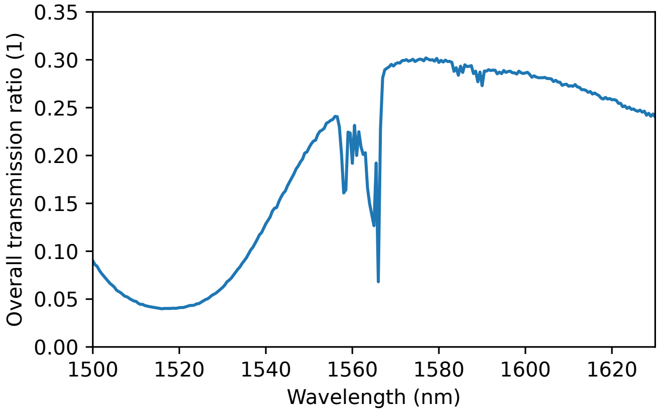

FIG. S14: Optical transmission ratio of a programmable nonlinear waveguide measured via a tunable CW laser (TSL-570; Santec). The loss includes incoupling loss to the waveguide, outcoupling loss from the waveguide, and the finite collection efficiency of the light.

4. Prospects for reducing the loss

There are various routes to reduce the absorption of SiN from the levels observed in Sec. S4 C 1. In principle, SiN is capable of achieving extremely low optical loss. The material absorption limit of SiN has been estimated to be 0.13 dB/m, and losses as low as 0.4 dB/m have been demonstrated in high-confinement SiN waveguides [S14, S39]. These films were fabricated using low-pressure chemical vapor deposition (LPCVD). For PECVD SiN, it is known that residual hydrogen atoms in the film increase absorption, especially near 1520 nm [S61, S62]. Reduction of loss in PECVD SiN is possible either by using deuterated silane [S64] or by high-temperature furnace annealing [S65], both of which reduce the hydrogen concentration in the film. Here, we present preliminary experimental results using the latter approach.

For the annealing characterization, we fabricated channel waveguides using PECVD SiN. First, NOVA electronic materials provided a Si substrate with a 1 µm bottom oxide. We then deposited 2 µm of SiN and 1 µm of SiO_2 using PECVD. For the SiN deposition, we used an RF power of 200 W with gas flows of SiH_4 : 3 sccm, H_2 : 40 sccm, and N_2 : 2000 sccm. We performed photolithography with a DUV stepper (PAS 5500; ASML) to produce a photomask. Using a plasma etcher (PlasmaPro 100 RIE; Oxford Instruments) with CH_2F_2/He gas, we etched the waveguide structure onto the top SiO_2 layer, creating an oxide hard mask. Next, we etched SiN with $CHF_3/O_2/N_2$ gas. Finally, we deposited 1 µm of SiO_2 with PECVD, and we performed a 3 hour furnace anneal at 1200 °C with N_2 flow. The final die contained two types of waveguides: straight waveguides with a length of 2 cm and a spiral waveguide with a total length of 6 cm. In Fig. S15, we show a microscope image of the spiral waveguide.

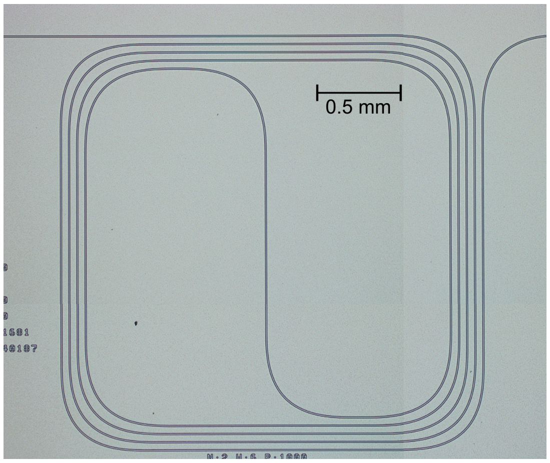

FIG. S15: A microscope image of the SiN spiral waveguide. The image was created by stitching together four images with smaller fields of view to capture the entire waveguide.

For the measurement of optical loss, we coupled a CW laser with a tunable wavelength between 1500 nm and 1630 nm into the waveguides and measured the power at the output. By dividing the ratio of output power by the difference in the lengths of the waveguides, we estimated the propagation loss per unit length. The measurement results are shown in Fig. S16. Though the data came out slightly noisy, potentially due to the multi-mode nature of the waveguide, we observed a clear reduction in optical loss—down to 0.4 dB/cm for wavelengths $>$ 1550 nm and to 0.8 dB/cm around 1520 nm, where we had previously observed losses of 5 dB/cm. In the future, these loss values may be further improved by optimizing the etching recipe to reduce contributions from sidewall roughness.

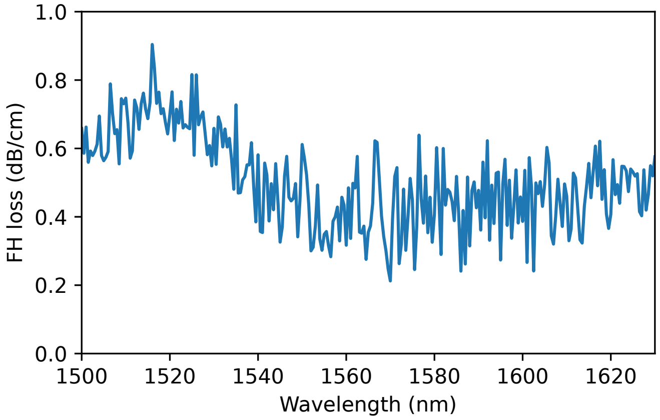

FIG. S16: Optical loss of PECVD SiN waveguides after furnace annealing, estimated by comparing the power transmission through two waveguides of different lengths. We used a straight channel waveguide with a length of 2 cm and a spiral waveguide with a length of 6 cm.

D. Optical damage threshold

Here, we comment on the amount of power that the programmable nonlinear waveguides can handle, which provides a rough estimate of the range of applications for which the device may be useful. Using 250 fs ultrashort pulses, Ref. [S66] reported a damage threshold of 0.19 J/cm^2, corresponding to 7.6 GW/mm^2. For a SiN planar waveguide with a cross-sectional mode area of 2 µm × 150 µm, the damage threshold corresponds to 2.3 MW. At half this damage threshold and using the normalized conversion efficiency of $\eta_{\mathrm{norm}} = 5 \times 10^{-5}$ %/W, a value demonstrated in this work, the total conversion efficiency would be $\eta_{\mathrm{tot}} = 6 \times 10^{1}$ %. For the programmable channel waveguide shown in Methods, the approximate cross-sectional area is 2 µm × 4 µm, leading to a damage threshold of 61 kW. With the observed normalized conversion efficiency of $\eta_{\mathrm{norm}} = 2 \times 10^{-3}$ %/W and operating at half of the damage threshold, the total conversion efficiency becomes $\eta_{\mathrm{tot}} = 6 \times 10^{1}$ %.

S5. EXPERIMENTAL ESTIMATION OF ELECTRIC-FIELD-INDUCED $\chi^{(2)}$ NONLINEARITY

In this section, we estimate the electric-field-induced $\chi^{(2)}$ nonlinearity in a programmable waveguide on the basis of the experimentally measured CW-pumped SHG conversion efficiency. Assuming that the pump field remains undepleted during SHG and that the effects of spatial diffraction are negligible, the evolution of the SH field can be written as

$$\partial_z b = -i\kappa e^{-i\Delta k z} r(x,z) a^2, \tag{S32}$$

where the definitions of κ and $r(x,z)$ are provided in Sec. S6. When a monotonic QPM grating pattern with the correct period is projected to achieve quasi-phase matching, the nonlinearity distribution takes the form of a square wave

$$r_{\mathrm{square}}(x,z) = \frac{1}{2}\Big(\mathrm{sign}(\sin(\Delta k z)) + 1\Big) = \frac{1}{2} + \frac{1}{i\pi}\sum_{\ell=1}^{\infty}\frac{1}{2\ell-1}\Big(e^{i(2\ell-1)\Delta k z} - e^{-i(2\ell-1)\Delta k z}\Big). \tag{S33}$$

Since only the Fourier components with spatial frequencies close to Δk contribute significantly to SHG, if the waveguide is sufficiently long, the equation of motion for the SH field can be approximated as

$$\partial_z b \approx -\kappa_{\mathrm{eff}} a^2, \tag{S34}$$

with

$$\kappa_{\mathrm{eff}} = \frac{1}{\pi}\kappa. \tag{S35}$$

With the undepleted pump approximation, we can analytically integrate this equation to obtain

$$b(x, z = L_{\mathrm{QPM}}) = \kappa_{\mathrm{eff}}\, a^2(x, z=0)\, L_{\mathrm{QPM}},$$

where L_{QPM} is the quasi-phase-matched distance.

We parameterize the field profile of the input FH field as

$$|a(x,0)|^2 = \frac{\sqrt{2}P_{\mathrm{FH}}}{\sqrt{\pi}w}\, e^{-2x^2/w^2}, \tag{S36}$$

which has a total power flux of P_{FH}. Under this condition, we obtain

$$|b(x, z = L_{\mathrm{QPM}})|^2 = \frac{2\kappa_{\mathrm{eff}}^2 L_{\mathrm{QPM}}^2 P_{\mathrm{FH}}^2}{\pi w^2}\, e^{-4x^2/w^2}. \tag{S37}$$

Integrating over the transverse coordinate yields

$$\eta_{\mathrm{norm}} = \frac{P_{\mathrm{SH}}}{P_{\mathrm{FH}}^2} = \frac{\kappa^2 L_{\mathrm{QPM}}^2}{\pi^{5/2} w}, \tag{S38}$$

which establishes the relationship between the experimentally measurable η_{norm} and the nonlinear coupling κ.

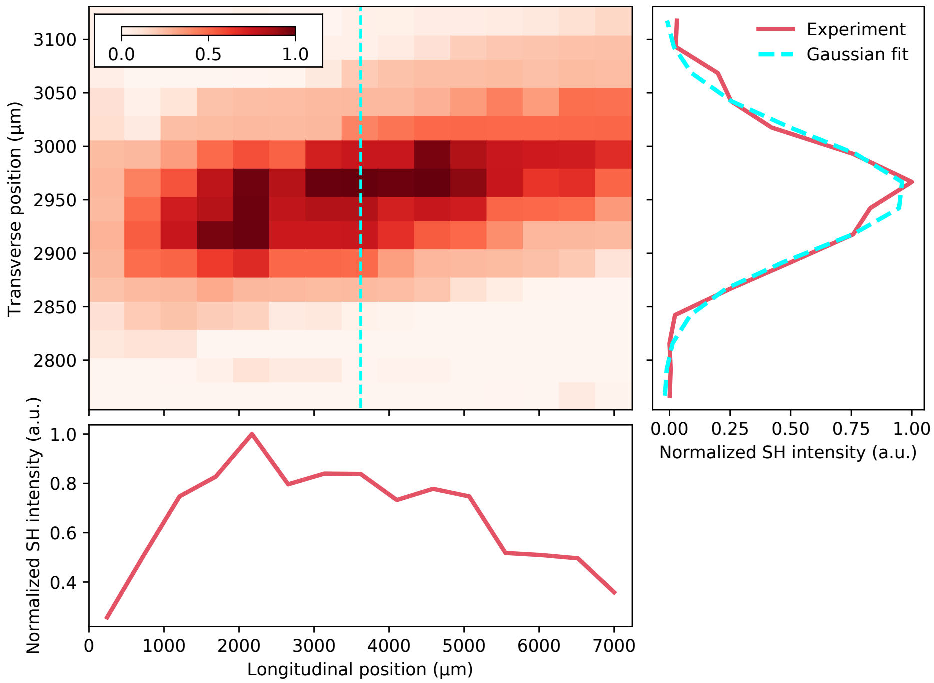

FIG. S17: Experimentally measured contributions to SHG from different regions of a programmable nonlinear waveguide. The data were acquired with a pulse pump laser via the measurement technique described in Sec. S10 A. The cyan line on the right top panel is the Gaussian fit (with offset) of the cross section at the vertical cyan line in the left top panel. Similarly, the cyan line on the left bottom panel is the Gaussian fit (with offset) of the cross section at the horizontal cyan line in the left top panel.

In Fig. S17, we show the experimentally mapped distributions of the SHG contributions from different regions of the waveguide. From a Gaussian fit to the beam profile, we obtained an SH beam width of 108 μm, which corresponded to $w = 152\,\mu\text{m}$ in Eq. (S36).

To estimate the maximum $\chi^{(2)}$ nonlinearity inducible on our platform, we projected a monotonic grating pattern with a varying period Λ and measured the SHG conversion efficiency when pumping the waveguide with a CW laser at a wavelength of $\lambda = 1580\,\text{nm}$. We set the bias voltage to $V_{\text{bias}} = 1600\,\text{V}$ at a frequency of 5 Hz. The measurement result is shown in Fig. S18, where we observed a maximum conversion efficiency of $\eta_{\text{norm}} = 1.51 \times 10^{-5}\,\%\text{W}^{-1}$. Note that in this measurement, we limited the projection of the grating pattern to the region $1509.6\,\mu\text{m} \leq z \leq 5283.6\,\mu\text{m}$, corresponding to $L_{\text{QPM}} = 3772\,\mu\text{m}$. This restriction avoided the nonuniform nonlinearity in the longitudinal direction observed in Fig. S17. The good agreement between the experimental data and the theoretical sinc curve—assuming $L_{\text{QPM}} = 3772\,\mu\text{m}$ in Fig. S18—indicates that the $\chi^{(2)}$ nonlinearity was mostly uniform within this window.

Now, we could estimate the value of the nonlinearity by solving Eq. (S38) for $\chi^{(2)}_{yyy}$ with Eq. (S50). For this purpose, we used numerically calculated waveguide parameters for the fundamental TM modes: $L_{\text{eff}} = 1.693\,\mu\text{m}$, $n^{(\omega)}_{\text{eff}} = 1.891$, and $n^{(2\omega)}_{\text{eff}} = 1.938$. Overall, we obtained $\chi^{(2)}_{yyy} = 0.47\,\text{pm/V}$ as our best estimate of the measured $\chi^{(2)}$ nonlinearity. As discussed in Sec. S3, improvements in the photoconductor can increase the induced nonlinearity by a factor of $\mathcal{R}_{\max} \approx 2.3$, suggesting that $\chi^{(2)}_{yyy} = 1.1\,\text{pm/V}$ could be physically feasible.

Although we present our estimates to two significant figures, it is well known that estimating nonlinear coefficients is notoriously difficult even for bulk materials, so we do not expect our estimates to be accurate to better than a factor of 2. Possible sources of error include uncertainty in the off-chip collection efficiency, contributions from nonlinear tensor elements other than $\chi^{(2)}_{yyy}$, fringing of the bias electric field inside the core (see Ref. [S17]), and variations in the spatial beam profiles on the waveguide.

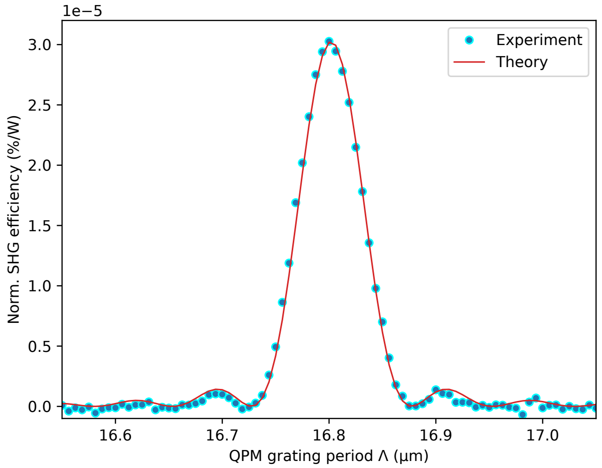

FIG. S18: Experimentally measured η_{norm} with a pump wavelength of 1580 μm. A bias voltage of $V_{\text{tot}} = 1600\,\text{V}$ was used. The red solid lines represent the theoretical curve $\propto \text{sinc}^2(\pi(\Lambda - \Lambda_0)L_{\text{QPM}})$ with $\Lambda_0 = 16.802\,\mu\text{m}$ and $L_{\text{QPM}} = 3772\,\mu\text{m}$.

S6. MODEL FOR SHG IN A PROGRAMMABLE NONLINEAR WAVEGUIDE

In this section, we derive a model for SHG in a programmable nonlinear waveguide by formulating equations of motion for the FH and SH beams. We adapt the formalism from Ref. [S67] to the case of a planar waveguide.

The electric and magnetic field profiles of the fundamental TM modes can be written as functions of the vertical coordinate, i.e., $\mathbf{E}(y)$ and $\mathbf{H}(y)$ (see Sec. S4). Using Poynting's theorem, we impose the following normalization condition for the mode:

$$\frac{1}{2}\int \mathrm{d}y\,\mathrm{Re}\Big(\mathbf{E}(y)\times\mathbf{H}^*(y)\Big)\cdot\mathbf{z} = W, \tag{S39}$$

where $\mathbf{z}$ is a unit vector in the longitudinal direction and W is a normalization constant with units of one-dimensional power density [power $\cdot$ length^{-1}]. We then express the field profiles as

$$\mathbf{E}(y) = \sqrt{\frac{2Z_0 W}{n_{\text{eff}}L_{\text{mode}}}}\,\mathbf{e}(y), \qquad \mathbf{H}(y) = \sqrt{\frac{2n_{\text{eff}}W}{Z_0 L_{\text{mode}}}}\,\mathbf{h}(y), \tag{S40}$$

where $Z_0 = 377\,\Omega$ is the vacuum impedance, and $\mathbf{e}(y)$ and $\mathbf{h}(y)$ are dimensionless field profiles. The width of the mode in the vertical direction is characterized as

$$L_{\text{mode}} = \int \mathrm{d}y\,\mathrm{Re}\Big(\mathbf{e}(y)\times\mathbf{h}(y)\Big)\cdot\mathbf{z}. \tag{S41}$$

Note that Eqs. (S40) and (S41) are defined to be consistent with the normalization condition Eq. (S39), which leaves the scaling of $\mathbf{e}(y)$ and $\mathbf{h}(y)$ as a free parameter. Following a convention in NLO, we set the scaling of $\mathbf{e}(y)$ and $\mathbf{h}(y)$ so that the peak value of $\mathrm{Re}\big(\mathbf{e}(y)\times\mathbf{h}(y)\big)\cdot\mathbf{z}$ is unity.

In the remainder of this section, we assume that only the fundamental TM modes of the FH and SH light, with frequencies ω and 2ω, respectively, are excited. The electromagnetic fields can be parameterized as

$$\mathbf{E}(x,y,z,t) = \frac{1}{\sqrt{W}}\int \mathrm{d}x\,\Big[\mathbf{E}^{(\omega)}(y)\,a(x,z)\,e^{-i\omega t + ik_1 z} + \mathbf{E}^{(2\omega)}(y)\,b(x,z)\,e^{-2i\omega t + ik_2 z}\Big], \tag{S42}$$

where we explicitly label the waveguide modes by their frequencies. Here, $a(x,z)$ and $b(x,z)$ are the spatial amplitudes of the FH and SH fields, respectively, with units of [power$^{1/2}$ $\cdot$ length$^{-1/2}$].

The evolution of the field amplitudes follows

$$\partial_z a(x,z) = \frac{i}{2k_1}\partial_x^2 a(x,z) - \frac{i\omega}{4\sqrt{W}}\,e^{-ik_1 z}\int \mathrm{d}y\,\mathbf{E}^{(\omega)*}(y)\cdot\mathbf{P}_{\text{NL}}^{(\omega)}(x,y,z), \tag{S43}$$

$$\partial_z b(x,z) = \frac{i}{2k_2}\partial_x^2 b(x,z) - \frac{i\omega}{2\sqrt{W}}\,e^{-ik_2 z}\int \mathrm{d}y\,\mathbf{E}^{(2\omega)*}(y)\cdot\mathbf{P}_{\text{NL}}^{(2\omega)}(x,y,z). \tag{S44}$$

The nonlinear polarizations at the respective frequencies are given by

$$P_{\mathrm{NL},i}^{(\omega)}(x,y,z) = \frac{2\epsilon_0\, b(x,z)\, a^*(x,z)}{W} \sum_{jk} d_{ijk}\, E_j^{(2\omega)}(y)\, E_k^{(\omega)*}(y)\, e^{ik_2 z - ik_1 z}, \tag{S45}$$

$$P_{\mathrm{NL},i}^{(2\omega)}(x,y,z) = \frac{\epsilon_0\, a^2(x,z)}{W} \sum_{jk} d_{ijk}\, E_j^{(\omega)}(y)\, E_k^{(\omega)}(y)\, e^{2ik_1 z}, \tag{S46}$$

where the indices i,j,k run over the coordinate axes x,y,z. To further evaluate the model, we make the simplifying assumption that only the vertical component of the induced $\chi^{(2)}$ nonlinearity predominantly contributes to SHG between the TM modes. That is, we assume that $d_{ijk}=0$ except when $i=j=k=y$ [S9]. The programmed spatial distribution of the nonlinearity is generally denoted as

$$d_{yyy}(x,y,z) = \begin{cases} \frac{\chi_{yyy}^{(2)}}{2}\, r(x,z) + \frac{\chi_{\mathrm{const}}^{(2)}}{2} & \text{for } |y| \le d_{\mathrm{core}}/2, \\ 0 & \text{otherwise,} \end{cases} \tag{S47}$$

with the maximum programmable nonlinearity $\chi_{yyy}^{(2)}$. The function $0 \le r(x,z) \le 1$ represents the dynamically programmable distribution of $\chi^{(2)}$ nonlinearity. We assume that any contribution from the cladding to the nonlinearity is negligible. The constant background nonlinearity $\chi_{\mathrm{const}}^{(2)}$ is caused by the non-zero bias electric field that is present even when the programming illumination is off. While the absolute maximum $\chi^{(2)}$ nonlinearity on the device is $\chi_{\mathrm{tot}}^{(2)} = \chi_{yyy}^{(2)} + \chi_{\mathrm{const}}^{(2)}$, only the programmable part with spatial variation can contribute to meaningful nonlinear-optical processes. This is because the contributions from $\chi_{\mathrm{const}}^{(2)}$ average out over propagation due to phase mismatch. Therefore, we ignore the contribution from $\chi_{\mathrm{const}}^{(2)}$ below unless otherwise specified. By using a thicker photoconductor layer, we can reduce the background nonlinearity $\chi_{\mathrm{const}}^{(2)}$ and realize programmable nonlinearity approaching $\chi_{\mathrm{tot}}^{(2)}$.

Overall, we obtain

$$\partial_z a = \frac{i}{2k_1}\partial_x^2 a - i\kappa\, e^{i\Delta k\, z}\, r(x,z)\, a^* b, \tag{S48}$$

$$\partial_z b = \frac{i}{2k_2}\partial_x^2 b - i\kappa\, e^{-i\Delta k\, z}\, r(x,z)\, a^2, \tag{S49}$$

with a nonlinear coupling given by

$$\kappa = \frac{\epsilon_0 \omega \chi_{yyy}^{(2)}}{4\sqrt{W^3}} \left(\int_{\mathrm{core}} \mathrm{d}y\, E_y^{(2\omega)*} (E_y^{(\omega)})^2 \right) = \frac{\omega \chi_{yyy}^{(2)}}{2c\sqrt{L_{\mathrm{eff}}}} \sqrt{\frac{2Z_0}{n_{\mathrm{eff}}^{(\omega)2} n_{\mathrm{eff}}^{(2\omega)}}}. \tag{S50}$$

The effective mode width is defined as

$$L_{\mathrm{eff}} = \frac{\left(L_{\mathrm{mode}}^{(\omega)}\right)^2 L_{\mathrm{mode}}^{(2\omega)}}{\left[\int_{\mathrm{core}} \mathrm{d}y\, e_y^{(2\omega)} \left(e_y^{(\omega)}\right)^2\right]^2}. \tag{S51}$$

The equations of motion Eqs. (S48) and (S49) are the main results of this section and can be used to simulate the SHG dynamics for a given distribution of $\chi^{(2)}$ nonlinearity, i.e., $r(x,z)$.

S7. COMMON PARTS OF THE EXPERIMENT

In this section, we describe the parts of the experimental setup that were commonly used for all the experiments in this work.

A. Projector setup for programming illumination

The details of the projector setup used to produce the programming illumination are described here. A photograph of the setup is shown in Fig. S19. The primary light source was a green diode laser with a wavelength of 532 nm. To

clean the spatial mode, the laser output was first focused through a pinhole and then collimated with a lens. The spatial dimensions of the beam were tailored via an anamorphic prism pair and a beam expander. The anamorphic prism pair expanded the beam in the horizontal direction, and the beam expander adjusted the overall beam size.

The spatial intensity was modulated via a spatial light modulator (SLM-200-01; Santec) in combination with a polarization beam splitter and a half-wave plate. The SLM had a resolution of 1920×1200 pixels, each with a pitch of $8\,\mu\text{m}$, and supported 10-bit grayscale resolution. The SLM pattern was projected onto the surface of the programmable nonlinear waveguide via a macro camera lens (Milvus 100 mm f/2M Lens; Carl Zeiss). The demagnification ratio of the setup was determined by imaging a test grating with a known period onto a monitoring camera, which was placed at the same distance from the test grating surface as the SLM. Through this calibration process, we measured the demagnification ratio as 2.1209, meaning that each $8\,\mu\text{m}$ pixel on the SLM corresponds to a feature size of $3.772\,\mu\text{m}$ on the waveguide surface. The illumination intensity on the chip surface is approximately $50\,\text{mW}/\text{cm}^2$, which is low enough to avoid causing breakdown damage to the films.

The update speed of the illumination pattern was limited by the SLM response time; typically, the system required approximately 1 s to reach a steady state after the SLM pattern was updated. This limitation can be addressed by employing a faster SLM.

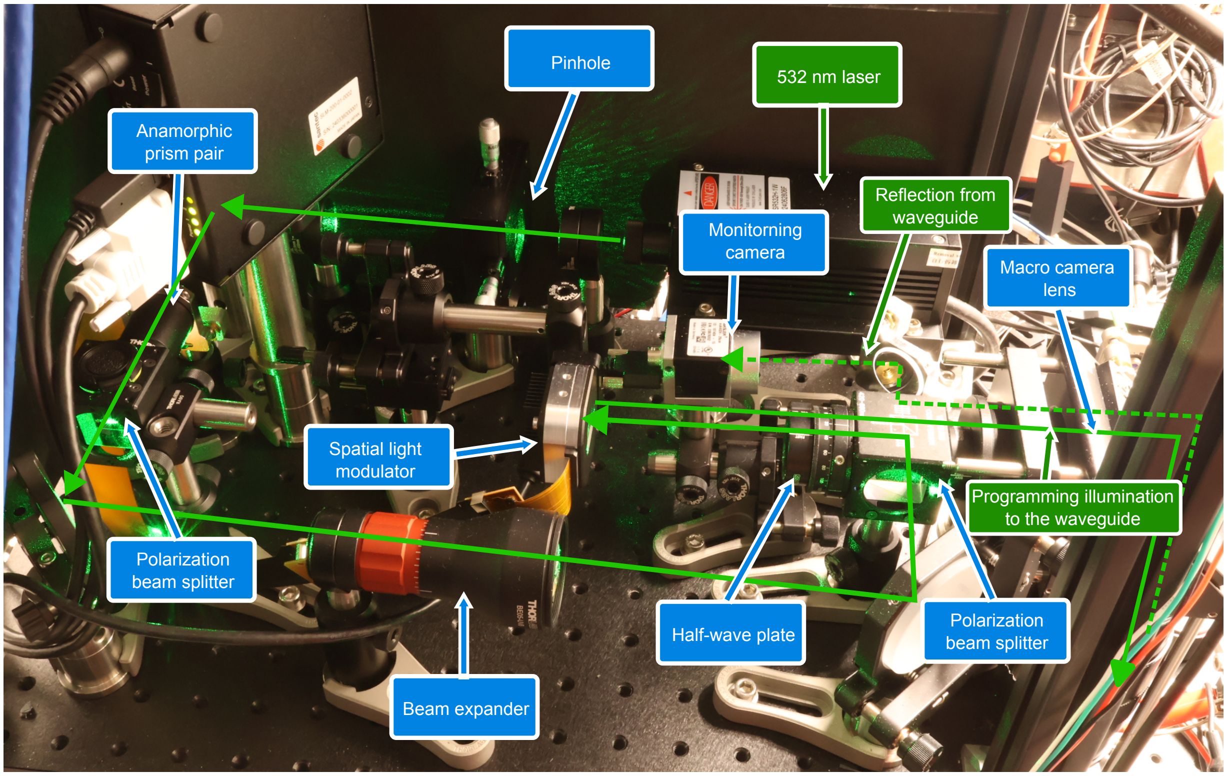

FIG. S19: Photograph of the projector setup used to generate the programming illumination. The solid green lines indicate the forward laser path toward the surface of the programmable nonlinear waveguide, and the dashed green lines indicate the reflected light path from the waveguide surface. The light blue boxes and arrows indicate the essential optical components.

B. Electrical and optical coupling to the waveguide

As shown in Fig. S20, a programmable nonlinear waveguide was mounted on a micrometer translation stage. The programming illumination from the projector setup (see Sec. S7 A) was applied to the top surface of the waveguide. From the side, a pump laser focused by an aspheric focusing lens (C660TME-C; Thorlabs) was coupled to the waveguide. Bias electric fields were applied via a pair of electrodes, one attached to the top transparent electrode and the other connected to the waveguide substrate. The electrically induced $\chi^{(2)}$ nonlinearity produced the SHG, which was collected by an objective lens. Various objective lenses were used depending on the experimental requirements, as described later.

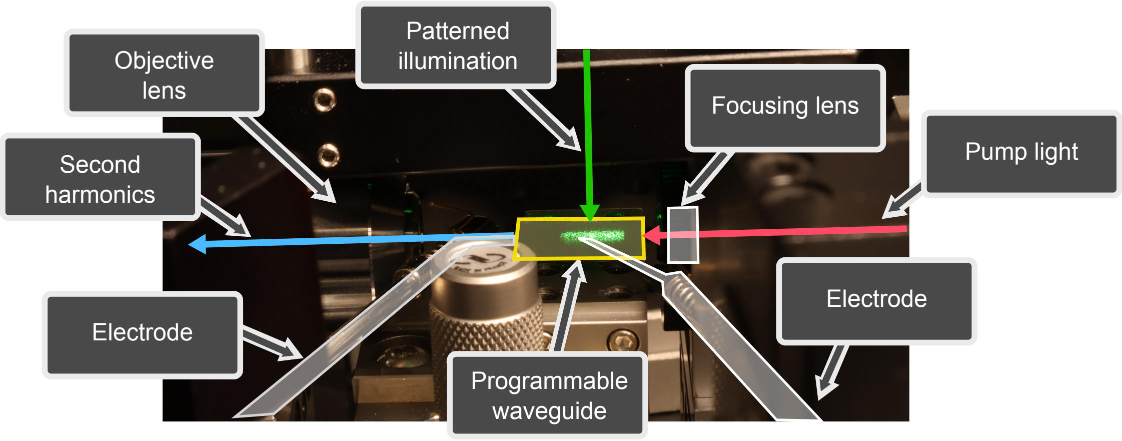

FIG. S20: Main part of the experimental setup, where we programmed the nonlinearity, pumped the waveguide, and collected the generated second harmonics.

S8. PROGRAMMABLE PERIODIC POLING FOR CW-PUMPED SHG

This section describes the experimental details of the results of CW-pumped SHG presented in the main text.

A. Calibration of the experimental setup

In Fig. S21, we show an illustration of the experimental setup. The pump light from a CW laser (TSL-570; Santec), with a wavelength tunable between $\lambda = 1500\,\mathrm{nm} \sim 1630\,\mathrm{nm}$, was coupled into a programmable nonlinear waveguide via a pair of focusing lenses. The combination of the first cylindrical lens and a focusing aspheric lens approximately collimated the beam in the horizontal (i.e., x-) direction while tightly focusing it in the vertical (i.e., y-) direction. The generated SH light was then collimated via an aspheric lens (used as an objective) and a cylindrical lens and was detected via a photomultiplier tube (PMT) through short-pass filters that rejected the pump light.

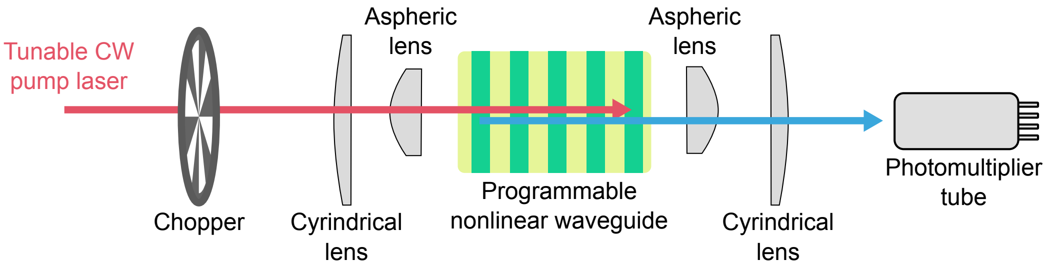

FIG. S21: Illustration of the CW-pumped programmable SHG experiment. On the input side, the cylindrical lens had a focal length of $f = 25\,\mathrm{mm}$, and the aspheric lens was a C660TME-C (Thorlabs). For the output side, we used a cylindrical lens and an aspheric lens C330TME-B (Thorlabs). The generated SH signal was detected via a PMT (PMT H10722-20; Hamamatsu Photonics) with a control voltage of $V_{\mathrm{control}} = 0.5\,\mathrm{V}$.

We applied a total bias voltage of $V_{\mathrm{tot}} = 1000\,\mathrm{V}$ at a frequency of 5 Hz unless otherwise specified, which was found to be approximately optimal (see Sec. S3). During the measurement, an optical chopper wheel modulated the pump light, and we used the difference between the on- and off-state signals as the measurement outcome. This lock-in-like procedure allowed us to reject DC noise in the signal. The raw data were recorded as the signal voltage from the PMT, which we converted to obtain the normalized SHG conversion efficiency. Several calibration and normalization procedures were required for this conversion.

First, we calibrated the sensitivity of the PMT, including the losses incurred by the filters. A measurement using

a power reference at 780 nm yielded a sensitivity of 1.34×10^9 V/W. Using this value, we converted the signal voltage from the PMT to the detected SH power $P_{\rm SH}^{\rm detected}$. To determine the SH power generated inside the chip, we accounted for the collection efficiency, $R^{\rm collect} = 75\,\%$, from the chip to free space, which was independently calibrated. Additionally, the propagation loss of the SH was estimated as

$$R_{\rm SH}^{\rm prop} = \exp(-\alpha_{\rm SH}(L_{\rm tot} - L_{\rm SHG})), \tag{S52}$$

where $L_{\rm tot} = 1.8$ cm is the total length of the waveguide and $L_{\rm SHG} = 0.6$ cm is the nominal location of the SHG on the chip measured from the input facet. We use the material loss characterized in Sec. S4 C to obtain the attenuation coefficient $\alpha_{\rm SH}$. Finally, the $\chi^{(2)}$ nonlinearity was modulated sinusoidally by the AC bias electric field, so the peak SHG power was twice its average. Overall, the calibrated SH power was given by

$$P_{\rm SH} = \frac{2\,P_{\rm SH}^{\rm detected}}{R^{\rm collect}\,R_{\rm SH}^{\rm prop}}. \tag{S53}$$

Second, we estimated the pump power $P_{\rm FH}$ that effectively contributed to SHG. To do this, we first measured the FH power $P_{\rm FH}^{\rm detected}$, accounting for the collection efficiency $R^{\rm collect}$, as shown in Fig. S22. Notably, the dips around 1560 nm were caused by leakage into the photoconductor mode (see Sec. S4 C 3). Owing to the coherent nature of this leakage, we cannot simply apply an exponential decay model to estimate $P_{\rm FH}$. Instead, we used smooth interpolation over the dip to obtain $P_{\rm FH}^{\rm corrected}$, as shown in the figure. We then accounted for the propagation loss

$$R_{\rm FH}^{\rm prop} = \exp(-\alpha_{\rm FH}(L_{\rm tot} - L_{\rm SHG})) \tag{S54}$$

by using the material absorption described in Sec. S4 C 1 for $\alpha_{\rm FH}$. Overall, we obtained

$$P_{\rm FH} = \frac{P_{\rm FH}^{\rm corrected}}{R_{\rm FH}^{\rm prop}}. \tag{S55}$$

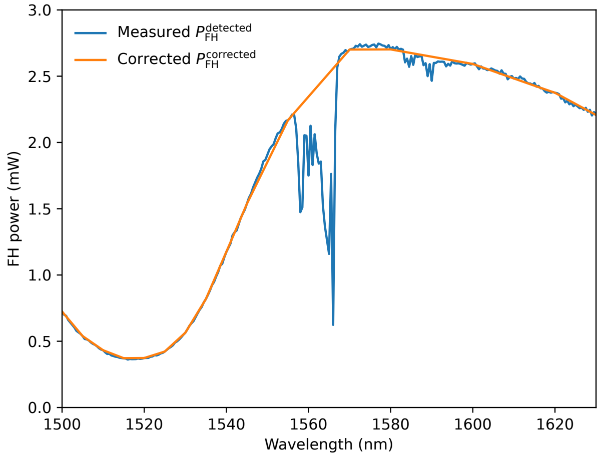

FIG. S22: Experimentally measured pump power accounting for the collection efficiency $P_{\rm FH}^{\rm detected}$(blue lines) and the pump power $P_{\rm FH}^{\rm corrected}$ corrected via interpolation over the region with photoconductor-induced loss around 1560 nm (orange lines).

Combining all the measurements, we obtained our best estimate for the normalized SHG conversion efficiency

$$\eta_{\rm norm} = \frac{P_{\rm SH}}{P_{\rm FH}^2}. \tag{S56}$$

In our programmable nonlinear waveguide, the value of $\eta_{\rm norm}$ was lower than that of a ridge waveguide with the same nonlinearity because of the relatively loose transverse confinement inherent to the planar waveguide geometry.

B. Basic nonlinear-optical characterization of the device

To produce the data shown in Fig. 2(b), we projected grating patterns with periods ranging from $\Lambda = 16.3\,\mu\text{m}$ to $16.9\,\mu\text{m}$, and we linearly scanned the pump wavelength, λ, while measuring the signal with a photodetector. The measured signal was converted to a normalized efficiency via the procedures described in Sec. S8 A. We fit sinc functions to the measured peaks, which allowed us to obtain pairs of poling periods Λ and pump wavelengths λ at which the SHG was phase-matched. Fitting the relationship between Λ and λ as a quadratic function around $\lambda = 1560\,\text{nm}$ yielded an optimal poling period of $\Lambda = 16.685\,\mu\text{m}$ and a group velocity mismatch (GVM) between the fundamental and second harmonics of $-92\,\text{fs/mm}$. Notably, this direct measurement of the GVM on a single device was enabled by the programmability of the poling period, Λ.

C. Real-time feedback to compensate for random walks in the pump wavelength

In Fig. 2(c), we present a proof-of-concept demonstration of the utility of programmable nonlinearity by using programmable poling to compensate for random fluctuations in the pump laser wavelength. As shown in Fig. S23, the experiment proceeded in discrete iteration steps. At the beginning of the jth step, we inherited the pump wavelength λ_{j-1} and the best estimate for the phase mismatch Δk_{j-1} from the previous $(j-1)$th step.

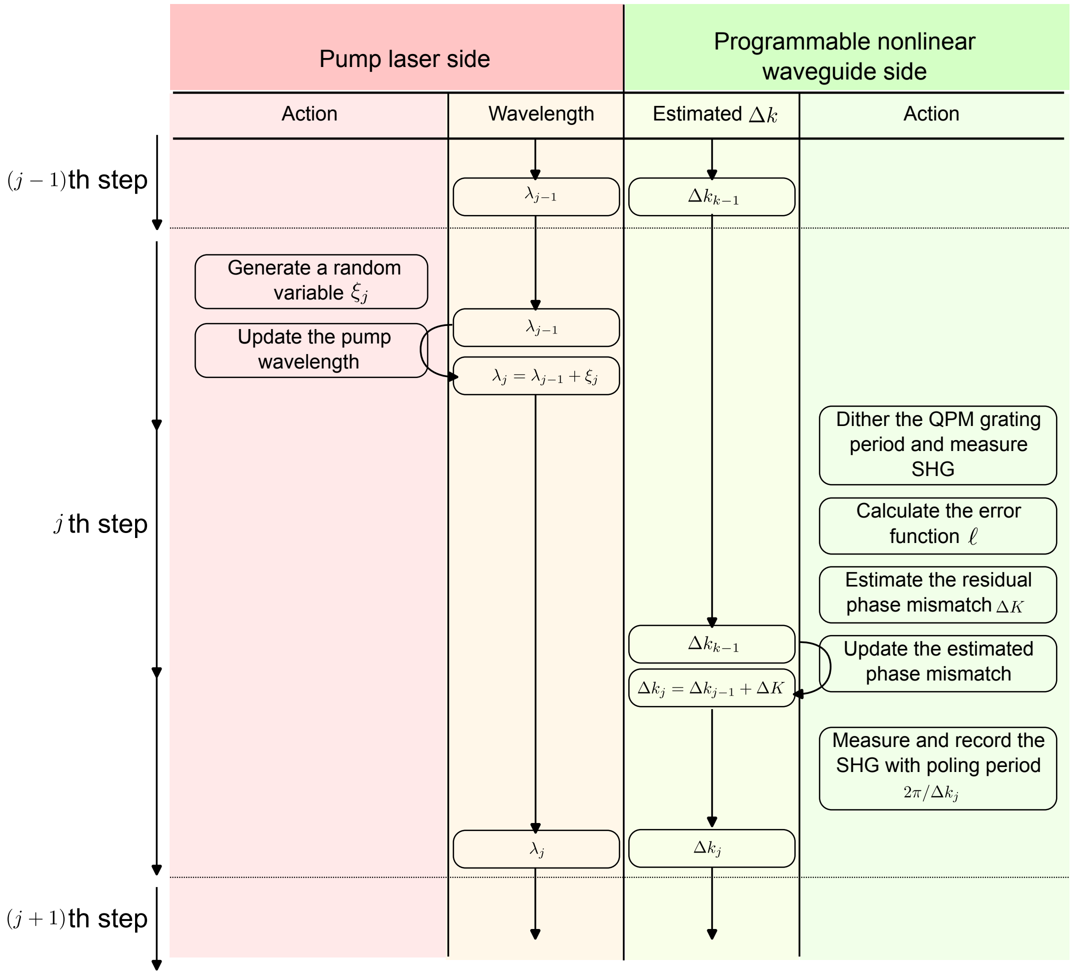

FIG. S23: Illustration of the experimental sequence used to implement real-time feedback to compensate for random walks in the pump wavelength.

First, on the side of the pump laser, we applied artificial shifts to the pump wavelength to emulate a random walk. This was achieved by generating a random number ξ_j and updating the pump wavelength to $\lambda_j = \lambda_{j-1} + \xi_j$. The

random variable ξ_j followed a Gaussian distribution with a standard deviation of 1 nm, so the sequence $\{\lambda_j\}$ followed a Gaussian random walk process. We denote the phase mismatch for the SHG at λ_j as $\Delta \tilde{k}_j$.

Then, on the side of the programmable nonlinear waveguide, we leveraged programmability to estimate a poling period that maximized the SHG efficiency for the drifted pump wavelength. Since the programmable nonlinear waveguide did not have knowledge of λ_j, the value of $\Delta \tilde{k}_j$ was initially unknown. To estimate this value experimentally, we dithered the poling period on the programmable waveguide by measuring the SHG signals at two poling periods, $\Lambda_\pm = 2\pi/(\Delta k_{j-1} \pm \epsilon)$, where ϵ is a small positive constant. Theoretically, the measured SHG powers were expected to follow

$$P_\pm = c\,\mathrm{sinc}^2\left[\frac{1}{2}(\Delta K \mp \epsilon) L_{\mathrm{QPM}}\right], \tag{S57}$$

where c is a positive constant, L_{QPM} is the length of the phase-matched region, and $\Delta K = \Delta \tilde{k}_j - \Delta k_{j-1}$ represents the error in our estimated phase mismatch. Intuitively, if $P_+ > P_-$ ($P_+ < P_-$), then we were underestimating (overestimating) the value of $\Delta \tilde{k}_j$, and this indicated how the estimate should be updated. We formally defined an error function

$$\ell = \log \frac{P_+}{P_-} \tag{S58}$$

to quantify this imbalance. To maximize the sensitivity of ℓ, we chose $\epsilon = \pi/L_{\mathrm{QPM}}$, which yielded

$$\ell(\Delta K) = \log\Big(\mathrm{sinc}^2\big(\Delta K L_{\mathrm{QPM}}/2 - \pi/2\big)\Big) - \log\Big(\mathrm{sinc}^2\big(\Delta K L_{\mathrm{QPM}}/2 + \pi/2\big)\Big). \tag{S59}$$

Note that the error function Eq. (S59) depends only on ΔK. Thus, by experimentally measuring ℓ, we could invert Eq. (S59) to obtain an estimate for ΔK, and we updated our phase mismatch estimate to $\Delta k_j = \Delta k_{j-1} + \Delta K$. In Fig. S24, we show a plot of ℓ. Finally, we measured the SHG efficiency via the updated poling period, $2\pi/\Delta k_j$, and this normalized SHG efficiency is shown in Fig. 2(c).

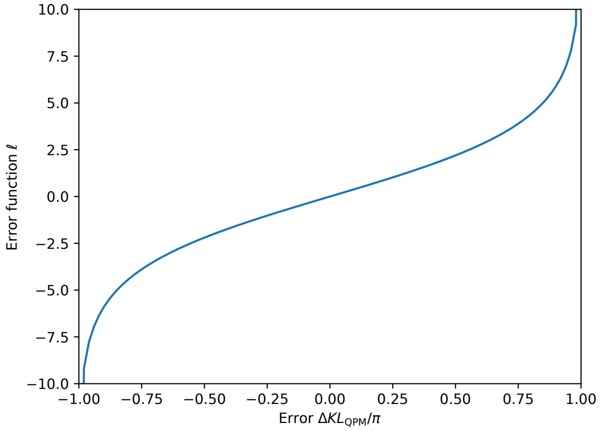

FIG. S24: Numerically calculated error function Eq. (S59).

In the experiment that produced the data for Fig. 2(c), we used $L_{\mathrm{QPM}} = 6500\,\mu\mathrm{m}$. We had 300 iteration steps, and each iteration step took 9.3 s.

S9. SPECTRAL ENGINEERING

In this section, we present the experimental details of the results on spectral engineering shown in the main text. Specifically, we explain how the data presented in Fig. 3 were experimentally produced.

A. Broadband SHG with manually designed QPM gratings

First, we describe how we produced the data in Fig. 3(a), where manually designed QPM grating structures were used to obtain programmable broadband SHG. An illustration of the experimental setup is shown in Fig. S25.

As shown in the figure, we pumped a programmable nonlinear waveguide using a pulse laser. On the output side, a spectrometer measured the spectrum of the generated SH light as we varied the structure of the patterned illumination on the waveguide surface. We used the bias voltage of $V_{\text{tot}} = 500\,\text{V}$ for this experiment. The resulting spectra in Fig. 3(a) were normalized with respect to the peak value in each plot.

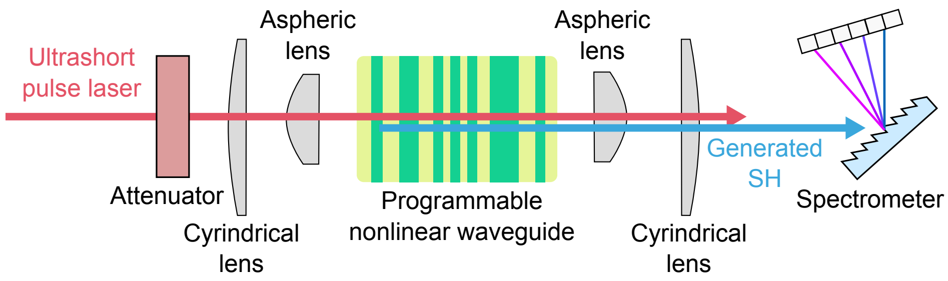

FIG. S25: Illustration of the experimental setup used to perform a broadband SHG experiment. The lens systems were the same as those in Fig. S21. The programmable nonlinear waveguide was pumped by a pulse laser (ELMO-HP; Menlo systems) with a pulse duration of $\approx$ 45 fs and an average power of 180 mW. The pump light was attenuated by a neutral density filter before coupling to the waveguide. The spectrum of the generated SH light was measured with a spectrometer (OCEAN-FX-VIS-NIR-ES; Ocean Optics).

For these broadband SHG experiments, the illumination patterns on the waveguide were uniform in the transverse direction, whereas nontrivial QPM grating patterns were engineered in the longitudinal direction. In other words, the two-dimensional illumination pattern $I(x, z)$ had the form $I(x, z) = I(z)$. The simplest pattern we considered was a monotonic grating pattern

$$I_{\text{monotonic}}(z) = \text{H}\left(\sin(2\pi z/\Lambda)\right), \tag{S60}$$

where Λ is the spatial period and $H(x)$ is the Heaviside step function. Here, I was discretized in space and became the direct grayscale input to the SLM in the projector setup (see Sec. S7 A). The resulting QPM grating phase matched the SHG for a particular wavelength, yielding a solitary peak in the output SH spectrum, as shown in Fig. 3(a-i) for $\Lambda = 16.64\,\mu\text{m}$.

To perform multiple SHG processes simultaneously, we superimposed gratings with various periods. The complete grating structure was given by

$$I_{\text{multi-peaks}}(z) = \mathcal{N} \sum_{j=1} c_j \,\text{H}\left(\sin(2\pi z/\Lambda_j)\right) + \mathcal{C}, \tag{S61}$$

where c_j denotes the relative weights. In Fig. 3(a-ii), we show the results for $\Lambda_1 = 16.36\,\mu\text{m}$, $\Lambda_2 = 16.53\,\mu\text{m}$, $\Lambda_3 = 16.72\,\mu\text{m}$, and $\Lambda_4 = 16.88\,\mu\text{m}$, with corresponding weights $c_1 = 0.4$, $c_2 = 0.17$, $c_3 = 0.12$, and $c_4 = 0.15$. The weights were chosen so that the heights of the peaks in the spectrum were similar. The normalization constant $\mathcal{N}$ and the constant $\mathcal{C}$ were set so that $\min_z I(z) = 0$ and $\max_z I(z) = 1$.

We can also achieve simultaneous phase matching for broadband SHG by adiabatically chirping the QPM grating. To generate such an adiabatic AFC, as shown in Fig. 3(a-iii), we varied the grating period adiabatically from $\Lambda_{\text{ini}} = 16.3\,\mu\text{m}$ to $\Lambda_{\text{fin}} = 16.9\,\mu\text{m}$. Quantitatively, the QPM grating structure was given by

$$I_{\text{adiabatic}}(z) = \text{H}\Big(\sin\big(\theta_{\text{adiabatic}}(z)\, z\big)\Big). \tag{S62}$$

The accumulated grating phase was defined as

$$\theta_{\text{adiabatic}}(z) = \int_0^{L_{\text{img}}} \mathrm{d}z'\, k_{\text{adiabatic}}(z'), \tag{S63}$$

and the local wavenumber of the grating was expressed by

$$k_{\text{adiabatic}}(z) = \frac{2\pi}{\Lambda_{\text{ini}}} \frac{L_{\text{img}} - z}{L_{\text{img}}} + \frac{2\pi}{\Lambda_{\text{fin}}} \frac{z}{L_{\text{img}}}. \tag{S64}$$

Here, L_{img} denotes the total distance in the longitudinal dimension over which the programming illumination was projected. As shown in Fig. 3(b-iii), the resulting SHG spectrum was extremely broad, spanning over 50 nm in bandwidth. The full illumination patterns used in these experiments are shown in Fig. S26.

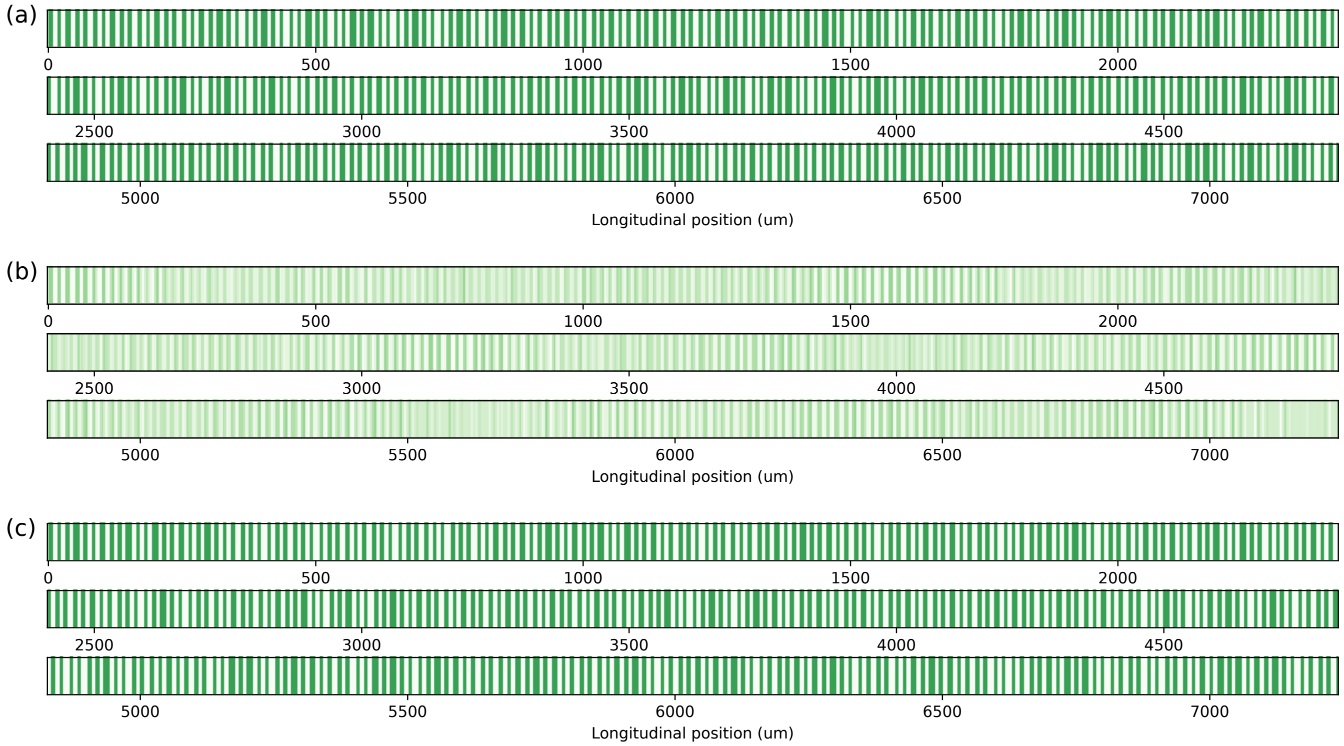

FIG. S26: The final illumination patterns $I(z)$ obtained through the optimization process. (a), (b), and (c) indicate Fig. 3(a-i), 3(a-ii), and 3(a-iii), respectively.

B. In situ optimization of the QPM grating

A key capability of our programmable nonlinear waveguide is its ability to modify the QPM grating structure in real time, which allowed us to optimize the grating on the basis of real-time experimental feedback. For example, in Fig. 3(b), we show how optimizing the QPM grating could shape the SH spectrum into the desired form. Below, we describe the experimental procedure used to achieve these results.

To enable flexible optimization of the QPM grating structure, we parameterized the illumination pattern $I(z)$ using several free parameters. First, we partitioned the entire imaging window of length L_{img} into $N_{\mathrm{opt}} = 20$ sections of equal length, $\Delta z_{\mathrm{opt}} = L_{\mathrm{img}}/N_{\mathrm{opt}}$. The overall illumination pattern was defined as

$$I(z) = \frac{c_{\mathrm{opt}}(z)}{2}\left\{\sin\bigl(\theta_{\mathrm{opt}}(z)z\bigr) + 1\right\}, \tag{S65}$$

where $c_{\mathrm{opt}}(z) = c_j$ for $(j-1)\Delta z_{\mathrm{opt}} \leq z < j\Delta z_{\mathrm{opt}}$, meaning that $c_{\mathrm{opt}}(z)$ takes the value c_j in the jth section. The overall phase function $\theta_{\mathrm{opt}}(z)$ was defined as

$$\theta_{\mathrm{opt}}(z) = \int_0^z \mathrm{d}z'\, k_{\mathrm{opt}}(z'), \tag{S66}$$

where $k_{\mathrm{opt}}(z)$ is the local wavenumber of the QPM grating. We parameterized $k_{\mathrm{opt}}(z)$ so that it increased linearly within each section, where the rate of increase depended on the section. In other words,

$$k_{\mathrm{opt}}(z) = \int_0^z \mathrm{d}z'\, \mu_{\mathrm{opt}}(z) + \frac{2\pi}{\Lambda_{\mathrm{ini}}}, \tag{S67}$$

where $\mu_{\mathrm{opt}}(z) = \mu_j \geq 0$ for $(j-1)\Delta z_{\mathrm{opt}} \leq z < j\Delta z_{\mathrm{opt}}$. We set the initial poling period to $\Lambda_{\mathrm{ini}} = 16.42\,\mu\mathrm{m}$. Overall, we obtained a monotonically chirped adiabatic QPM grating that was parameterized by $2N_{\mathrm{opt}}$ parameters, i.e., $\{c_j\}$ and $\{\mu_j\}$ with $j \in \{1, 2, \ldots, N_{\mathrm{opt}}\}$.

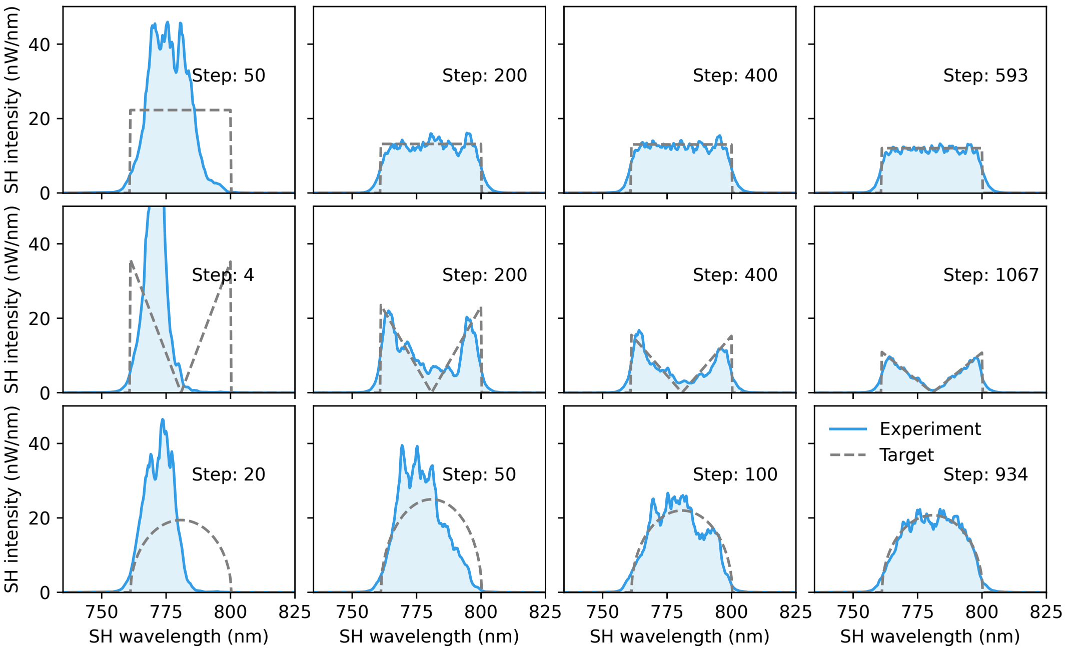

FIG. S27: Broadband SH spectra (blue shaded regions) that were experimentally measured during in situ optimization. The gray dashed lines represent the target spectra, which were normalized with respect to their peak values, and the experimental SH spectra were normalized so that the area under each curve matched that of the respective target spectrum. The first, second, and last rows correspond to the optimization steps for the data shown in Fig. 3(b-i), Fig. 3(b-ii), and Fig. 3(b-iii), respectively. The optimization step counts are indicated as integer numbers on the plots.

At each optimization step, we first measured the SH spectrum, $S_{\text{measured}}(\lambda)$, as a function of the wavelength λ. We then computed the normalized distance between $S_{\text{measured}}(\lambda)$ and the target spectrum $S_{\text{target}}(\lambda)$:

$$\mathcal{D} = \frac{\left(\int \mathrm{d}\lambda \left|S_{\text{measured}} - S_{\text{target}}\right|^2\right)^{1/2}}{\left(\int \mathrm{d}\lambda\, S_{\text{measured}}\right)\left(\int \mathrm{d}\lambda\, S_{\text{target}}\right)}. \tag{S68}$$

Next, we proposed an update to the QPM grating by applying a small perturbation to the parameters $\{c_j\}$ and $\{\mu_j\}$. If the perturbation decreased $\mathcal{D}$, we accepted the update; otherwise, we rejected it and proceeded to the next step. After many iterations, $S_{\text{measured}}(\lambda)$ was expected to converge toward the target spectrum. We used the bias voltage of $V_{\text{tot}} = 800\,\text{V}$ for this experiment. In Fig. S27, we show the evolution of the measured SH spectrum over different numbers of optimization steps. Although some target spectra were more challenging to achieve and required additional iterations, the output SHG spectrum eventually converged close to the target shape. In Fig. S28, we present the full QPM patterns $I(z)$ obtained from the optimization. For clarity in the main text, we downsample $I(z)$ every $17\,\mu\text{m}$ to provide a concise visualization of the QPM grating structure.

C. Real-time update of the QPM grating

In Fig. 3(d), we updated the programming illumination pattern $I(x, z)$ in real time, effectively projecting a "movie" onto the surface of a programmable nonlinear waveguide to achieve dynamic control of the broadband SHG spectrum. Although we show only approximately $\sim 300\,\text{s}$ of the trace in the main text, the operation was highly stable and could continue for much longer periods. In Fig. S29, we present a time trace of the SHG spectrum over 10 hours of operation, during which numerous "Cornell" patterns were generated. This process involved reconfiguring the $\chi^{(2)}$ nonlinearity 15,400 times, and we did not observe any practical upper bound on the cycle count. These results provide compelling visual evidence of the stability and repeatability of the programmable nonlinear waveguide. By comparing the SHG power from the same QPM pattern over the course of the measurement, we observed approximately 15 % drift in power. Although such fluctuations may be caused by misalignment of the pump beam from the fundamental mode

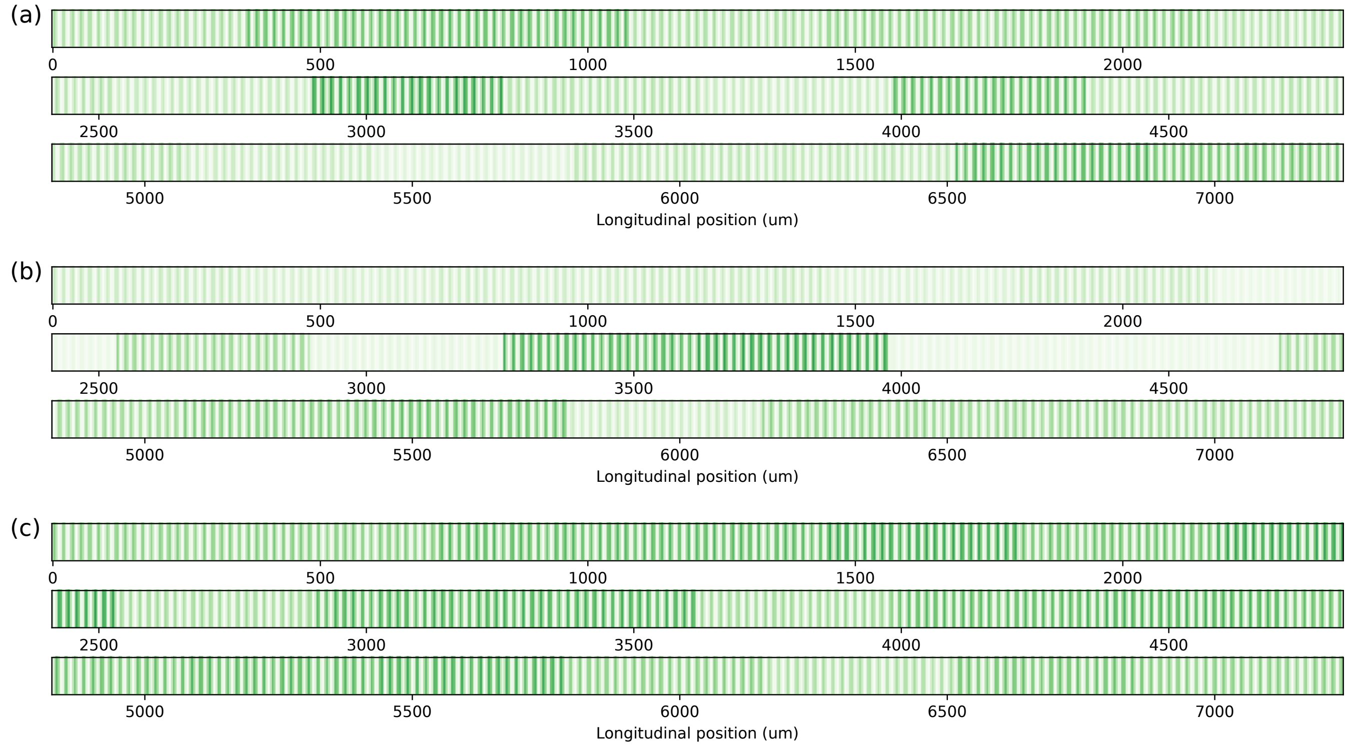

FIG. S28: The final illumination patterns $I(z)$ obtained through the optimization process. (a), (b), and (c) indicate Fig. 3(b-i), 3(b-ii), and 3(b-iii), respectively.

of the waveguide—e.g., due to typical temperature fluctuations between 21 °C and 23 °C in the room or mechanical vibrations—it is challenging to accurately estimate their contribution due to the multimode nature of the waveguide. This measurement provides an upper bound on the instability of the $\chi^{(2)}$ nonlinearity, estimated to be approximately 7 % over 10 hours under 2 °C degrees of temperature variation.

The pattern we could obtain in the trace of the SH spectrum was not limited to the one shown in the main text. In Fig. S30, we give another demonstration, drawing an “NTT” pattern. We used the bias voltage of $V_{\mathrm{tot}} = 800\,\mathrm{V}$ for the experiments presented in this section.

S10. SPATIAL ENGINEERING

In this section, we present the experimental details of the results on spatial engineering shown in the main text. Specifically, we explain how the experimental data and simulation results presented in Fig. 4 were experimentally produced. Throughout the section, we use the notation introduced in Sec. S6.

A. Calibration of the experimental setup

In Fig. S31, we illustrate the experimental setup. Pulse pump light was coupled to the programmable nonlinear waveguide, and the spatial profile of the generated SH light was imaged with a camera. We used the bias voltage of $V_{\mathrm{tot}} = 600\,\mathrm{V}$ for the experiments presented in this section. First, we calibrated the magnification ratio of this imaging system that measured the SH beam profile. This calibration was performed by translating the programmable waveguide by a known distance via a micrometer stage, capturing images of the output facet, and measuring the displacement of the visible features in the camera image. Overall, we obtained a magnification ratio of 8.67, meaning that the output SH light was magnified by a factor of 8.67 when it reached the camera.

To achieve accurate control of the spatial profiles of SHG, the spatial profile of the pump light on the waveguide should be known. This is nontrivial in a conventional waveguide because one cannot simply “cut open” the waveguide to measure the field profile. Fortunately, the programmability of our platform offered a unique solution to this

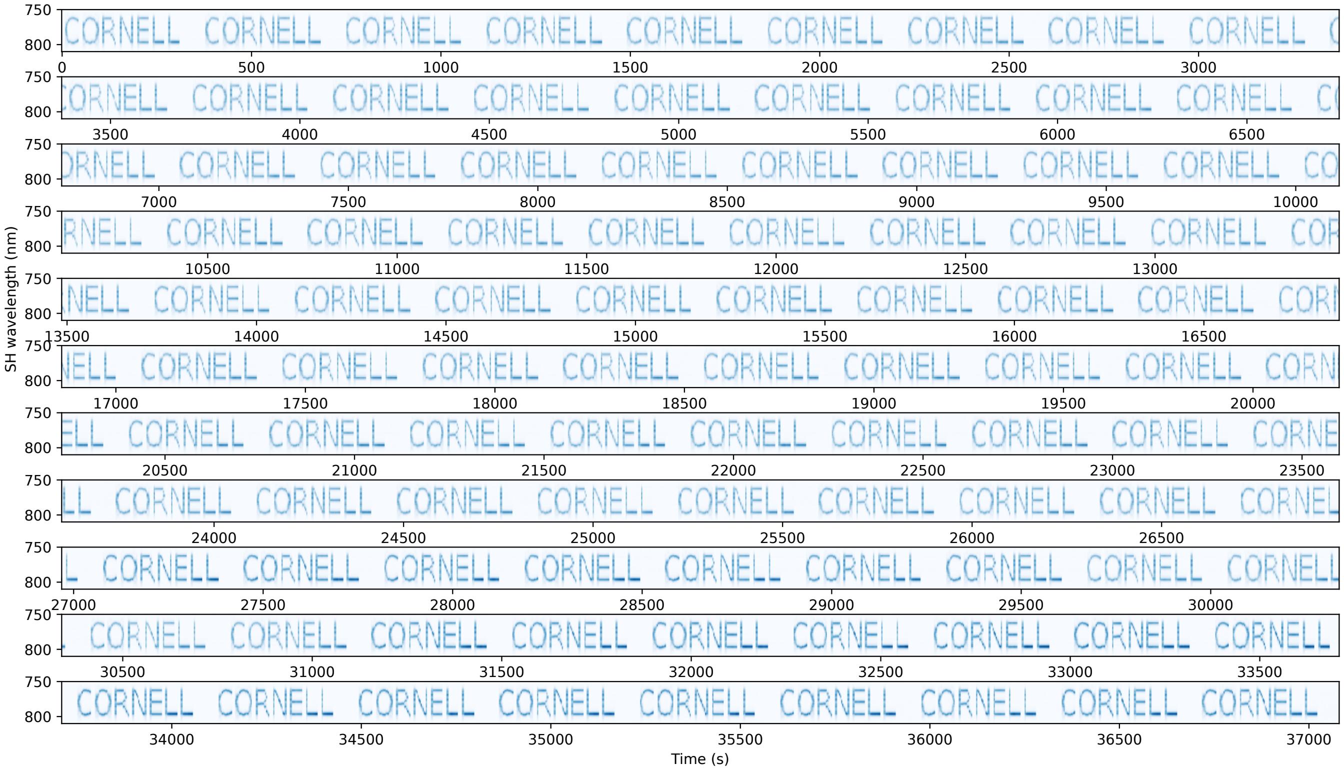

FIG. S29: Time trace of the SH spectrum for over 10 hours of operation, involving 15400 updates to the QPM grating patterns. The illumination patterns were optimized beforehand to produce the "Cornell" pattern in the SH spectrum, which we repeated multiple times.

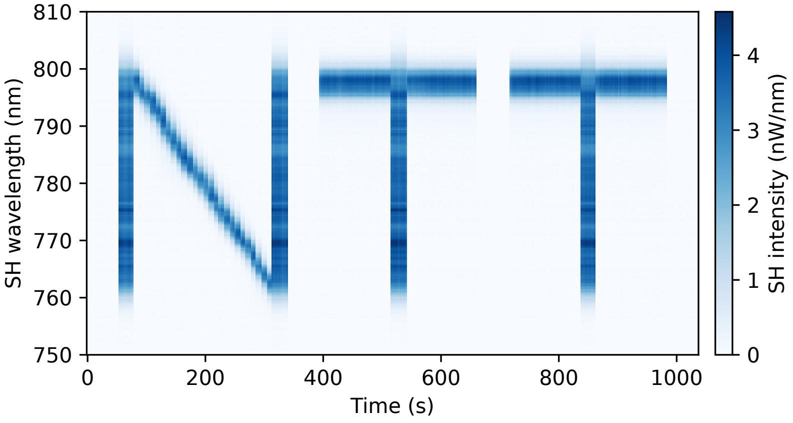

FIG. S30: Time trace of the SH spectrum showing an "NTT" pattern. We used the same experimental procedure as in Fig. S29.

challenge.

Our approach for measuring the pump light intensity distribution, $|a|^2(x, z)$, is illustrated in Fig. S32. In this measurement, we projected a QPM grating pattern onto the programmable waveguide, but only within a small rectangular region of interest (ROI) centered at the position (x', z'). If the ROI does not overlap with the pump light's intensity distribution, no SH light is detected because the SHG process is not phase matched without a QPM grating (see Fig. S32(a)). Conversely, when the ROI overlaps with the pump light, as shown in Fig. S32(b), the SHG becomes phase matched, and SH light is generated. Since the SHG power is proportional to the square of the pump intensity in the ROI, i.e., $P_{\mathrm{SH}} \propto |a|^4(x', z')$, scanning the ROI across the programmable region allowed us to

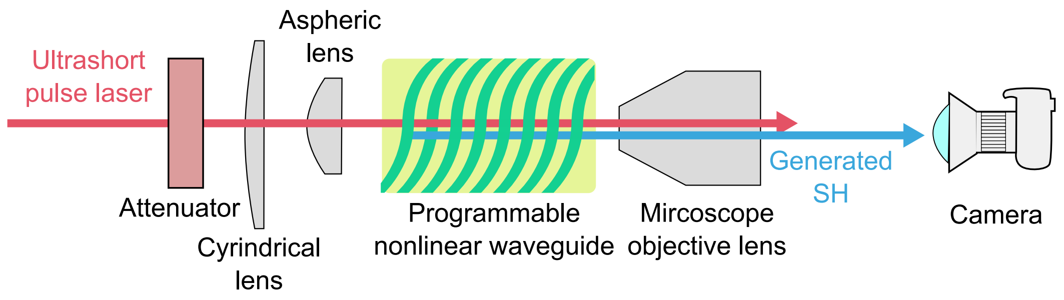

FIG. S31: Experimental setup for spatial-domain control of SHG. The optics on the input side were identical to those in Fig. S25. On the output side, we used a microscope objective lens (PLN 10X Objective; Olympus) to image the transverse spatial profiles of the generated SH light with a camera (acA1440-220um; Basler).

experimentally map out the spatial profile of $|a|^2$.

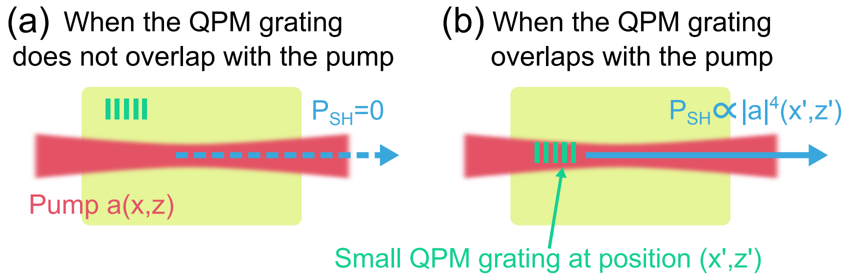

FIG. S32: A method of measuring the spatial distribution of the pump intensity $|a|^2(x, z)$ on the waveguide using the programmability of the $\chi^{(2)}$ nonlinearity. (a) As we project the QPM grating pattern only within a small ROI, we observe no SHG when the ROI does not overlap with the pump light. (b) When the ROI overlaps with the pump light, we observe the SHG, whose power is proportional to the square of the local pump intensity around the ROI.

In Fig. S33(a), we show the results of this measurement, where the experimentally measured pump-intensity distribution $|a|^2$ is presented in SLM coordinates. On the basis of this map, we defined a waveguide coordinate system—a Cartesian coordinate system aligned with the pump beam. When the pump beam was well aligned, the SLM and waveguide coordinates approximately coincided; however, small corrections were usually necessary for accurate system calibration. In Fig. S33(a), the solid cyan line represents the centroid of the pump beam, which was tilted by 0.007 rad relative to the SLM coordinate. We designated this line as the z-axis of the longitudinal waveguide coordinate, which in turn defined the origin for the transverse waveguide coordinate. The origin of the longitudinal coordinate was set at the beginning of the region in which the programming illumination was projected. The dashed cyan line indicates the approximate longitudinal position where the SHG intensity was strongest, $z = z_{\mathrm{ref}} = 3772\,\mu\mathrm{m}$, which we used as a reference point in the following.

To quantitatively parameterize the beam profiles, we approximated the pump field at the reference point as a Gaussian beam:

$$a(x, z = z_{\mathrm{ref}}) \propto e^{iu_{\mathrm{ref}}x^2/2}\, e^{-x^2/w^2_{\text{FH-ref}}}. \tag{S69}$$

This was a reasonable assumption since the pump beam was coupled from a single-mode fiber, which cleans up its spatial profile. In Fig. S33(b), we show the cross-section of the FH intensity at $z = z_{\mathrm{ref}}$, along with a Gaussian fit, $|a|^2 \propto \exp(-2x^2/w^2_{\mathrm{FH}})$, on the basis of Eq. (S69). The beam waist at the reference position was found to be $w_{\text{FH-ref}} = 132\,\mu\mathrm{m}$. The SH field generated from a thin slice around $z = z_{\mathrm{ref}}$ was given by

$$b(x, z = z_{\mathrm{ref}}) \propto a^2(x, z = z_{\mathrm{ref}}) \propto e^{iu_{\mathrm{ref}}x^2}\, e^{-x^2/w^2_{\text{SH-ref}}}, \tag{S70}$$

with a beam waist of $w_{\text{SH-ref}} = w_{\text{FH-ref}}/\sqrt{2} = 93.3\,\mu\mathrm{m}$.

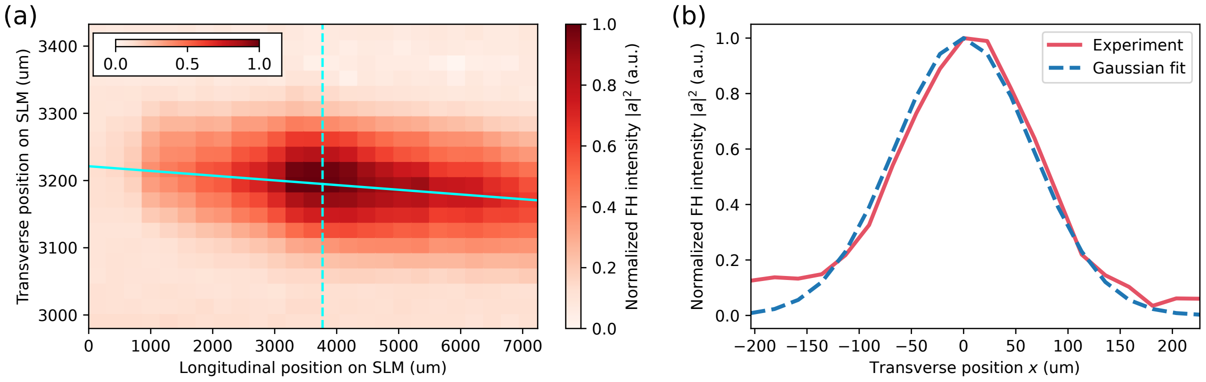

FIG. S33: (a) Distribution of the intensity of the pump light mapped using the technique shown in Fig. S32. The coordinates were defined according to the axis of the SLM. The solid cyan line represents the approximate center of the pump beam, which defines the z-axis for the longitudinal coordinate with $x = 0$. The dashed cyan line represents the longitudinal position $z = z_0 = 3772\,\mu\text{m}$ around which the SHG was strongest. (b) Cross section of the FH beam profile at $z = z_0$, shown with a Gaussian fit.

To design accurate QPM gratings for engineering the spatial profiles of SHGs, it was essential to characterize the overall geometry of the waveguide precisely. In particular, we needed to determine the position of the output facet, $z = L_{\text{out}}$, relative to the defined coordinates. For this purpose, we employed an approach based on beam steering of the SHG. Specifically, we projected a flat QPM grating with a tilt angle of θ_{tilt} relative to the waveguide coordinate axes and measured the displacement observed in the camera image of the output SH profile. Intuitively, a larger L_{out} produced a larger displacement. In Fig. S34(a), we show the output beam profiles for various values of θ_{tilt}. The origin $x = 0$ in the camera image was set so that the output beam was approximately centered when $\theta_{\text{tilt}} = 0$. The measured displacement, d_{disp}, was fitted to the following function:

$$d_{\text{disp}} = -\frac{2\pi \tan\theta_{\text{tilt}}}{k_2 \Lambda}(L_{\text{out}} - z_0), \tag{S71}$$

where k_2 is the wavenumber for the SH and the QPM grating period $\Lambda = 16.75\,\mu\text{m}$ was chosen to phase match the SHG at an output wavelength of 790 nm. The fitting process yielded $L_{\text{out}} = 1.58 \times 10^4\,\mu\text{m}$.

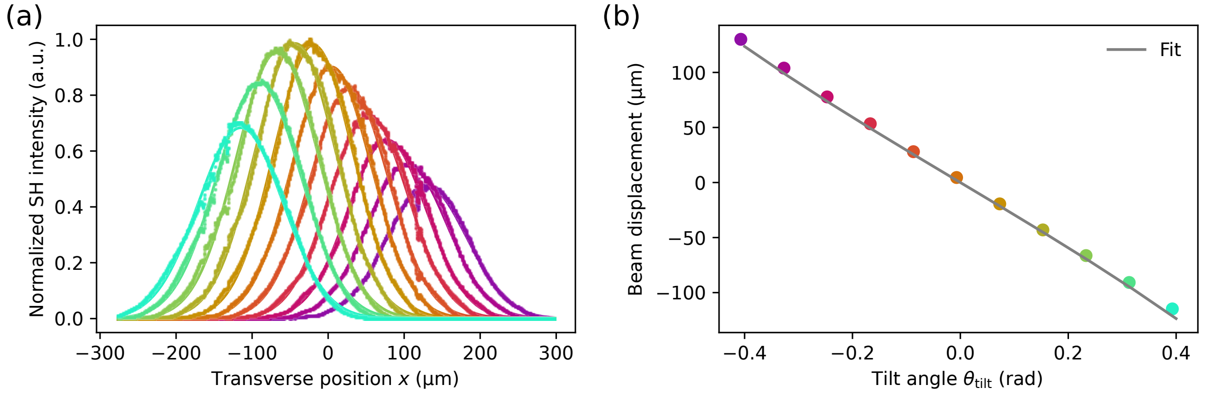

FIG. S34: Beam-steering technique used to characterize the beam propagation distance. (a) Circles: Experimentally measured transverse beam profile of the SHG for various tilt angles of the QPM grating with period $\Lambda = 16.75\,\mu\text{m}$. Solid lines: Gaussian fits of the spatial profiles. (b) Circles: The location of the beam centroid based on Gaussian fits for various tilt angles. Solid line: A least-squares fit based on Eq. (S71), yielding $L_{\text{out}} = 1.58 \times 10^4\,\mu\text{m}$. The colors of the markers in (b) provide the legend for the values of θ_{tilt} in (a).

The final unknown parameter needed for a full characterization of the experimental setup was the transverse spatial chirp of the field, u_{ref}. Because this parameter affected only the phase of the electric field, it was difficult to extract it from an intensity distribution (e.g., Fig. S33), particularly when the Rayleigh length was large. To determine u_{ref},

we again exploited the programmability of our nonlinear waveguide. Specifically, we projected a QPM grating with quadratic curvature, parameterized as

$$I_{\text{quad}}(x,z) = \frac{1}{2}\left\{\sin\left(2\pi z\Lambda^{-1} - q_{\text{quad}}\,x^2\right) + 1\right\}, \tag{S72}$$

onto a thin slice of the region around the reference point, defined by $z_{\text{ref}} - \epsilon_z/2 \le z \le z_{\text{ref}} + \epsilon_z/2$. The generated SH profile then inherited a spatial chirp and became

$$b_{\text{SH-quad}}(x, z = z_{\text{ref}}) \propto e^{-iq_{\text{quad}}\,x^2} a^2(x, z = z_{\text{ref}}) \propto e^{i(-q_{\text{quad}}+u_{\text{ref}})x^2} e^{-x^2/w^2_{\text{SH-ref}}}. \tag{S73}$$

Intuitively, the spatial chirp term acted like a lens applied to a Gaussian beam, where q_{quad} controlled the effective curvature of the lens. Thus, tuning q_{quad} allowed us to control the focus of the SH beam.

In Fig. S35, we show experimentally measured output SH beam profiles for various q_{quad} values. Specifically, we determined an optimal value $q_{\text{quad}} = q_{\text{opt}}$ that minimized the width of the SH beam at the output facet, $z = L_{\text{out}}$, resulting in a minimum beam waist of $w_{\text{SH-opt}} = 16.4\,\mu\text{m}$ at the output facet. We assumed that the beam was spatially unchirped at this point, which allowed us to calculate the Rayleigh range of the SH as $z_{\text{SH-R}} = 2072\,\mu\text{m}$. Next, we backtracked the spatial evolution of the beam profile from the output facet to the reference point, yielding

$$b_{\text{SH-opt}}(x, z = z_{\text{ref}}) \propto e^{i\frac{k_2 x^2}{2\tilde{R}_{\text{SH-opt}}}} e^{-x^2/\tilde{w}^2_{\text{SH-opt}}}, \tag{S74}$$

with

$$\tilde{R}_{\text{SH-opt}} = (z_{\text{ref}} - L_{\text{out}})\left[1 + \frac{z^2_{\text{SH-R}}}{(z_{\text{ref}} - L_{\text{out}})^2}\right], \tag{S75}$$

$$\tilde{w}^2_{\text{SH-opt}} = w^2_{\text{SH-opt}}\left(1 + \frac{(z_{\text{ref}} - L_{\text{out}})^2}{z^2_{\text{R-opt}}}\right). \tag{S76}$$

Equations (S73) and (S74) should be equivalent when $q_{\text{quad}} = q_{\text{opt}}$. Solving the equality $\tilde{w}_{\text{SH-opt}} = w_{\text{SH-ref}}$ for L_{out} yielded $L_{\text{out}} = 1.54 \times 10^4\,\mu\text{m}$, which reasonably agreed with the value estimated via the beam-steering method, $L_{\text{out}} = 1.58 \times 10^4\,\mu\text{m}$. Owing to the sparsity of the data in Fig. S35, the beam-focusing approach was expected to be less accurate; therefore, we adopted the beam-steering-based estimate of L_{out} as our best estimate. Finally, by equating the spatial chirp values, we determined $u_{\text{ref}} = 1.23 \times 10^{-4}\,\text{rad}/\mu\text{m}^2$.

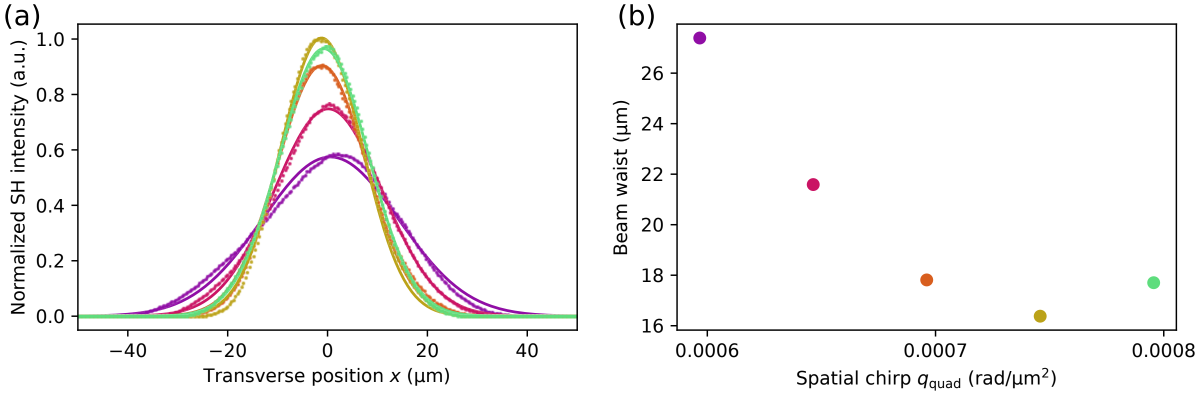

FIG. S35: (a) Circles: Experimentally measured spatial profile of the generated SH with a quadratically chirped QPM grating Eq. (S72) with $\epsilon = 377.2\,\mu\text{m}$ and various values of q_{quad}. Solid lines: Gaussian fits of the spatial beam profile. (b) Beam waists of the SH beams obtained by Gaussian fitting for various chirps q_{quad}. The colors of the markers provide the legend for q_{quad} in (a).

B. Model for spatially engineered SHG

In this section, we establish a numerical model for spatially engineered SHG in a programmable nonlinear waveguide using the parameters calibrated in the previous section. To simplify the analysis, we make the following assumptions:

(i) the pump field remained undepleted during propagation; (ii) the pump field was monochromatic CW light with a wavelength of 1580 nm, generating SHG at 790 nm; (iii) the optical loss in the waveguide was negligible; and (iv) the induced $\chi^{(2)}$ nonlinearity was proportional to the intensity of the programming illumination, $I(x, z)$.

We take the models derived in Sec. S6 as our starting point. The FH field profile at the reference position $z = z_{\text{ref}}$ is given by

$$a(z_{\text{ref}}, x) = a_0\, e^{iu_{\text{ref}}x^2/2}\, e^{-x^2/w^2_{\text{FH-ref}}}, \tag{S77}$$

where the parameters u_{ref} and $w_{\text{FH-ref}} = \sqrt{2}\, w_{\text{SH-ref}}$ are determined in Sec. S10 A. Under assumptions (i), (ii), and (iii), we can solve Eq. (S48) to obtain

$$a(z, x) = e^{\frac{i}{2k_1}(z-z_{\text{ref}})\partial_x^2}\, a(z_{\text{ref}}, x). \tag{S78}$$

Assumption (iv) implies that

$$r(x, z) = C\, I(x, z), \tag{S79}$$

where $r(x, z)$ represents the spatial distribution of the $\chi^{(2)}$ nonlinearity and C is a constant. Since only the Fourier component of $I(x, z)$ near the spatial frequency $2\pi/\Delta k$ significantly contributes to the SHG dynamics, we modify Eq. (S49) to obtain

$$\partial_z b(x, z) \approx \frac{i}{2k_2}\partial_x^2 b(x, z) - i\, C\, \kappa\, \mathcal{I}(x, z)\, a^2(x, z), \tag{S80}$$

where the complex illumination pattern $\mathcal{I}$ is defined via

$$I(x, z) = e^{i\Delta k\, z}\, \mathcal{I}(x, z) + e^{-i\Delta k\, z}\, \mathcal{I}^*(x, z). \tag{S81}$$

We numerically obtained the SH field profile, up to an overall scaling factor C, by integrating Eq. (S80) with the initial condition $b(x, z) = 0$ and using the FH field profile from Eq. (S78). The output SH beam measured by the camera is proportional to $|b(x, z = L_{\text{out}})|^2$.

C. Experimental results and simulations

In this section, we use the calibration results from Sec. S10 A to design QPM grating structures for spatially engineering the SHG and compare them with numerical simulations based on the model developed in Sec. S10 B. In particular, we describe how the results in Fig. 4 in the main text were obtained.

For Fig. 4(b), we projected a monotonic QPM grating with period $\Lambda = 16.75\,\mu\text{m}$, in which $\Delta k = 2\pi/\Lambda$ was set. We used this parameter for the remaining experiments as well. The beam propagation simulation was performed with $\mathcal{I}(x, z) = 1$. Note that the induced $\chi^{(2)}$ nonlinearity was assumed to be zero for $z > L_{\text{img}}$ (i.e., outside the region of patterned illumination). The overall scaling and phase of $\mathcal{I}(x, z)$ do not affect the result.

For Fig. 4(c), we implemented a quadratically chirped grating. The base programming illumination pattern was given by

$$I_{\text{focus}}(x, z) = \frac{1}{2}\Big\{\sin\Big(2\pi z/\Lambda - q_{\text{focus}}(z)x^2\Big) + 1\Big\}, \tag{S82}$$

where the chirp parameter was defined as

$$q_{\text{focus}}(z) = q_{\text{opt}} + q_{\text{chirp}}(z - z_{\text{ref}}). \tag{S83}$$

The projected pattern involved appropriate normalizations and offsets. The resulting nonlinearity pattern became

$$\mathcal{I}_{\text{focus}}(x, z) \propto e^{-iq_{\text{focus}}(z)x^2}. \tag{S84}$$

The linear chirp of the curvature, q_{chirp}, compensated for the variation in the optimal transverse chirp at different longitudinal positions. We used $q_{\text{chirp}} = 5.3 \times 10^{-7}\,\text{rad}/\mu\text{m}^3$.

In Fig. 4(d), we superimposed multiple grating patterns, I_{focus}, with different spatial offsets to produce a comb-like structure with n_{comb} peaks. For this purpose, we projected an illumination pattern given by

$$I_{\text{comb}}(x, z) = \mathcal{N}\sum_{j=1}^{n_{\text{comb}}} I_{\text{focus}}\big(x - x_j, z\big) + \mathcal{C}, \tag{S85}$$

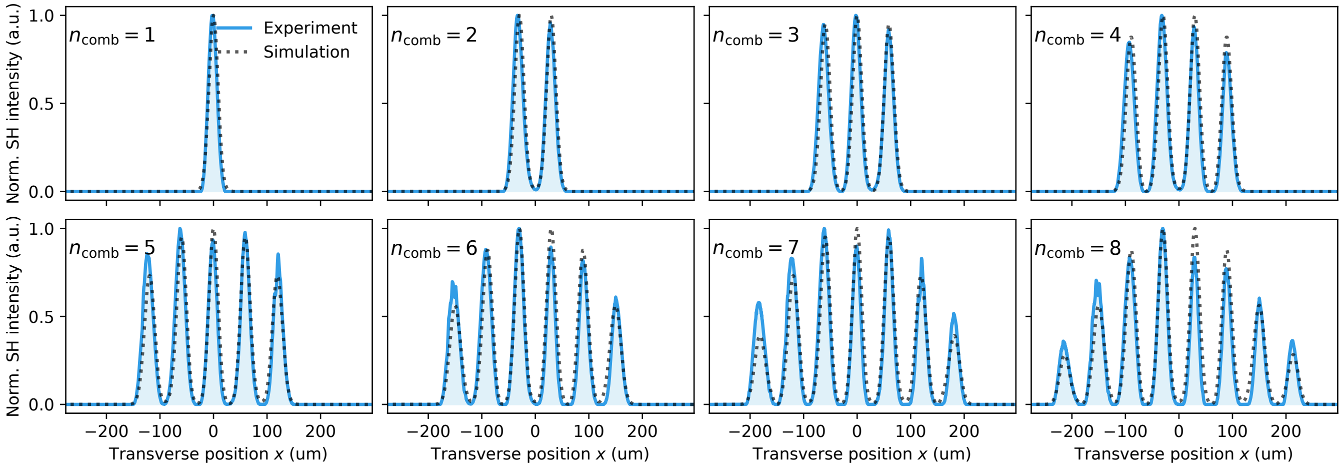

FIG. S36: Solid lines: Experimentally measured spatial SH beam profiles for the illumination patterns of Eq. (S85) for various numbers of peaks n_{comb} with $\Delta x = 52.8\,\mu\mathrm{m}$. Dotted lines: Results of the numerical simulations with no free parameter except for the overall scaling, which was set so that the peak values are unity.

where $x_j = \Delta x \left(\frac{n_{\mathrm{comb}}-1}{2} + j - 1\right)$ is the offset for the jth pattern. The constants $\mathcal{N}$ and $\mathcal{C}$ were chosen so that the dynamic range of the illumination was unity. The overall distribution of the $\chi^{(2)}$ nonlinearity used in the simulation was

$$\mathcal{I}_{\mathrm{comb}}(x,z) \propto \sum_{j=1}^{n_{\mathrm{comb}}} e^{-iq_{\mathrm{focus}}(z)(x-x_j)^2}. \tag{S86}$$

In addition to the case of $n_{\mathrm{comb}} = 9$ shown in the main text, we present results for different values of n_{comb} in Fig. S36, which uniformly demonstrate good agreement between theory and experiment.

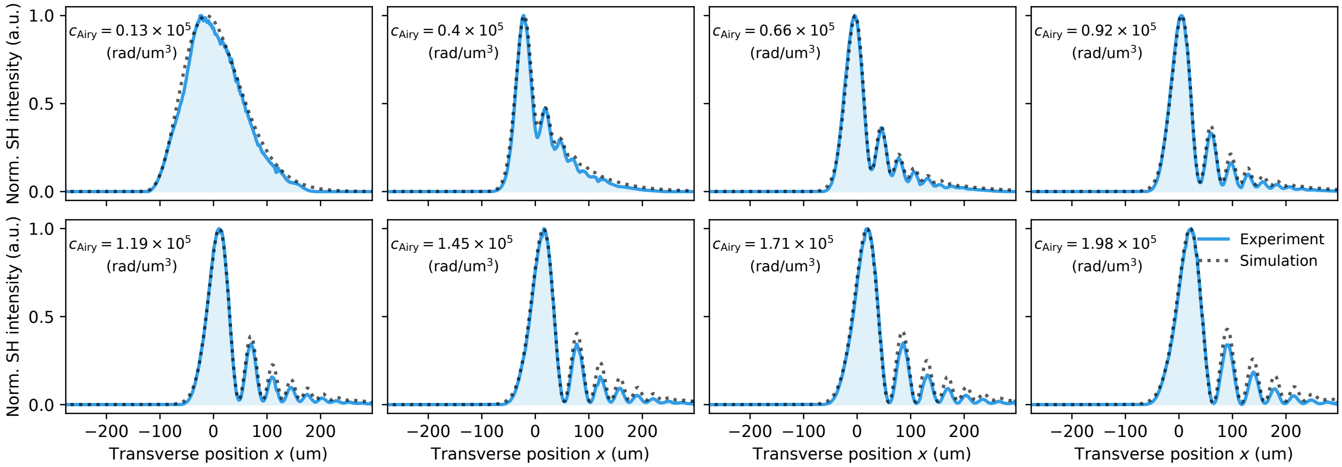

FIG. S37: Solid lines: Experimentally measured spatial SH beam profiles for the illumination patterns of Eq. (S87) for various values of c_{Airy}. Dotted lines: Results of the numerical simulations with no free parameter except for the overall scaling, which was set so that the peak values were unity.

Finally, in Fig. 4(d), we demonstrated the generation of an Airy beam via a cubically chirped QPM grating. Specifically, we used

$$I_{\mathrm{Airy}}(x,z) = \frac{1}{2}\left(\sin\left(2\pi z/\Lambda + c_{\mathrm{Airy}}x^3\right) + 1\right), \tag{S87}$$

which was projected onto a thin slice defined by $z_{\rm ref} - \epsilon_z/2 \leq z \leq z_{\rm ref} + \epsilon_z/2$, where $\epsilon_z = 7242\,\mu$m. For the simulation, we used

$$\mathcal{I}_{\rm Airy}(x, z) \propto e^{i c_{\rm Airy} x^3}. \tag{S88}$$

In the main text, we present the experimental results for $c_{\rm Airy} = 1.05 \times 10^{-5}\,{\rm rad}/\mu{\rm m}^3$, and in Fig. S37, we present the results for additional values of $c_{\rm Airy}$.

S11. SPATIO-SPECTRAL ENGINEERING

In this section, we describe how we obtained the results presented in the main text for the spatio-spectral engineering of the SHG. Figure S38 shows a photograph of the experimental setup used to acquire the data. The optics for the input and programming illumination, as well as the electronics, were the same as those described in Appendices S7 and S10; however, we extended the detection system to resolve both spatial and spectral features. We used the bias voltage of $V_{\rm tot} = 600$ V for the experiments presented in this section.

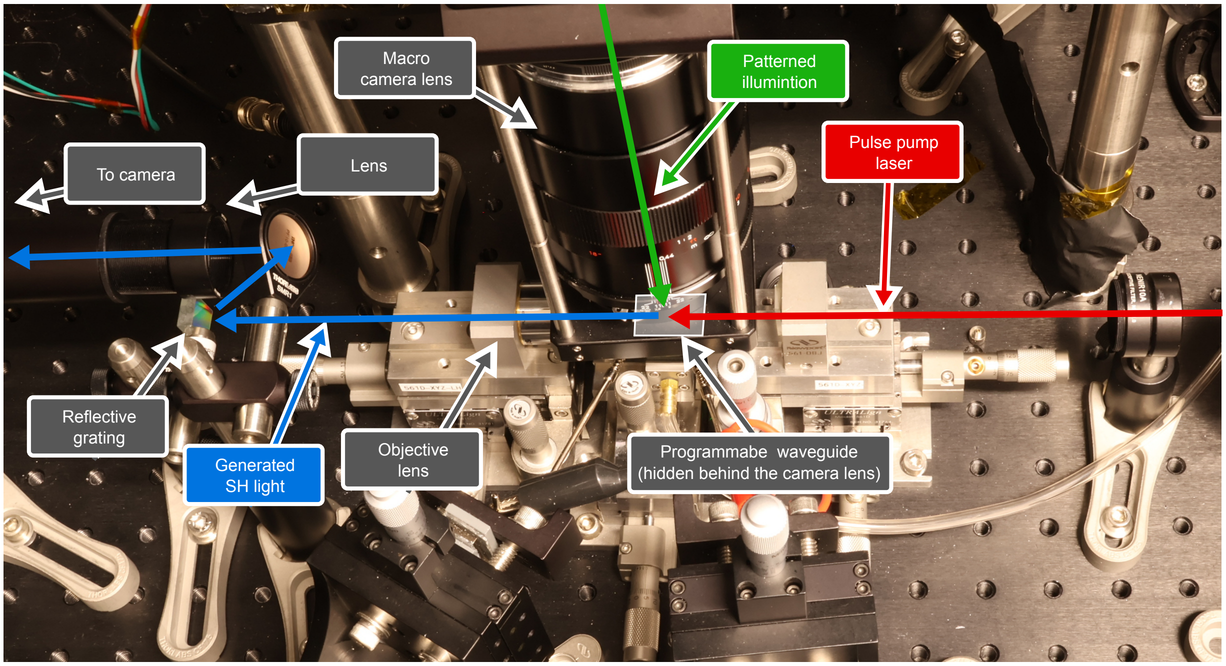

FIG. S38: Photograph of the experimental setup used to perform spectrally resolved imaging of the broadband SH output. The setup produced the data for the main text.

In our setup, the objective lens of a microscope was placed one focal distance from the output facet of the programmable waveguide. The beam then impinged on a reflective grating that diffracts the light in the vertical (i.e., y) direction, where the diffraction angle depended on the wavelength. A second lens, positioned one focal distance from the grating, focused the output onto a camera for detection. Consequently, the horizontal axis of the camera image resolved the spatial features of the output light, whereas the vertical axis resolved the spectral features.

We calibrated the setup for both the spatial and spectral domains. To characterize the spatial magnification of the imaging system, we followed the procedure described in Sec. S10. Calibrating the spectral coordinate—that is, determining which wavelength corresponds to each camera pixel—was more involved. First, we projected monotonic QPM grating patterns with period Λ and measured the generated SH wavelength with a spectrometer. This measured relationship between Λ and the SH wavelength established the mapping between the vertical coordinate of the camera sensor and the SH wavelength. We also calibrated the pump beam profile on the waveguide via the procedure outlined in Sec. S10.

For simultaneous engineering of spatial and spectral features, we exploited the full two-dimensional programmability of the waveguide. In Fig. 5(b), we superimposed multiple quadratically chirped grating patterns Eq. (S82) with

different spatial offsets and base periods. Specifically, we had

$$I_{\text{2D-comb}}(x,z) = \mathcal{N} \sum_{k=1}^{n_{\text{spec}}} \sum_{j=1}^{n_{\text{comb},k}} c_k \, I_{\text{focus}}(x - x_j, z, \Lambda_k) + \mathcal{C}, \tag{S89}$$

where n_{spec} is the number of spectral bands we addressed independently, $n_{\text{comb},k}$ is the number of comb lines in the kth spectral band, and Λ_k is the QPM grating period used to phase match the SHG for the kth band. In the main text, we used $n_{\text{spec}} = 5$, meaning that we independently controlled 5 different spectral bands. The wavelength of each band was determined by the QPM grating period, which we set as $\Lambda_1 = 16.4\,\mu\text{m}$, $\Lambda_2 = 16.5375\,\mu\text{m}$, $\Lambda_3 = 16.675\,\mu\text{m}$, $\Lambda_4 = 16.8125\,\mu\text{m}$, and $\Lambda_5 = 16.95\,\mu\text{m}$, with relative weights of $c_1 = 0.3$, $c_2 = 0.14$, $c_3 = 0.13$, $c_4 = 0.19$, and $c_5 = 0.45$. The number of peaks we engineer was given by $n_{\text{comb},k} = k$ for $k \in \{1,2,3,4,5\}$. The normalization constant $\mathcal{N}$ was set so that the maximum value of the grayscale illumination was 0.8. In Fig. S39, we display all fifteen base patterns that were summed to produce the full QPM grating pattern $I_{\text{2D-comb}}$. Finally, in Fig. S40, we present another example of spatio-spectral engineering, which indicated the formation of sixteen distinct peaks.

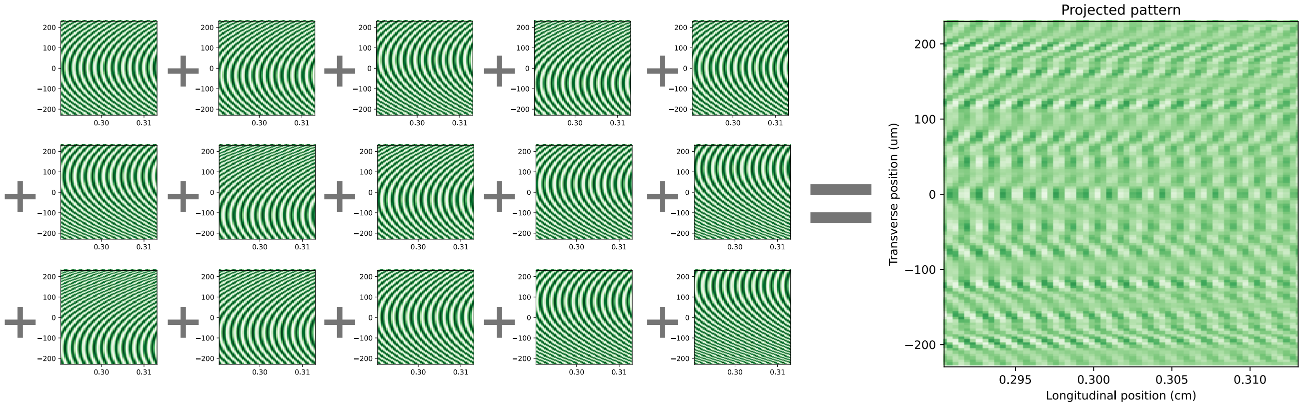

FIG. S39: The full illumination pattern Eq. (S89), composed of fifteen base patterns, is shown. Each pattern on the left focused a particular wavelength of the SH onto a particular position. Overall, the total pattern shown on the right focused the SH light into fifteen distinct peaks in the two-dimensional phase space spanned by the transverse position and the SH wavelength.

The spatio-spectral engineering achievable on a programmable nonlinear waveguide is not limited to comb-like structures. As shown in Fig. 5(c), we engineered Airy beams with different chirp parameters at different SH wavelengths. This was achieved by projecting the following programming illumination pattern:

$$I_{\text{2D-Airy}}(x,z) = \mathcal{N} \sum_{k=0}^{n_{\text{spec}}} c_k(z) I_{\text{Airy}}(x, z; \Lambda_k, c_{\text{Airy},k}) + \mathcal{C} \tag{S90}$$

with $n_{\text{spec}} = 2$, $\Lambda_1 = 16.4\,\mu\text{m}$, $\Lambda_2 = 16.95\,\mu\text{m}$, $c_{\text{Airy},1} = 1.85 \times 10^{-5}\,\text{rad}/\mu\text{m}^3$, and $c_{\text{Airy},2} = -1.32 \times 10^{-5}\,\text{rad}/\mu\text{m}^3$. Here, $c_1(z)$ took a value of 0.3 only when $1886\,\mu\text{m} \le z \le 3019\,\mu\text{m}$. Similarly, $c_2(z)$ took a value of 0.4 only for $3019\,\mu\text{m} \le z \le 3772\,\mu\text{m}$.

S12. NEED FOR EXPERIMENTAL CHARACTERIZATION OF THE ELECTRIC-FIELD-INDUCED $\chi^{(2)}$ NONLINEARITY

In this section, we describe the underlying physics of electric-field-induced $\chi^{(2)}$ nonlinearity, relating its value to a tensor element of the native $\chi^{(3)}$ nonlinearity. Our discussion suggests that identifying an ideal material for a programmable nonlinear waveguide (and for electric-field-induced $\chi^{(2)}$ nonlinearity in general) dedicated experimental efforts are required to directly measure the induced $\chi^{(2)}$ for each material rather than relying solely on tabulated values of the optical $\chi^{(3)}$ nonlinearity.

Below, we adopt the mathematical notation provided in Ref. [S68], which differs slightly from that used elsewhere in this manuscript. The electric-field-induced $\chi^{(2)}$ nonlinearity can be understood as follows. The third-order nonlinear

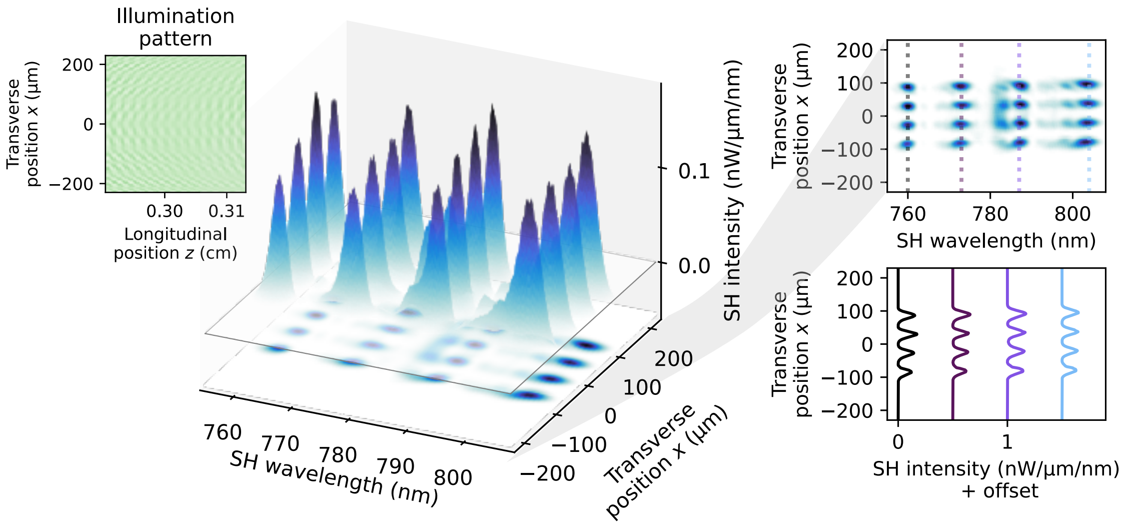

FIG. S40: Demonstration of the generation of sixteen-peak spatiotemporal features via SHG on a programmable nonlinear waveguide. The projected programming illumination was characterized by $n_{\text{spec}} = 4$, $\Lambda_1 = 16.4\,\mu\text{m}$, $\Lambda_2 = 16.583\,\mu\text{m}$, $\Lambda_3 = 16.767\,\mu\text{m}$, $\Lambda_4 = 16.4\,\mu\text{m}$, and $n_{\text{comb},k} = 4$. The weights were $c_1 = 0.3$, $c_2 = 0.15$, $c_3 = 0.12$, and $c_4 = 0.47$.

polarization of a material is given by

$$P_i^{(3)}(\omega_m + \omega_n + \omega_o) = \epsilon_0 \sum_{jkl} \sum_{(mno)} \chi^{(3)}_{ijkl}(\omega_m + \omega_n + \omega_o, \omega_m, \omega_n, \omega_o) E_j(\omega_m) E_k(\omega_n) E_l(\omega_o), \tag{S91}$$

where (mno) denotes all permutations of the indices. Electric-field-induced $\chi^{(2)}$ nonlinearity arises when one of the electric fields is replaced by a bias field E_{bias}, whose frequency is essentially zero compared with the optical frequency. Focusing on interactions among fields in the y direction, we obtain

$$\begin{aligned} P_y^{(2)}(\omega_m + \omega_n) &= 3\epsilon_0 \sum_{(mn)} \chi^{(3)}_{yyyy}(\omega_m + \omega_n, \omega_m, \omega_n, 0) E_y(\omega_m) E_y(\omega_n) E_{\text{bias}} \\ &= \epsilon_0 \sum_{(mn)} \chi^{(2)}_{yyy}(\omega_m + \omega_n, \omega_m, \omega_n) E_y(\omega_m) E_y(\omega_n). \end{aligned} \tag{S92}$$

Thus, the effective $\chi^{(2)}$ nonlinear tensor element is given by

$$\chi^{(2)}_{yyy}(\omega_m + \omega_n, \omega_m, \omega_n) = 3\chi^{(3)}_{yyyy}(\omega_m + \omega_n, \omega_m, \omega_n, 0) E_{\text{bias}}. \tag{S93}$$

To achieve better performance for a programmable nonlinear waveguide, it is therefore desirable to find a material with a large value of $\chi^{(3)}_{yyyy}(\omega_m + \omega_n, \omega_m, \omega_n, 0)$.

One might assume that optimal materials can be identified via tabulated values of the $\chi^{(3)}$ optical nonlinearity, such as the nonlinear index n_2. However, this approach is inadequate because the tabulated values refer to $\chi^{(3)}(\omega_m + \omega_n + \omega_o, \omega_m, \omega_n, \omega_o)$, where all the frequencies are optical. In contrast, we require $\chi^{(3)}(\omega_m + \omega_n, \omega_m, \omega_n, 0)$, which involves one DC field. Since the optical frequency ω_o is vastly different from DC, inferring the value of $\chi^{(3)}(\omega_m + \omega_n, \omega_m, \omega_n, 0)$ from the values of $\chi^{(3)}(\omega_m + \omega_n + \omega_o, \omega_m, \omega_n, \omega_o)$ in the literature is difficult.

This difficulty can be intuitively understood by considering the example of $\chi^{(2)}$ nonlinearity. For a material with a native $\chi^{(2)}$ nonlinearity, the strengths of the optical nonlinearity and the electro-optic effect are given by $\chi^{(2)}(\omega_m + \omega_n, \omega_m, \omega_n)$ and $\chi^{(2)}(\omega_m, \omega_m, 0)$, respectively. The former is not generally a good indicator of the latter, or vice versa. For example, III-V semiconductor materials exhibit very large optical nonlinearities but only moderate electro-optic coefficients. On the other hand, for example, lithium niobate has a smaller nonlinear-optical coefficient and larger electro-optic coefficient than typical III-V materials.

S13. METHODS FOR INVERSE DESIGN

In this section, we discuss methods for training a programmable nonlinear waveguide to achieve the desired function. Formally, the behavior of a photonic device, including a programmable nonlinear waveguide, can be described by an input–output mapping

$$\mathbf{y} = f_{\mathbf{w}}(\mathbf{x}), \tag{S94}$$

where the vectors $\mathbf{x}$ and $\mathbf{y}$ denote the optical fields of the input and output, respectively, and $f_{\mathbf{w}}$ is a generally nonlinear function parameterized by the device parameters $\mathbf{w}$. The performance of the device is characterized by a loss function

$$\mathcal{L}(\bar{\mathbf{y}}, \mathbf{y}), \tag{S95}$$

which quantifies the deviation of the output $\mathbf{y}$ from the desired output $\bar{\mathbf{y}}$ for a given input $\mathbf{x}$. When the desired input–output mapping is perfectly implemented, $\mathcal{L} = 0$.

Inverse design is a powerful paradigm in which one first specifies the desired device function by choosing an appropriate loss function $\mathcal{L}$ and then searches for the optimal device parameters $\mathbf{w}$ that minimize $\mathcal{L}$, thereby realizing the desired function. As demonstrated in various works in photonics [S69, S70], highly nontrivial functions can be obtained via inverse design, especially for devices with many degrees of freedom that are challenging to optimize manually.

In the spectral engineering experiment presented in Fig. 3(b) in the main text, we implemented an inverse design by defining $\mathcal{L}$ as the distance between the desired and experimentally measured SH spectra. In this case, the parameters $\mathbf{w}$ were represented by 20 scalar values that parameterized the pattern of the programming illumination. As shown in the main text and Sec. S9, this approach enabled us to identify complex QPM grating structures that yielded highly nontrivial SH spectra.

A key distinction between our approach and conventional inverse design is that our approach is performed *in situ* and in real time on the basis of experimental feedback—an advantage that is uniquely enabled by the programmability of the device. In contrast, conventional inverse design is performed *in silico* (via digital simulations), and the resulting design is then implemented during fabrication. Below, we review these two paradigms of inverse design, discussing their advantages and limitations.

A. In silico inverse design

If we have a digital model $\tilde{f}_{\mathbf{w}}$ that accurately approximates the behavior of a device $f_{\mathbf{w}}$, we can optimize $\mathbf{w}$ much more efficiently via gradient-based methods. Specifically, we apply the chain rule to calculate the gradient of the loss function with respect to the parameters, i.e.,

$$\frac{\partial \mathcal{L}(\bar{\mathbf{y}}, \tilde{f}_{\mathbf{w}}(\mathbf{x}))}{\partial \mathbf{w}}, \tag{S96}$$

which indicates the direction in which the loss decreases. For example, a simple gradient descent algorithm uses the update rule

$$\mathbf{w} \mapsto \mathbf{w} - \alpha \, \frac{\partial \mathcal{L}(\bar{\mathbf{y}}, \tilde{f}_{\mathbf{w}}(\mathbf{x}))}{\partial \mathbf{w}}, \tag{S97}$$

where α is the learning rate. More sophisticated algorithms may further improve the optimization performance [S71].

Note that these optimizations are performed *in silico*, i.e., entirely digitally. An optimal $\mathbf{w}$ found through this approach is expected to perform well when deployed on a real physical device if our digital model $\tilde{f}_{\mathbf{w}}$ accurately reflects reality. Conversely, any discrepancy between the digital model and reality results in suboptimal device performance. In this sense, the paradigm of in silico inverse design faces inherent challenges in handling fabrication imperfections, miscalibration of the experimental setup, and environmental drift.

B. Hybrid in situ–in silico inverse design

In the paradigm of in situ inverse design, we optimize the device parameters on the basis of real-time feedback from the actual device. The simplest in situ inverse design method employed in this work is random optimization [S72]. In

each iteration, we update the parameters as follows:

$$\mathbf{w} \mapsto \mathbf{w} + \delta\mathbf{w}, \tag{S98}$$

where $\delta\mathbf{w}$ is randomly generated; we then experimentally measure the loss function $\mathcal{L}$. If the update reduces the loss relative to the previous value, we accept the new parameters; otherwise, we retain the previous parameters and proceed to the next iteration. A key advantage of this approach is that it does not require any prior knowledge of $f_{\mathbf{w}}$, which increases the robustness of the optimization. However, the optimization process tends to slow as the dimensionality of $\mathbf{w}$ increases.

Physics-aware training (PAT) is a hybrid in situ–in silico training method that enables efficient gradient-based optimization and leverages the robustness provided by experimental feedback [S73]. In PAT, similar to in silico inverse design, we construct a digital differential numerical model $\tilde{f}_{\mathbf{w}}$ that mimics the input–output map of the system. However, instead of using the digital model for the forward path, we use the actual physical system to obtain an experimental result and calculate the error vector $\frac{\partial \mathcal{L}}{\partial \mathbf{y}}$. The digital model $\tilde{f}_{\mathbf{w}}$ is then used to backpropagate the error and compute the gradient $\frac{\partial \mathcal{L}}{\partial \mathbf{w}}$.

[S1] D. D. Hickstein, D. R. Carlson, H. Mundoor, J. B. Khurgin, K. Srinivasan, D. Westly, A. Kowligy, I. I. Smalyukh, S. A. Diddams, and S. B. Papp, Self-organized nonlinear gratings for ultrafast nanophotonics, Nature Photonics **13**, 494–499 (2019).

[S2] A. Billat, D. Grassani, M. H. P. Pfeiffer, S. Kharitonov, T. J. Kippenberg, and C.-S. Brès, Large second harmonic generation enhancement in Si_3N_4 waveguides by all-optically induced quasi-phase-matching, Nature Communications **8** (2017).

[S3] E. Nitiss, J. Hu, A. Stroganov, and C.-S. Brès, Optically reconfigurable quasi-phase-matching in silicon nitride microresonators, Nature Photonics **16**, 134–141 (2022).

[S4] X. Lu, G. Moille, A. Rao, D. A. Westly, and K. Srinivasan, Efficient photoinduced second-harmonic generation in silicon nitride photonics, Nature Photonics **15**, 131–136 (2020).

[S5] B. Li, Z. Yuan, J. Williams, W. Jin, A. Beckert, T. Xie, J. Guo, A. Feshali, M. Paniccia, A. Faraon, J. Bowers, A. Marandi, and K. Vahala, Down-converted photon pairs in a high-Q silicon nitride microresonator, Nature **639**, 922 (2025).

[S6] G. Wang, O. Yakar, X. Ji, M. Clementi, J. Zhou, C. Lafforgue, J. Wu, J. Hu, T. J. Kippenberg, and C.-S. Brès, Integrated tunable green light source on silicon nitride, arXiv preprint arXiv:2504.13662.

[S7] Z. Yuan, J. Ge, P. Liu, B. Li, M. Li, J.-Y. Liu, Y. Yu, H.-J. Chen, J. Bowers, and K. Vahala, Efficient and wavelength-tunable second-harmonic generation toward the green gap, Science Advances **11**, eadw2781 (2025).

[S8] H.-H. Lin, R. Sharma, A. Friedman, B. M. Cromey, F. Vallini, M. W. Puckett, K. Kieu, and Y. Fainman, On the observation of dispersion in tunable second-order nonlinearities of silicon-rich nitride thin films, APL Photonics **4**, 036101 (2019).

[S9] E. Timurdogan, C. V. Poulton, M. J. Byrd, and M. R. Watts, Electric field-induced second-order nonlinear optical effects in silicon waveguides, Nature Photonics **11**, 200–206 (2017).

[S10] C. J. Krückel, A. Fülöp, Z. Ye, P. A. Andrekson, and V. Torres-Company, Optical bandgap engineering in nonlinear silicon nitride waveguides, Optics Express **25**, 15370 (2017).

[S11] C. Meier, A. Gondorf, S. Lüttjohann, A. Lorke, and H. Wiggers, Silicon nanoparticles: Absorption, emission, and the nature of the electronic bandgap, Journal of Applied Physics **101** (2007).

[S12] Y. Yang, A. Forbes, and L. Cao, A review of liquid crystal spatial light modulators: devices and applications, Opto-Electronic Science **2**, 230026 (2023).

[S13] M. A. Guidry, K. Y. Yang, D. M. Lukin, A. Markosyan, J. Yang, M. M. Fejer, and J. Vučković, Optical parametric oscillation in silicon carbide nanophotonics, Optica **7**, 1139 (2020).

[S14] X. Ji, S. Roberts, M. Corato-Zanarella, and M. Lipson, Methods to achieve ultra-high quality factor silicon nitride resonators, APL Photonics **6** (2021).

[S15] H. Jung, S.-P. Yu, D. R. Carlson, T. E. Drake, T. C. Briles, and S. B. Papp, Tantala kerr nonlinear integrated photonics, Optica **8**, 811 (2021).

[S16] Y. Zou, S. Chakravarty, C.-J. Chung, X. Xu, and R. T. Chen, Mid-infrared silicon photonic waveguides and devices [invited], Photonics Research **6**, 254 (2018).

[S17] T. Onodera, M. M. Stein, B. A. Ash, M. M. Sohoni, M. Bosch, R. Yanagimoto, M. Jankowski, T. P. McKenna, T. Wang, G. Shvets, M. R. Shcherbakov, L. G. Wright, and P. L. McMahon, Scaling on-chip photonic neural processors using arbitrarily programmable wave propagation, arXiv preprint arXiv:2402.17750.

[S18] A. M. Weiner, Ultrafast optical pulse shaping: A tutorial review, Optics Communications **284**, 3669 (2011).

[S19] Y. Liu, S.-G. Park, and A. Weiner, Terahertz waveform synthesis via optical pulse shaping, IEEE Journal of Selected Topics in Quantum Electronics **2**, 709 (1996).

[S20] Z. Jiang, D. Leaird, and A. Weiner, Line-by-line pulse shaping control for optical arbitrary waveform generation, Optics Express **13**, 10431 (2005).

[S21] http://www.optophase.com/images/Biophotonic\%20solutions/MIIPSBox640_sheet.pdf, accessed: 2025-07-08.
[S22] S. Divitt, W. Zhu, C. Zhang, H. J. Lezec, and A. Agrawal, Ultrafast optical pulse shaping using dielectric metasurfaces, Science **364**, 890 (2019).
[S23] https://amplitude-laser.com/products/femtosecond-lasers/instrumentation-lasers-femtosecondes/dazzler/, accessed: 2025-07-08.
[S24] A. J. Metcalf, H.-J. Kim, D. E. Leaird, J. A. Jaramillo-Villegas, K. A. McKinzie, V. Lal, A. Hosseini, G. E. Hoefler, F. Kish, and A. M. Weiner, Integrated line-by-line optical pulse shaper for high-fidelity and rapidly reconfigurable rf-filtering, Optics Express **24**, 23925 (2016).
[S25] G. Imeshev, A. Galvanauskas, D. Harter, M. A. Arbore, M. Proctor, and M. M. Fejer, Engineerable femtosecond pulse shaping by second-harmonic generation with Fourier synthetic quasi-phase-matching gratings, Optics Letters **23**, 864 (1998).
[S26] R. Shiloh and A. Arie, Spectral and temporal holograms with nonlinear optics, Optics Letters **37**, 3591 (2012).
[S27] A. Leshem, R. Shiloh, and A. Arie, Experimental realization of spectral shaping using nonlinear optical holograms, Optics Letters **39**, 5370 (2014).
[S28] J. Liu, G. Huang, R. N. Wang, J. He, A. S. Raja, T. Liu, N. J. Engelsen, and T. J. Kippenberg, High-yield, wafer-scale fabrication of ultralow-loss, dispersion-engineered silicon nitride photonic circuits, Nature Communications **12**, 2236 (2021).
[S29] J. F. Bauters, M. J. Heck, D. D. John, J. S. Barton, C. M. Bruinink, A. Leinse, R. G. Heideman, D. J. Blumenthal, and J. E. Bowers, Planar waveguides with less than 0.1 db/m propagation loss fabricated with wafer bonding, Optics express **19**, 24090 (2011).
[S30] P. Kumar, Quantum frequency conversion, Optics letters **15**, 1476 (1990).
[S31] S.-H. Wei, B. Jing, X.-Y. Zhang, J.-Y. Liao, C.-Z. Yuan, B.-Y. Fan, C. Lyu, D.-L. Zhou, Y. Wang, G.-W. Deng, *et al.*, Towards real-world quantum networks: a review, Laser & Photonics Reviews **16**, 2100219 (2022).
[S32] J. Zhao, Y. Xu, X. Lu, E. Kaur, M. Kilzer, R. Kompella, R. W. Boyd, and R. Nejabati, Scalable mhz-rate entanglement distribution in low-latency quantum networks interconnecting heterogeneous quantum processors, arXiv preprint arXiv:2504.05567 (2025).
[S33] T. F. Weiss and A. Peruzzo, Nonlinear domain engineering for quantum technologies, Applied Physics Reviews **12** (2025).
[S34] https://quantumcomputinginc.com/products/commercial-products/frequency-converter, accessed: 2025-07-10.
[S35] Y. M. Sua, H. Fan, A. Shahverdi, J.-Y. Chen, and Y.-P. Huang, Direct generation and detection of quantum correlated photons with 3.2 um wavelength spacing, Scientific reports **7**, 17494 (2017).
[S36] J. Lu, M. Li, C.-L. Zou, A. Al Sayem, and H. X. Tang, Toward 1% single-photon anharmonicity with periodically poled lithium niobate microring resonators, Optica **7**, 1654 (2020).
[S37] J. Lu, A. Al Sayem, Z. Gong, J. B. Surya, C.-L. Zou, and H. X. Tang, Ultralow-threshold thin-film lithium niobate optical parametric oscillator, Optica **8**, 539 (2021).
[S38] R. Yanagimoto, E. Ng, M. Jankowski, H. Mabuchi, and R. Hamerly, Temporal trapping: a route to strong coupling and deterministic optical quantum computation, Optica **9**, 1289 (2022).
[S39] X. Ji, F. A. Barbosa, S. P. Roberts, A. Dutt, J. Cardenas, Y. Okawachi, A. Bryant, A. L. Gaeta, and M. Lipson, Ultra-low-loss on-chip resonators with sub-milliwatt parametric oscillation threshold, Optica **4**, 619 (2017).
[S40] C. Couteau, Spontaneous parametric down-conversion, Contemporary Physics **59**, 291 (2018).
[S41] C. J. Xin, J. Mishra, C. Chen, D. Zhu, A. Shams-Ansari, C. Langrock, N. Sinclair, F. N. C. Wong, M. M. Fejer, and M. Lončar, Spectrally separable photon-pair generation in dispersion engineered thin-film lithium niobate, Optics Letters **47**, 2830 (2022).
[S42] V. Ansari, J. M. Donohue, B. Brecht, and C. Silberhorn, Tailoring nonlinear processes for quantum optics with pulsed temporal-mode encodings, Optica **5**, 534 (2018).
[S43] J.-L. Zhu, W.-X. Zhu, X.-T. Shi, C.-T. Zhang, X. Hao, Z.-X. Yang, and R.-B. Jin, Design of mid-infrared entangled photon sources using lithium niobate, Journal of the Optical Society of America B **40**, A9 (2023).
[S44] A. Fedrizzi, T. Herbst, A. Poppe, T. Jennewein, and A. Zeilinger, A wavelength-tunable fiber-coupled source of narrowband entangled photons, Optics Express **15**, 15377 (2007).
[S45] L. Shen, J. Lee, A. W. Hartanto, P. Tan, and C. Kurtsiefer, Wide-range wavelength-tunable photon-pair source for characterizing single-photon detectors, Optics Express **29**, 3415 (2021).
[S46] V. Sultanov, A. Kavčič, E. Kokkinakis, N. Sebastián, M. V. Chekhova, and M. Humar, Tunable entangled photon-pair generation in a liquid crystal, Nature **631**, 294–299 (2024).
[S47] A. Villar, A. Lohrmann, and A. Ling, Experimental entangled photon pair generation using crystals with parallel optical axes, Optics express **26**, 12396 (2018).
[S48] N. A. Harper, E. Y. Hwang, R. Sekine, L. Ledezma, C. Perez, A. Marandi, and S. K. Cushing, Highly efficient visible and near-IR photon pair generation with thin-film lithium niobate, Optica Quantum **2**, 103 (2024).
[S49] https://www.thorlabs.com/newgrouppage9.cfm?objectgroup_id=13675 (), accessed: 2025-07-10.
[S50] https://www.ozoptics.com/ALLNEW_PDF/DTS0199.pdf (), accessed: 2025-07-10.
[S51] https://s-fifteen.com/products/cpps-810-correlated-photon-pair-source (), accessed: 2025-07-10.
[S52] J. Schneeloch, S. H. Knarr, D. F. Bogorin, M. L. Levangie, C. C. Tison, R. Frank, G. A. Howland, M. L. Fanto, and P. M. Alsing, Introduction to the absolute brightness and number statistics in spontaneous parametric down-conversion, Journal of Optics **21**, 043501 (2019).
[S53] A. S. Solntsev, A. A. Sukhorukov, D. N. Neshev, and Y. S. Kivshar, Spontaneous parametric down-conversion and quantum walks in arrays of quadratic nonlinear waveguides, Physical Review Letters **108**, 023601 (2012).

[S54] J. G. Titchener, A. S. Solntsev, and A. A. Sukhorukov, Reconfigurable cluster-state generation in specially poled nonlinear waveguide arrays, Physical Review A **101**, 023809 (2020).

[S55] D. Barral, M. Walschaers, K. Bencheikh, V. Parigi, J. A. Levenson, N. Treps, and N. Belabas, Quantum state engineering in arrays of nonlinear waveguides, Physical Review A **102**, 043706 (2020).

[S56] B. Cao, K. Hayama, S. Suezawa, M. Hisamitsu, K. Tokuda, S. Kurimura, R. Okamoto, and S. Takeuchi, Non-collinear generation of ultra-broadband parametric fluorescence photon pairs using chirped quasi-phase matching slab waveguides, Optics Express **31**, 23551 (2023).

[S57] P.-K. Chen, I. Briggs, C. Cui, L. Zhang, M. Shah, and L. Fan, Adapted poling to break the nonlinear efficiency limit in nanophotonic lithium niobate waveguides, Nature Nanotechnology **19**, 44–50 (2023).

[S58] T. Sylvestre, E. Genier, A. N. Ghosh, P. Bowen, G. Genty, J. Troles, A. Mussot, A. C. Peacock, M. Klimczak, A. M. Heidt, J. C. Travers, O. Bang, and J. M. Dudley, Recent advances in supercontinuum generation in specialty optical fibers [invited], Journal of the Optical Society of America B **38**, F90 (2021).

[S59] J. R. Nagel, Numerical solutions to poisson equations using the finite-difference method [education column], IEEE Antennas and Propagation Magazine **56**, 209–224 (2014).

[S60] I. H. Malitson, Interspecimen comparison of the refractive index of fused silica, Journal of the Optical Society of America **55**, 1205 (1965).

[S61] L. Wang, W. Xie, D. Van Thourhout, Y. Zhang, H. Yu, and S. Wang, Nonlinear silicon nitride waveguides based on pecvd deposition platform, Optics Express **26**, 9645 (2018).

[S62] F. Ay and A. Aydinli, Comparative investigation of hydrogen bonding in silicon based pecvd grown dielectrics for optical waveguides, Optical Materials **26**, 33 (2004).

[S63] J. Hu and C. R. Menyuk, Understanding leaky modes: slab waveguide revisited, Advances in Optics and Photonics **1**, 58 (2009).

[S64] D. Bose, M. W. Harrington, A. Isichenko, K. Liu, J. Wang, N. Chauhan, Z. L. Newman, and D. J. Blumenthal, Anneal-free ultra-low loss silicon nitride integrated photonics, Light: Science & Applications **13**, 156 (2024).

[S65] X. Ji, Y. Okawachi, A. Gil-Molina, M. Corato-Zanarella, S. Roberts, A. L. Gaeta, and M. Lipson, Ultra-low-loss silicon nitride photonics based on deposited films compatible with foundries, Laser & Photonics Reviews **17**, 2200544 (2023).

[S66] S. Tan, Z. Zhao, K. Urbanek, T. Hughes, Y. J. Lee, S. Fan, J. S. Harris, and R. L. Byer, Silicon nitride waveguide as a power delivery component for on-chip dielectric laser accelerators, Optics letters **44**, 335 (2019).

[S67] M. Jankowski, R. Yanagimoto, E. Ng, R. Hamerly, T. P. McKenna, H. Mabuchi, and M. M. Fejer, Ultrafast second-order nonlinear photonics—from classical physics to non-gaussian quantum dynamics: a tutorial, Advances in Optics and Photonics **16**, 347 (2024).

[S68] R. W. Boyd, *Nonlinear Optics* (Academic Press, 2008).

[S69] C.-Y. Lee, Y. Liu, Y. Cheng, C. Lao, and Q.-F. Yang, Inverse design of coherent supercontinuum generation using free-form nanophotonic waveguides, APL Photonics **9** (2024).

[S70] S. Molesky, Z. Lin, A. Y. Piggott, W. Jin, J. Vucković, and A. W. Rodriguez, Inverse design in nanophotonics, Nature Photonics **12**, 659–670 (2018).

[S71] D. P. Kingma and J. Ba, Adam: A method for stochastic optimization, in *3rd International Conference on Learning Representations, ICLR 2015, San Diego, CA, USA, May 7-9, 2015, Conference Track Proceedings*, edited by Y. Bengio and Y. LeCun (2015).

[S72] J. Matyas, Random optimization, Automation and Remote Control **26**, 246 (1965).

[S73] L. G. Wright, T. Onodera, M. M. Stein, T. Wang, D. T. Schachter, Z. Hu, and P. L. McMahon, Deep physical neural networks trained with backpropagation, Nature **601**, 549–555 (2022).
